# Luteinizing hormone activity in ovarian stimulation: comparative efficacy and safety of gonadotropins *versus* recombinant follicle-stimulating hormone—a systematic review and meta-analysis

**DOI:** 10.3389/fendo.2026.1792900

**Published:** 2026-04-22

**Authors:** Juan Jose Espinós, Miguel Angel Checa, Esteban Villegas-Arbelaez, Mª José Martínez-Zapata

**Affiliations:** 1 Fertty Foundation, Barcelona, Spain; 2 Universitat Autònoma de Barcelona (UAB), Barcelona, Spain; 3 Clinical Epidemiology and Public Health Service, Hospital de la Santa Creu i Sant Pau, Barcelona, Spain; 4 Institut de Recerca Sant Pau, Barcelona, Spain; 5 Centro de Investigación Biomédica en Red de Epidemiología y Salud Pública (CIBERESP), Madrid, Spain

**Keywords:** gonadotropins, HP-hMG, infertility, live birth, OHSS, ovarian stimulation, rFSH, rLH

## Abstract

**Background:**

Luteinizing hormone (LH) activity plays a crucial role in follicular development, endometrial decidualization, and embryo implantation. We aimed to compare the efficacy and safety of gonadotropins with LH activity *versus* recombinant follicle-stimulating hormone (rFSH) for controlled ovarian stimulation (COS).

**Methods:**

We included randomized clinical trials (RCTs) assessing gonadotropins with LH activity with or without rFSH *versus* rFSH, or comparing different sources of LH activity, or different doses. Participants were women undergoing COS. The outcomes were *live births*, *ongoing pregnancies*, *oocytes recovered per woman*, *metaphase II (MII) oocytes per woman*, and *occurrence of ovarian hyperstimulation syndrome (OHSS)*. We searched the MEDLINE, Embase, and CENTRAL databases. We calculated the mean differences (MDs) and risk ratios (RRs) for continuous and dichotomous outcomes, respectively.

**Results:**

We included 56 RCTs with 14,034 women. The evidence of gonadotropins with LH activity (±rFSH) when compared with rFSH alone probably results in little to no difference in live birth (RR = 1.07, 95%CI = 0.96–1.18; 17 studies, moderate certainty of evidence) and ongoing pregnancy (RR = 1.03, 95%CI = 0.95–1.12; 19 studies, moderate certainty of evidence), may result in a slight reduction in the number of oocytes retrieved (MD = −0.50, 95%CI = −0.88 to −0.12, *I*^2^ = 81.1%; 46 studies, low certainty of evidence), and is very uncertain in the number of MII oocytes (MD = −0.49, 95%CI = −0.93 to −0.05, *I*^2^ = 86%; 30 studies, very low certainty of evidence). However, it probably does not increase OHSS compared with rFSH alone (RR = 0.80, 95%CI = 0.61–1.03, *I*^2^ = 17.1%; 19 studies, moderate certainty of evidence). Subgroup analysis by downregulation protocol, ovarian reserve, and age showed some differences in the effects between the compared groups.

**Conclusions:**

The evidence of gonadotropins with LH activity (±rFSH) when compared with rFSH alone probably results in little to no difference in live birth and ongoing pregnancy. However, there is a slight reduction in the number of oocytes retrieved. Moreover, they are safer than rFSH alone because they probably do not increase OHSS. Therefore, based on our review, the benefit–risk balance favors the addition of an LH-active gonadotropin for the prevention of OHSS. More studies are needed to determine the effects in specific populations.

**Systematic Review Registration:**

https://www.crd.york.ac.uk/PROSPERO/view/CRD42024572731, identifier CRD42024572731.

## 1 Introduction

Gonadotropins constitute the cornerstone of controlled ovarian stimulation (COS) and are essential for the success of assisted reproductive technologies (ARTs), including *in vitro* fertilization (IVF), intracytoplasmic sperm injection (ICSI), and fertility preservation strategies. By regulating follicular recruitment and development, gonadotropins directly influence the oocyte yield, embryo quality, and, ultimately, the reproductive outcomes.

In humans, follicle-stimulating hormone (FSH) and luteinizing hormone (LH) are the two principal gonadotropins involved in ovarian function. Currently, three main types of gonadotropin preparations are available for clinical use in COS: recombinant human FSH (rFSH); highly purified human menopausal gonadotropin (HP-hMG), which provides urinary-derived FSH and LH activity at an approximately 1:1 ratio; and recombinant FSH and LH combinations, administered either as a fixed-ratio single formulation [2:1 rFSH/recombinant LH (rLH)] or as separate products allowing individualized dosing.

Recombinant gonadotropins are produced using recombinant DNA technology, ensuring high purity, molecular consistency, and minimal batch-to-batch variability. In contrast, HP-hMG is derived from the urine of postmenopausal women and contains both FSH and LH bioactivity. Advances in purification techniques have reduced the urinary protein contaminants to less than 5%, thereby minimizing the risk of rare adverse reactions such as local hypersensitivity.

Beyond its role in steroidogenesis, LH is critical for follicular maturation, endometrial receptivity, decidualization, and embryo implantation (1). Consequently, LH activity has been incorporated into COS protocols through the administration of hMG, HP-hMG, or rLH. Several studies suggest that adequate LH supplementation during COS may optimize the ovarian response and improve oocyte competence, particularly in selected patient populations (2, 3). For instance, Behre et al. (4) demonstrated that the addition of rLH to rFSH increased the clinical pregnancy rates per cycle start and per embryo transfer in women of advanced reproductive age. Observational data further indicate that, in women with low or suboptimal ovarian response, combined FSH and LH administration may improve oocyte quality, resulting in pregnancy and delivery rates comparable to those of women with normal ovarian response (2).

Despite these findings, the clinical benefit of LH supplementation remains controversial. A recent systematic review by Mao et al. (5) comparing rFSH plus rLH with rFSH alone reported significantly greater endometrial thickness in the combination group, but fewer retrieved oocytes and high-quality embryos. Similarly, the systematic review by Orvieto et al. (3), which included 11 studies (only two randomized controlled trials), found no significant differences between rFSH plus rLH and hMG in terms of oocyte yield, oocyte maturity, ongoing pregnancy, or live birth rates, although a higher incidence of ovarian hyperstimulation syndrome (OHSS) was observed in the rFSH plus rLH group. Other systematic reviews have reported heterogeneous results, with some suggesting a potential increase in ongoing pregnancy rates with rLH supplementation (6), while others found age-dependent differences when comparing rFSH plus rLH with hMG, particularly in women over 30 years of age (7).

Comparisons between rFSH and HP-hMG have also yielded inconsistent results. An early systematic review by Al-Inany et al. (8) reported significantly higher ongoing pregnancy and live birth rates in favor of HP-hMG in IVF cycles. However, more recent evidence, including studies in women with polycystic ovary syndrome (PCOS) undergoing intrauterine insemination, failed to demonstrate significant differences in the reproductive outcomes between rFSH and hMG or HP-hMG (9, 10). Moreover, while rFSH has been associated with a higher number of retrieved oocytes, this advantage has not consistently translated into improved pregnancy or live birth rates.

Given the heterogeneity of the study designs, patient populations, and COS protocols, the optimal use of gonadotropins with LH activity remains uncertain. Therefore, this systematic review aimed to update and clarify the current evidence regarding the efficacy and safety of gonadotropin preparations containing LH activity—specifically rLH and HP-hMG—in COS. A secondary objective was to evaluate their effectiveness in clinically relevant subgroups, including women of advanced maternal age, those with diminished ovarian reserve, and patients undergoing different pituitary downregulation protocols.

## 2 Methods

We followed standard methods for the conduct of systematic reviews of interventions (11) and reported the review following the Preferred Reporting Items for Systematic Reviews and Meta-Analyses (PRISMA) guidelines. The protocol was published at the PROSPERO database (12).

### 2.1 Eligibility criteria

We included randomized clinical trials (RCTs) comparing the safety and efficacy of adding LH activity to rFSH (HP-hMG *vs*. rFSH; HP-hMG + rFSH *vs*. rFSH; and rLH + rFSH *vs*. rFSH) (comparison 1); comparing the LH activity sources: HP-hMG *versus* synthetic gonadotropins as HP-hMG *vs*. rLH + rFSH and HP-hMG + rFSH *vs*. rLH + rFSH (comparison 2); and other comparisons such as HP-hMG *vs*. HP-hMG + rFSH or comparisons of different doses of gonadotropin with LH activity + rFSH. The included participants were women undergoing ovarian stimulation for the development of multiple follicles as part of IVF, ISCI, or fertility preservation and women acting as oocyte donors.

We excluded academic abstracts presented at congresses, observational studies (e.g., cohorts, real-life studies, exploitation of large registries, case–control, and cross-sectional studies), and studies involving ovarian stimulation for intrauterine insemination.

The outcomes of interest were the efficacy and safety of the treatments assessed. The efficacy outcomes were the number or percentage of live births (delivery of a live fetus after at least 20 complete weeks of gestation); the number or percentage of ongoing pregnancies, defined as detection of a gestational sac with fetal heartbeat at 12 weeks or more (confirmed via ultrasound); the number of oocytes retrieved per woman; and the number of metaphase II (MII) oocytes per woman.

The safety outcomes were the number or percentage of OHSS cases, as defined by the study authors, with moderate or severe intensity.

### 2.2 Information sources and search strategy

We designed systematic and comprehensive bibliographic searches according to current standards for conducting systematic reviews (13). We report the complete searches according to the PRISMA-S statement (14).

An information specialist designed specific search strategies for each bibliographic database considered. We searched the following bibliographic databases to the most recent data: MEDLINE (accessed via PubMed; October 22, 2025), Embase (via ; March 1, 2024), and CENTRAL (via The Cochrane Library; October 17, 2025). The literature search string for each database is presented in the *Annex*.

### 2.3 Selection process

We used EndNote X20 (The EndNote Team, 2023, Clarivate, Philadelphia, PA, USA) to create a reference database to manage the search results. We removed duplicates (records published in the same journal, volume, number, and pages) and constructed a database with unique records using the Covidence web-based software for managing the eligibility process (www.covidence.org).

Based on the literature search and the inclusion and exclusion criteria established for the systematic review, two researchers with expertise in systematic reviews independently selected references in duplicate based on reading the title and abstract. A screening based on the full text was conducted by two researchers independently. Discrepancies were resolved by discussion, with the input of content experts if necessary.

### 2.4 Data collection process

One researcher with expertise in the conduct of systematic reviews, with a methodological profile, carried out the following stages for the included studies: i) critical reading of the selected studies; ii) preparation of descriptive tables; and iii) data extraction from the primary studies. A second researcher reviewed the extracted data, and any errors or discrepancies were discussed and agreed upon for correction.

### 2.5 Data items

The main characteristics of the included studies are reported in a tabulated format, including: author and publication year, study design, country, study period, description of participants (e.g., number and age), prognostic response profile, risk of bias (RoB) items, description of the gonadotropin regimen and cycle characteristics [e.g., the type of gonadotropin-releasing hormone (GnRH) downregulated cycle, the FSH/LH ratio, the initial dose of gonadotropin, the total gonadotropin dose (separately for rFSH and for LH) if adjustment of the initial dose, day of LH start (either alone or as HP-hMG), and the duration of gonadotropin drugs until hCG], the outcome data (e.g., the number of live births and ongoing pregnancies, the mean and standard deviation of the oocytes retrieved, and the number of participants with OHSS), and funding.

### 2.6 Study risk of bias assessment

A researcher with expertise in the conduct of systematic reviews, with a methodological profile, assessed the RoB of the included studies. A second researcher reviewed the assessments, and any errors or discrepancies were discussed and agreed upon for correction.

RoB was assessed using the Cochrane risk-of-bias tool version 2 (RoB 2) for randomized trials (15). The tool is structured in a set of bias domains, focusing on different aspects of trial design, conduct, and reporting. The five domains for individually randomized trials are: 1) bias arising from the randomization process; 2) bias due to deviations from the intended interventions; 3) bias due to missing outcome data; 4) bias in the measurement of the outcome; and 5) bias in the selection of the reported result. The perspective of the RoB assessment was that of assignment to intervention [intention-to-treat (ITT) effect]. For each domain, a RoB assessment of low, high, or some concerns is reached based on the answers to several signaling questions.

The domain “Bias due to deviations from the intended interventions” was assessed considering protocol deviations reported as such by the study authors. Cycle cancellation was addressed in that missing outcome domain, and changes in the gonadotropin dose or duration are not considered deviations from the intended intervention.

The domain “Bias due to missing outcome data” was generally rated as low risk for studies conducting an ITT or modified ITT (mITT) analysis for the outcome considered. A mITT analysis includes all randomized participants who received at least one dose of the intervention drug.

If a study did not conduct ITT or mITT, but an approximate ITT analysis was possible, then the domain was generally rated as low risk for the outcomes *live birth, ongoing pregnanc*y, and *OHSS*, but some concerns or high risk for the outcomes *number of oocytes* or *number of MII oocytes*.

Cycles canceled due to no response are a missing data bias for oocytes; however, cycles canceled due to hyper-response are NOT a bias for oocytes, only for OHSS.

The domain “Bias in the measurement of the outcome” was relevant only for OHSS, whose diagnosis and classification can be subjective. For the remaining outcomes, assessments are objective, and lack of blinding is unlikely to introduce bias; thus, the domain was generally rated as low risk.

An overall RoB judgment of low, high, or some concerns was reached for each study based on the assessments of RoB for the five domains. As a general rule, studies with all domains at low RoB were judged to be at an overall low RoB; studies with at least one domain at high RoB or presenting some concerns for multiple domains such that it substantially lowers confidence in the results were judged to be at an overall high RoB; otherwise, studies were considered to present some concerns of RoB.

### 2.7 Effect measures and synthesis methods

We obtained overall and stratified pooled estimates of the efficacy of different gonadotropin products on ovarian stimulation. Pooled estimates of impact were mean differences (MDs) with 95% confidence intervals (95%CI) for continuous outcomes, and risk ratios (RRs) with 95%CI for dichotomous outcomes were obtained using a random effects model with the Mantel–Haenszel or inverse variance method.

We assessed the presence of heterogeneity between studies by visual inspection of the forest plots for all outcomes, complemented with the assessment of the percentage of heterogeneity (*I*^2^ parameter) for relative effects (16). In addition, heterogeneity of 0%–40% was considered as “might not be important,” 30%–60% as “moderate heterogeneity,” 50%–90% as “substantial heterogeneity,” and 75%–100% as “considerable heterogeneity.” It should be noted that overlapping categories convey that there are no strict cutoffs for the interpretation of heterogeneity, and categorization depends on the magnitude and direction of the effects. The sources of heterogeneity were explored with subgroup and sensitivity analyses. In outcomes presenting considerable heterogeneity, appropriateness of meta-analysis was discussed and narrative synthesis considered. We performed analyses in Stata (StataCorp 2017, Stata Statistical Software: Release 15; StataCorp LLC, College Station, TX, USA) and Cochrane Review Manager [RevMan (computer program), version 5.4; The Cochrane Collaboration, 2020].

### 2.8 Unit of analysis issues

The outcomes are presented as per woman (or couple) randomized, regardless of the number of cycles per woman. If data per woman were not reported or could not be extracted, the study was not included in the meta-analysis.

### 2.9 Dealing with missing data

Data were reported and analyzed according to the ITT principle, wherever possible, extracting from the studies the outcome data for all randomized participants (ITT analysis) or for all randomized participants that received at least one dose of gonadotropins (mITT analysis). In studies with missing outcome data (e.g., due to withdrawal of participants or cycles canceled, among others), imputation of missing data was conducted when possible.

Live births, ongoing pregnancy, and OHSS were assumed not to have occurred in participants without a reported outcome. A sensitivity analysis was conducted for OHSS, creating a composite outcome of participants with OHSS and participants with cycles canceled due to hyper-response (risk of OHSS).

In studies where analyses for the outcomes “number of oocytes” or “number of MII oocytes” did not consider cycles canceled (e.g., due to no response or due to hyper-response), an approximate ITT analysis was conducted. To this aim, the mean number of oocytes including all randomized participants was recalculated by setting the values for participants with canceled cycles to zero and including these women in the divisor. This approximate analysis required also to impute the corresponding standard deviations, which were obtained using a previously reported method (17).

### 2.10 Assessment of reporting bias

When 10 or more studies were included in the meta-analysis, we assessed the reporting bias using a funnel plot for the live birth and OHSS rates.

### 2.11 Subgroup and sensitivity analyses

The synthesis results were stratified by ovarian reserve, age group, and the downregulating protocol (agonist or antagonist).

For ovarian reserve, we collected the definitions used by the study authors and matched them with the proposed categorization: G1 ovarian reserve when the antral follicle count (AFC) is <5 follicles and/or anti-Müllerian hormone (AMH) when <1.2 ng/ml; G2 ovarian reserve when AFC = 5–20 follicles and/or AMH = 1.2–4 ng/ml; and G3 ovarian reserve when AFC > 20 follicles and/or AMH > 4 ng/ml.

We considered stratifying by age group, younger (≤35 years) *vs*. older (>35 years) and, by dose in the comparison of HP-hMG and rFSH, 150 *vs*. 225 IU/day.

A sensitivity analysis was conducted defining an outcome that considered ongoing and clinical pregnancies. In studies reporting both ongoing and clinical pregnancy, the data for ongoing pregnancy were first considered. Furthermore, a sensitivity analysis was conducted analyzing the combined outcome “OHSS or risk of OHSS,” including those participants withdrawn from the study due to risk of OHSS.

### 2.12 Certainty assessment

Certainty of evidence was assessed using the GRADE system with the GRADEpro software (GRADEpro Guideline Development Tool, McMaster University and Evidence Prime, 2025; available from ). We rated the certainty of evidence across studies and for each outcome as high, moderate, low, or very low, depending on several factors including RoB, imprecision, inconsistency, indirectness, and publication bias (18).

The GRADE approach specifies four levels of quality: high quality—Further research is very unlikely to change our confidence in the estimate of effect; moderate quality—Further research is likely to have an important impact on our confidence in the estimate of effect and may change the estimate; low quality—Further research is very likely to have an important impact on our confidence in the estimate of effect and is likely to change the estimate; and very low quality—We are very uncertain about the estimate.

We included the following five key outcomes in the summary of the findings tables: i) *live birth rate*; ii) *ongoing pregnancy rate*; iii) *OHSS*; iv) *number of oocytes retrieved*; and v) *number of MII oocytes retrieved*.

## 3 Results

### 3.1 Study selection

**Figure 1:**
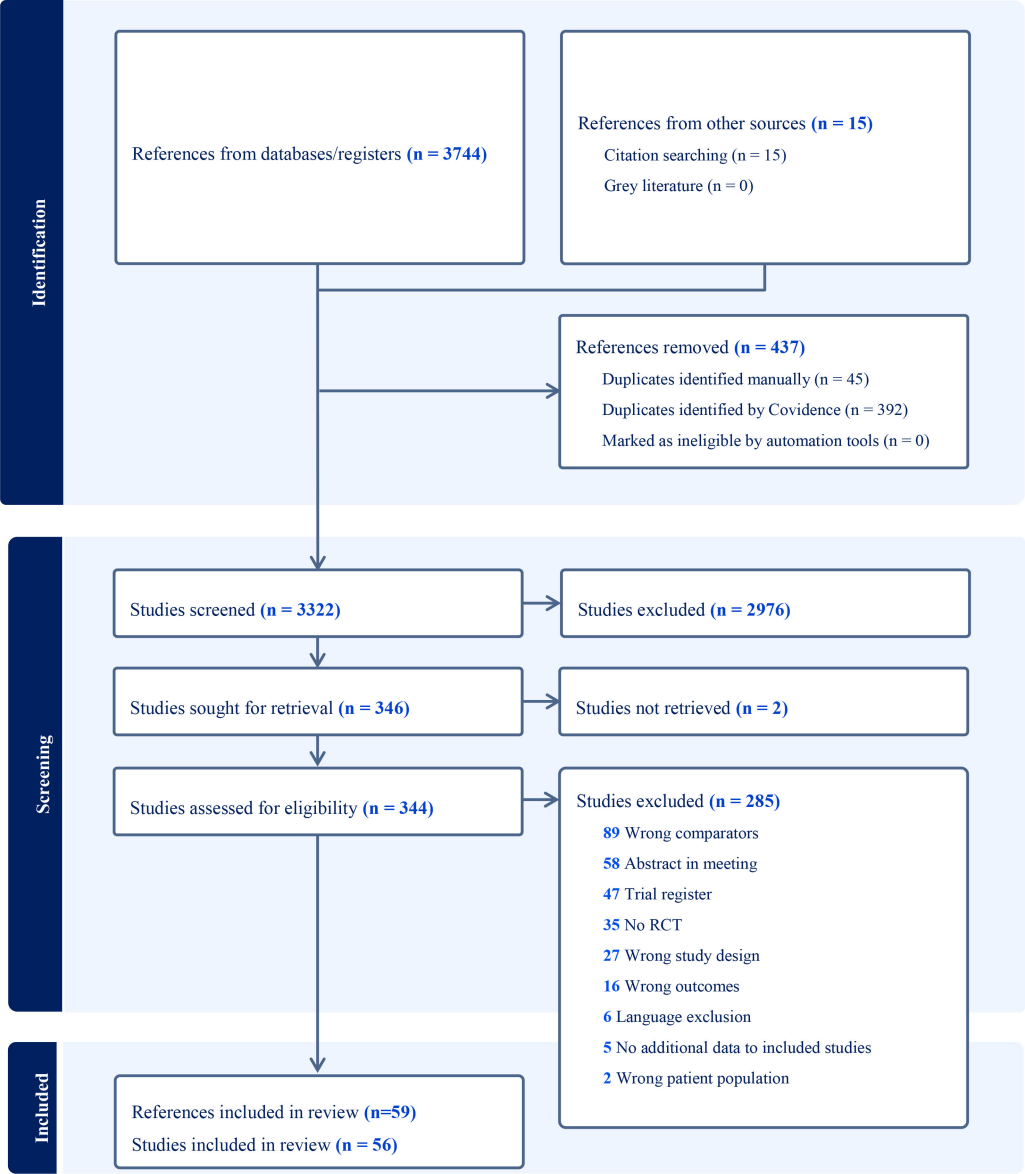
PRISMA 2020 flow diagram of study selection.

The searches retrieved 3,322 references, of which 344 were assessed by reading the full manuscript text, and 59 publications that corresponded to 56 RCTs were included, with a total of 14,034 women (**Figure 1**) (19–77). The reasons for the exclusion of 285 full-text studies are listed in **Supplementary Table 1**.

### 3.2 Study characteristics

**Supplementary Table 2** displays the descriptive characteristics of the included studies. The average sample size was 251 randomized participants (range = 20–1028). In 18 (32%) studies, the authors explicitly reported no conflicts of interest, while 12 (21%) studies reported non-financial funding and 15 (27%) studies reported funding or provision of services by interested companies.

All studies were conducted in women undergoing assisted reproductive treatments, except for two studies conducted in donors (24, 56). There were seven multinational studies. The remainder were single-country studies, with the majority conducted in Italy and Spain (*n* = 14 each), followed by Iran (*n* = 4); the Netherlands and China (*n* = 3 each); Denmark, Israel, and the United States (*n* = 2 each); and Australia, Brazil, France, Germany, Hungary, Turkey, and Vietnam (*n* = 1 each).

With respect to the indicators of poor ovarian reserve, there were 12 studies in women ≤35 years (24, 32, 33, 40, 48, 52, 66, 68–71, 74), 10 studies in women >35 years (22, 36–38, 42, 49, 55, 64, 73, 76), and five studies that reported results disaggregated by the participant’s age (25, 46, 54, 59, 60). The remaining studies included participants both younger and older than 35 years.

There were 10 studies conducted in women with low ovarian reserve or predicted low response (23, 34, 40, 41, 47, 58, 64, 72, 75, 77), while five of the studies were conducted in women with normal or good response (24, 30, 33, 69, 74), and one reported data separately by ovarian response (59). The remaining studies included a mix of participants with poor or good responses or did not report this characteristic.

### 3.3 Downregulation protocol

The downregulation protocols followed were agonist in 34 (60%) studies (21–23, 28, 31, 32, 35, 37, 38, 40, 42, 44–48, 50, 51, 53–56, 58–62, 66–71, 76), antagonist in 19 (34%) studies (24–26, 29, 30, 33, 34, 36, 39, 43, 49, 52, 57, 65, 72–75, 77), and a mixture in two studies where the participating units applied different protocols (41, 64). The protocol was not specified in one study (27). The downregulation agents applied were GnRH agonists such as triptorelin acetate (*n* = 16), leuprolide acetate (*n* = 10), buserelin (*n* = 9), and nafarelin (*n* = 1) and the GnRH antagonists cetrorelix (*n* = 11) and ganirelix (*n* = 8). The specific product was not specified in four studies.

### 3.4 Gonadotropin comparisons

The comparisons of the gonadotropin regimens studied are summarized in **Table 1**. The more frequent comparisons were the combination of rLH and rFSH compared with rFSH (33 studies and 6,747 women) and HP-hMG *vs*. rFSH (13 studies and 5,269 women).

**Table 1:** Distribution of the included studies by comparison of gonadotropin regimens.

Comparison	Studies (*N* = 56)	Total participants (*N* = 14,034)
Comparison 1: Addition of LH activity		
rLH + rFSH *vs*. rFSH	33	6,747
HP-hMG *vs*. rFSH	13 (16 publications)	5,269
HP-hMG + rFSH *vs*. rFSH	5	1,693
Comparison 2: Source of LH activity		
HP-hMG + rFSH *vs*. rLH + rFSH	2	234
HP-hMG *vs*. rLH + rFSH	2	157
Other comparisons		
HP-hMG + rFSH *vs*. HP-hMG	2	916
rLH^a^ + rFSH *vs*. rLH^a^ + rFSH	2	171

*LH*, luteinizing hormone; *HP-hMG*, highly purified human menopausal gonadotropin; *rFSH*, human follicle-stimulating hormone; *rLH*, recombinant luteinizing hormone.

a Comparison of different doses.

All RCTs contributed data to a single comparison, except that of Melo et al. (56), whose three arms were included in three comparisons, and Fábregues et al. (38), whose three arms were included in two comparisons. A few studies (31, 32, 41, 75) reported data for some study arms that were not included in the review due to the interventions in those arms not being of interest or the arm was not randomized.

A pre-randomization gonadotropin treatment with rFSH was administered in 16 studies (23, 29–32, 40, 44, 46, 49–52, 55, 62, 66, 67, 70, 71).

The commercial gonadotropin products used were as follows: for rFSH: Gonal-F^®^ (*n* = 44), Puregon^®^ (*n* = 4), Follistim^®^ (*n* = 1), and Elonva^®^ (*n* = 1); for HP-hMG: Menopur^®^ (*n* = 14), Merional^®^ (*n* = 3), Meriofert^®^ (*n* = 1), and hMG-Lepori^®^ (*n* = 1); and for rLH with or without rFSH: Luveris^®^ (*n* = 27) and Pergoveris^®^ (*n* = 3).

### 3.5 Risk of bias in the included studies

RoB was assessed for each group of outcomes: live birth/ongoing pregnancy, number of oocytes/MII oocytes, OHSS (see **Supplementary Figures 1–3**).

A total of 49 studies reported data for live birth and/or ongoing pregnancy. The RoB was low for 21 studies, some concerns for 25 studies, and high for three studies. The overall RoB was high mainly due to inadequate allocation concealment and no information on deviations from the intended intervention.

A total of 55 studies reported data for the number of oocytes and/or MII oocytes. The RoB was low for 10 studies, some concerns for 19 studies, and high for 26 studies. The overall RoB was high mainly due to inadequate allocation concealment, deviations from the intended intervention, and missing outcome data.

A total of 23 studies reported data for OHSS. The RoB was low for 12 studies, some concerns for nine studies, and high for two studies. The overall RoB was high mainly due to inadequate allocation concealment and no information on deviations from the intended interventions.

### 3.6 Results of syntheses

#### 3.6.1 Comparison 1: gonadotropins with LH (±rFSH) activity *vs*. rFSH alone

This comparison was informed by 42 studies: 13 studies comparing HP-hMG against rFSH, five studies comparing HP-hMG + rFSH against rFSH, and 25 studies comparing rLH + rFSH *vs*. rFSH.

##### 3.6.1.1 Live birth

**Figure 2:**
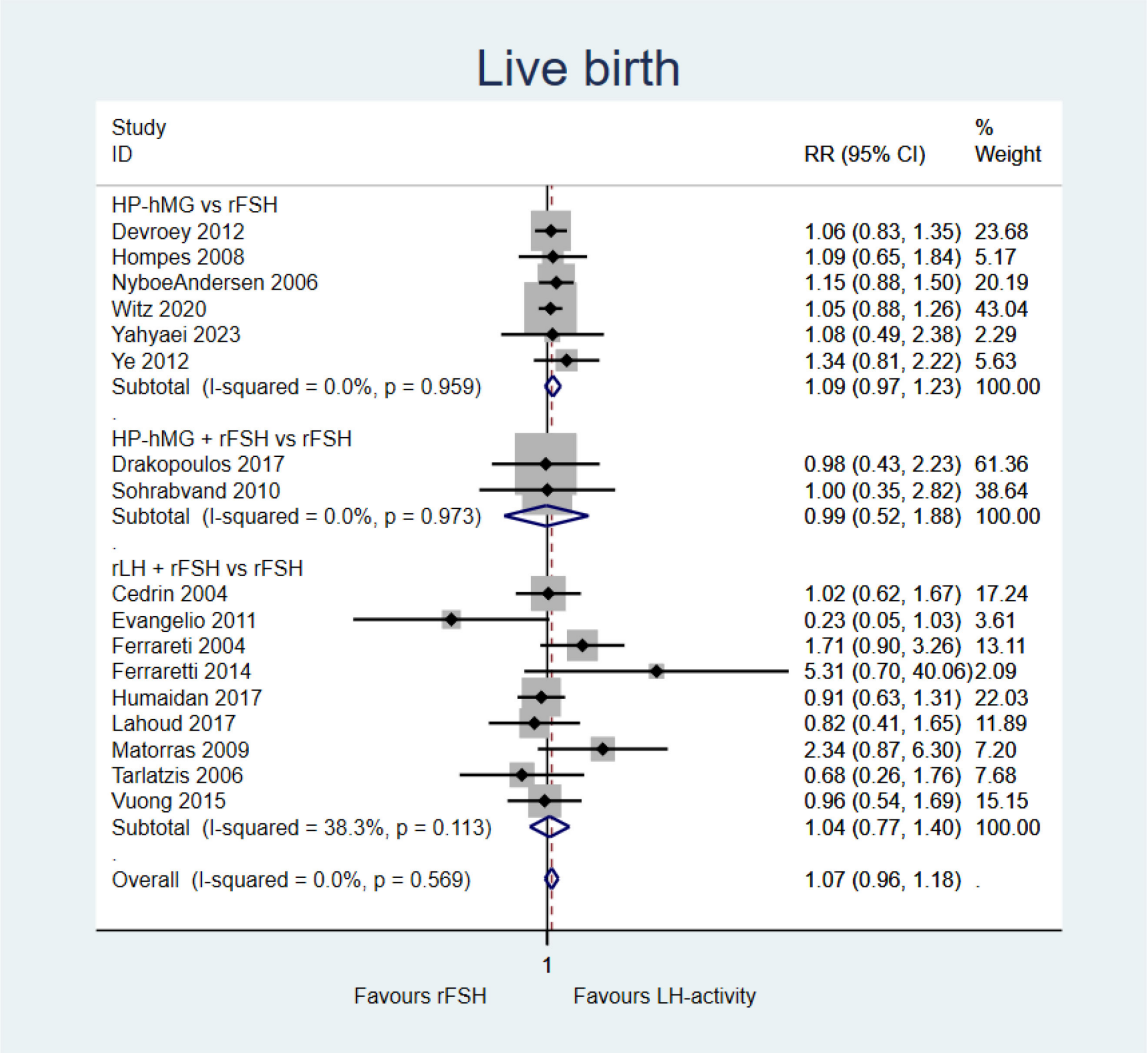
Forest plot for live birth.

Gonadotropins with LH activity (±rFSH) *versus* rFSH alone probably results in little to no difference in live birth (RR = 1.07, 95%CI = 0.96–1.18; 17 studies, moderate certainty of evidence) (**Figure 2** and Summary of finding in **Supplementary Table 4**). Comparisons of HP-hMG *vs*. rFSH (RR = 1.09, 95%CI = 0.97–1.23; six studies), adding rFSH to HP-hMG *vs.* rFSH (RR = 0.99, 95%CI = 0.52–1.88; two studies), and adding rLH to rFSH (RR = 1.04, 95%CI = 0.77–1.40; nine studies) presented similar results (**Figure 2**).

##### 3.6.1.2 Ongoing pregnancy

**Figure 3:**
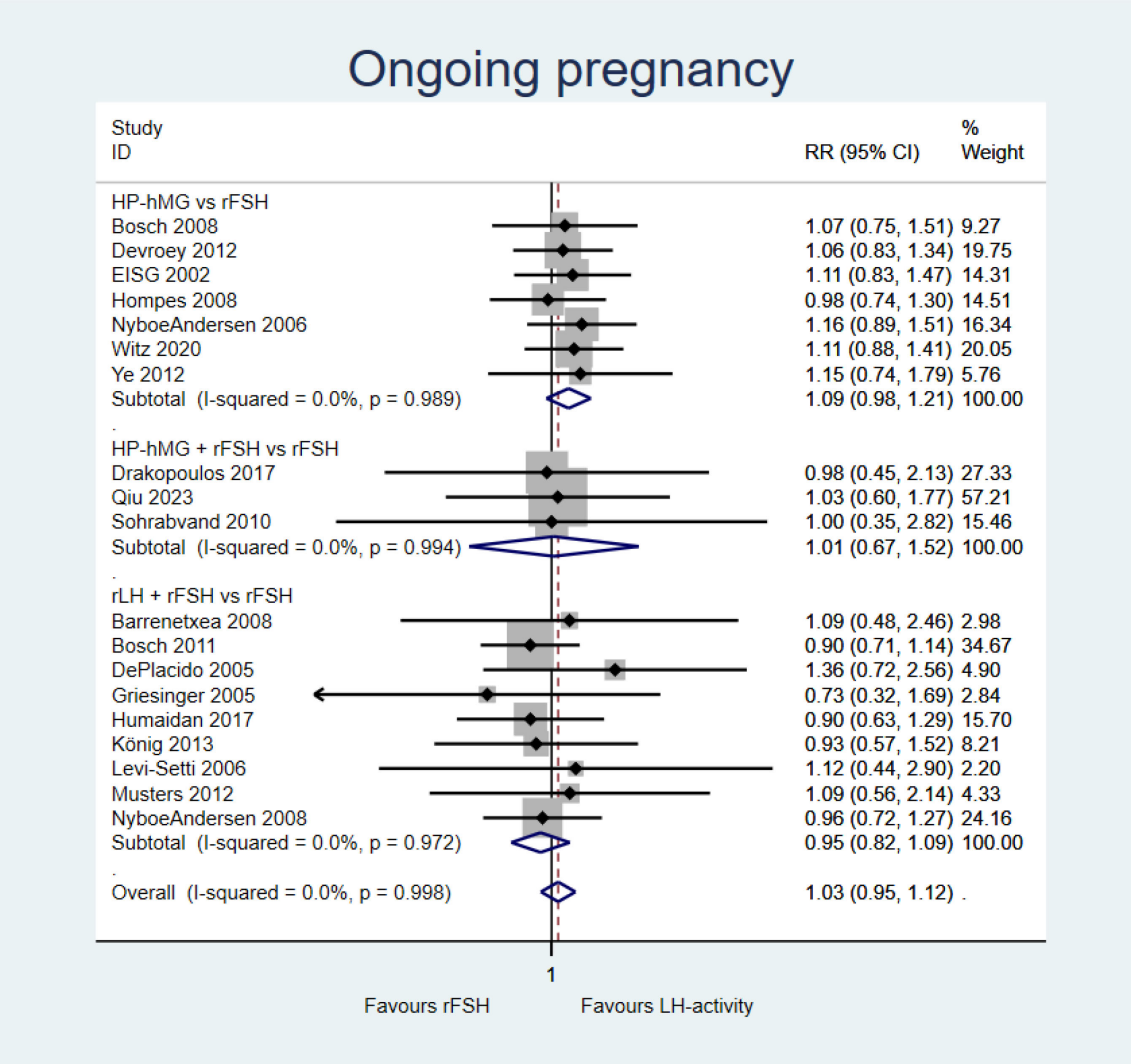
Forest plot for ongoing pregnancy.

Gonadotropins with LH activity (±rFSH) *versus* rFSH alone probably results in little to no difference in ongoing pregnancy (RR = 1.03, 95%CI = 0.95–1.12; 19 studies, moderate certainty of evidence) (**Figure 3**). Comparisons of HP-hMG *vs*. rFSH (RR = 1.09, 95%CI = 0.98–1.21; seven studies), adding rFSH to HP-hMG *vs.* rFSH (RR = 1.01, 95%CI = 0.67–1.52; three studies), and adding rLH to rFSH vs. rFSH alone (RR = 0.95, 95%CI = 0.82–1.09; nine studies) presented similar results (**Figure 3**).

A sensitivity analysis pooling studies that provided data for ongoing and clinical pregnancy outcomes showed that gonadotropins with LH activity (±rFSH) *versus* rFSH probably results in little to no difference for this outcome (RR = 1.02, 95%CI = 0.96–1.09, *I*^2^ = 0%; 46 studies, moderate certainty of evidence) (Summary of finding in **Supplementary Table 4**). Comparisons of HP-hMG *vs*. rFSH (RR = 1.06, 95%CI = 0.96–1.17; 10 studies), adding rFSH to HP-hMG *vs.* rFSH (RR = 1.14, 95%CI = 0.91–1.44; four studies), and adding rLH to rFSH (RR = 0.97, 95%CI = 0.89–1.07; 32 studies) presented similar results.

##### 3.6.1.3 Number of oocytes retrieved (PP analysis)

**Figure 4:**
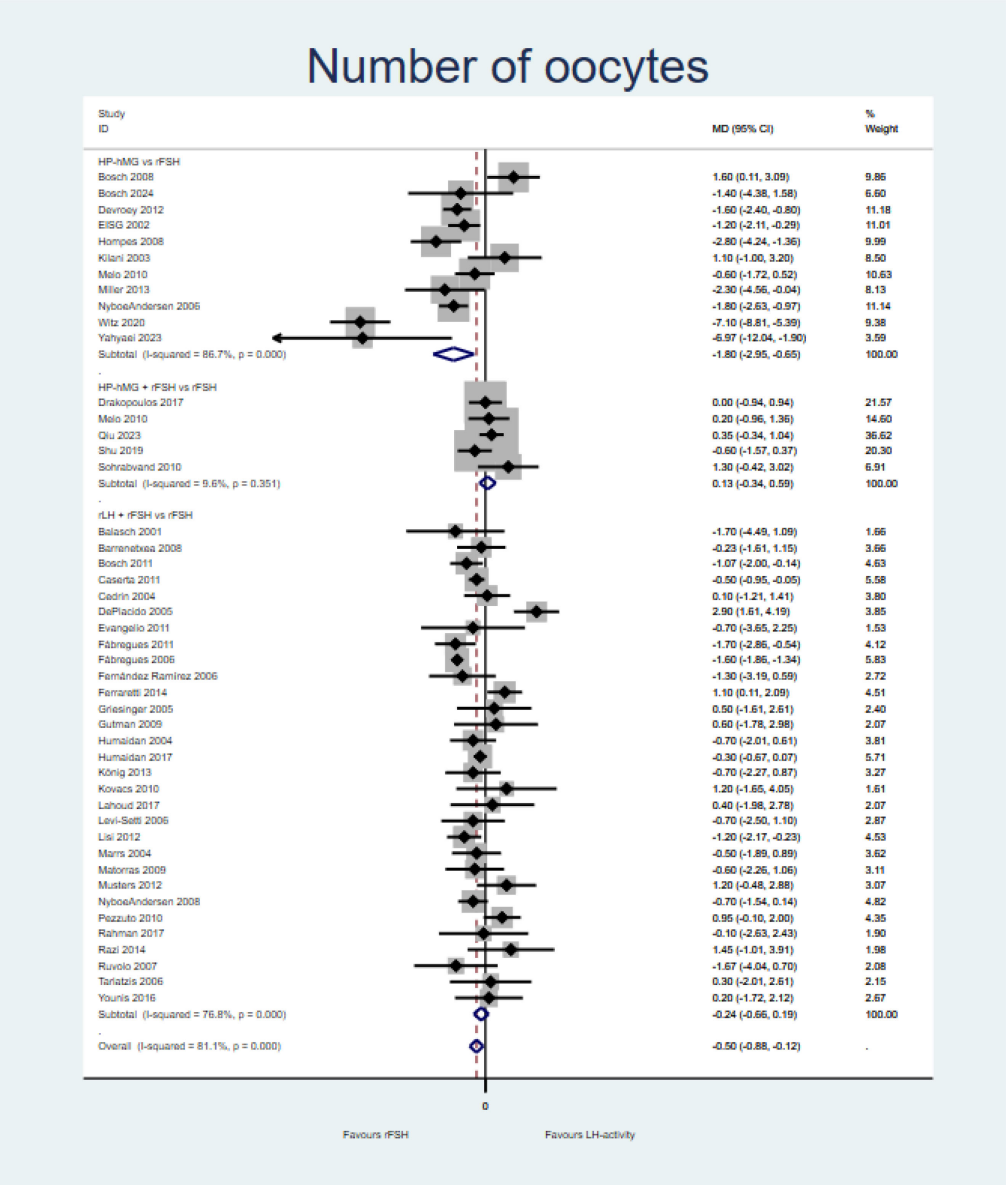
Forest plot for number of oocytes [per-protocol (PP) analysis].

Gonadotropins with LH activity (±rFSH) may result in a slight reduction in the number of oocytes retrieved in comparison with rFSH alone (MD = −0.50, 95%CI = −0.88 to −0.12, *I*^2^ = 81.1%; 46 studies, low certainty of evidence) (**Figure 4** and Summary of finding in **Supplementary Table 4**). Comparisons of HP-hMG *vs*. rFSH (RR = −1.80, 95%CI = −2.95 to 0.65; 11 studies), adding rFSH to HP-hMG *vs.* rFSH (RR = 0.13, 95%CI = −0.34 to 0.59; five studies), and adding rLH to rFSH (RR = −0.24, 95%CI = −0.88 to −0.12; 30 studies) presented similar results (**Figure 4**).

A sensitivity analysis was conducted excluding two outlier studies reporting implausible differences in the number of oocytes retrieved. The results for the HP-hMG *vs*. rFSH comparison were still heterogeneous and statistically significant, with similar confidence intervals for the pooled estimate. A second sensitivity analysis was conducted emulating an ITT analysis for each study. There were no relevant differences on this outcome overall (MD = −0.35, 95%CI = −0.74 to 0.03; 44 studies). Overall, the number of oocytes retrieved was higher in the group receiving rFSH alone compared with HP-hMG, but this was the result of two outlier studies (74, 75). The comparison of HP-hMG (with or without rFSH) *vs*. rFSH showed similar results (MD = −0.99, 95%CI = −1.81 to −0.16, *I*^2^ = 85.1%; 16 studies).

##### 3.6.1.4 Number of MII oocytes retrieved (PP analysis)

**Figure 5:**
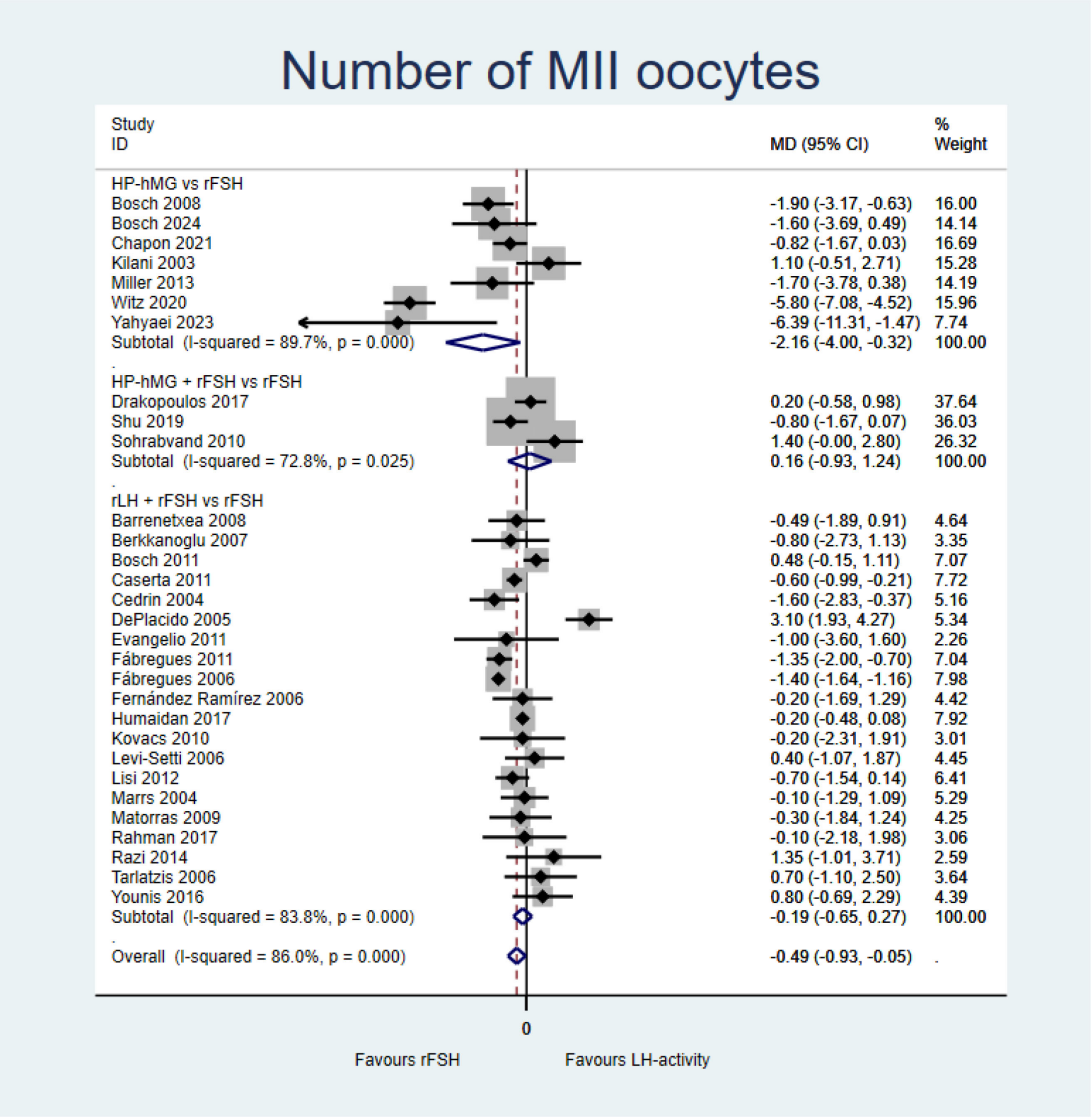
Forest plot for the number of metaphase II (MII) oocytes [per-protocol (PP) analysis].

The evidence of gonadotropins with LH activity (±rFSH) is very uncertain in the number of MII oocytes retrieved compared with rFSH alone (MD = −0.49, 95%CI = −0.93 to −0.05, *I*^2^ = 86%; 30 studies, very low certainty of evidence) (**Figure 5** and Summary of finding in **Supplementary Table 4**). Comparisons of HP-hMG *vs*. rFSH (RR = −2.16, 95%CI = −4.00 to −0.32; seven studies) presented similar results; however, adding rFSH to HP-hMG *vs.* rFSH (RR = 0.16, 95%CI = −0.93 to 1.24; three studies) and the combination of rLH and rFSH (RR = −0.19, 95%CI = −0.65 to −0.27; 20 studies) presented no differences compared with rFSH.

A *post-hoc* sensitivity analysis was conducted excluding two outlier studies that reported implausible differences in the number of oocytes retrieved (74, 75). The results were less heterogeneous and lost significance. A second sensitivity analysis was conducted emulating an ITT analysis for each study. There were no relevant differences in this outcome overall (MD = −0.23, 95%CI = −0.65 to 0.18; 29 studies). The comparison of HP-hMG (with or without rFSH) *vs*. rFSH presented similar results (MD = −0.93, 95%CI = −2.11 to 0.25, *I*^2^ = 91.3%; 10 studies).

##### 3.6.1.5 Ovarian hyperstimulation syndrome

The prevalence of OHSS ranged from 0% to 9.68% in the groups with LH activity and from 1.11% to 21.36% in the rFSH groups. In the HP-hMG arm, the prevalence ranged from 0.68% to 9.68%, while this ranged from 0% to 2.64% in the rLH arm.

**Figure 6:**
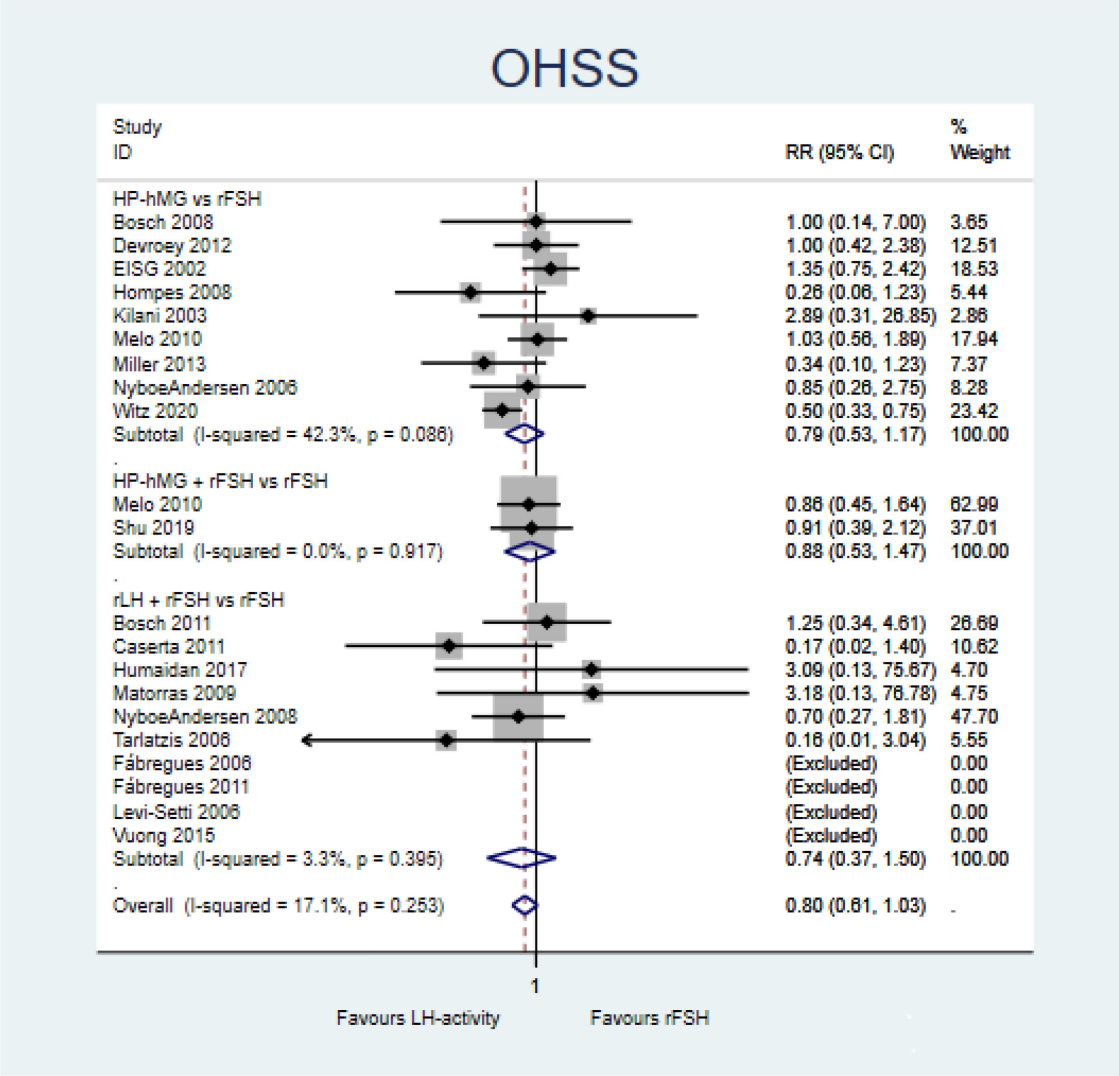
Forest plot for ovarian hyperstimulation syndrome (OHSS).

Gonadotropins with LH activity (±rFSH) probably does not increase OHSS compared with rFSH alone (RR = 0.80, 95%CI = 0.61–1.03, *I*^2^ = 17.1%; 19 studies, moderate certainty of evidence) (**Figure 6** and Summary of finding in **Supplementary Table 4**). Comparisons of HP-hMG *vs*. rFSH (RR = 0.79, 95%CI = 0.53–1.17; nine studies), adding rFSH to HP-hMG *vs.* rFSH (RR = 0.88, 95%CI = 0.53–1.47; two studies), and adding rLH to rFSH (RR = 0.74, 95%CI = 0.37–1.50; 10 studies) presented similar results (**Figure 6**).

A sensitivity analysis was conducted, defining a composite outcome that aggregated the canceled cycles due to hyperstimulation with the cases of OHSS. This outcome measures more broadly the safety of gonadotropin regimens in terms of hyperstimulation. The results were more heterogeneous and still not conclusive.

##### 3.6.1.6 Subgroup analyses of comparison 1

###### 3.6.1.6.1 By ovarian reserve

A subgroup analysis was conducted stratifying participants by ovarian reserve into three groups: poor, normal, and high. The results on live birth and pregnancies were similar across the ovarian reserve subgroups. For OHSS in the group of high reserve, HP-hMG treatment showed protective effect against OHSS (RR = 0.50, 95%CI = 0.32–0.79; two studies). However, they presented a lower number of oocytes (MD = −7.09, 95%CI = −8.71 to −5.47; two RCTs). In women with normal or high reserve and treated with HP-hMG, the number of total MII oocytes was also inferior compared with rFSH (MD = −0.93, 95%CI = −1.72 to −0.14; two RCTs; and MD = −5.84, 95%CI = −7.08 to −4.60; two RCTs) (**Supplementary Table 3**).

###### 3.6.1.6.2 By age

A subgroup analysis was conducted stratifying participants by age into two groups: participants younger and older than 35 years. The results on live birth were similar across age subgroups, although women ≤35 years seemed to have a better outcome with HP-hMG in ongoing/clinical pregnancies (RR = 1.15, 95%CI = 1.00–1.32; three RCTs). In women older than 35 years, the combination of rLH plus rFSH presented an inferior number of MII oocytes compared with rFSH alone (MD = −1.08, 95%CI = −1.50 to −0.66; six RCTs) (**Supplementary Table 3**).

###### 3.6.1.6.3 By downregulation protocol

A subgroup analysis was conducted stratifying participants by type of downregulation protocol into two groups (agonist or antagonist). The results on pregnancies were similar across protocol subgroups. However, in women treated with the GnRH agonist protocol and HP-hMG, there was an increase in the rate of the RR in live birth (1.23, 95%CI 1.0–1.51; three RCTs). In women treated with the GnRH antagonist protocol and HP-hMG, the rate of OHSS was inferior (0.54, 95%CI = 0.34–0.86; four RCTs), and the mean difference in the number of oocytes (−2.75, 95%CI = −5.27 to −0.23; six RCTs) and the mean difference in the number of MII oocytes (−2.74, 95%CI = −4.65 to −0.83; six RCTs) (**Supplementary Table 3**).

#### 3.6.2 Comparison 2: sources of LH activity

This comparison was informed by four studies (**Supplementary Table 2**): two studies comparing HP-hMG against rLH + rFSH (27, 61) and two studies comparing HP-hMG + rFSH against rLH + rFSH (42, 68).

##### 3.6.2.1 Live birth

The evidence of the combination of HP-HMG plus rFSH in comparison to rLH plus rFSH is very uncertain for live birth (RR = 1.35, 95%CI = 0.26–6.99; 175 participants, two studies, very low certainty) (Summary of finding in **Supplementary Table 5**).

##### 3.6.2.2 Ongoing pregnancy

None of the studies reported ongoing pregnancy, only clinical pregnancy. The evidence of the combination of HP-HMG plus rFSH in comparison to rLH plus rFSH may result in little to no difference in clinical pregnancy (RR = 1.05, 95%CI = 0.39–2.85; 234 participants, two studies, low certainty of evidence) (Summary of finding in **Supplementary Table 5**).

##### 3.6.2.3 Number of oocytes retrieved

The evidence of the combination of HP-HMG plus rFSH in comparison to rLH plus rFSH may result in little to no difference in the number of oocytes retrieved (MD = −1.89, 95%CI = −5.65 to 1.87; 251 participants, two studies, low certainty of evidence) (Summary of finding in **Supplementary Table 5**).

##### 3.6.2.4 Number of MII oocytes retrieved

A single study reported the number of MII oocytes retrieved (68). The evidence of the combination of HP-HMG plus rFSH in comparison to rLH plus rFSH is uncertain in the number of MII oocytes retrieved (MD = 1.33, 95%CI = −0.49 to 3.15; 140 participants, one RCT, very low certainty of evidence) (Summary of finding in **Supplementary Table 5**).

##### 3.6.2.5 Ovarian hyperstimulation syndrome

In the three studies that reported this outcome (27, 42, 68), none of the participants presented OHSS. The certainty of evidence was low.

A sensitivity analysis was conducted defining a composite outcome that aggregated the canceled cycles due to hyperstimulation with the cases of OHSS. This outcome measures more broadly the safety of gonadotropin regimens in terms of hyperstimulation. In this analysis, the fourth study (61) was included, which reported more cases of cycles canceled due to OHSS risk in the rLH + rFSH group than the HP-hMG group (7/62 and 1/60; RR = 0.16, 95%CI = 0.02–1.26), without statistical significance. The other three studies did not report any cycle canceled due to OHSS risk.

#### 3.6.3 Other comparisons

The descriptive characteristics of the studies included in other comparisons are shown in **Supplementary Table 2**. The comparison of HP-hMG + rFSH *vs*. HP-hMG was explored in two studies (56, 72). There were no differences between both groups in the probability of live births (RR = 0.80, 95%CI = 0.45–1.44; 234 participants, one study) or OHSS (RR = 0.84, 95%CI = 0.44–1.59; 570 participants, one study).

Two studies compared two different doses of rLH + rFSH (31, 38), administering a common dosage of rFSH to both study arms.

De Plácido et al. (31) compared daily rLH doses of 75 and 150 IU/day. In this study, there were more ongoing pregnancies in the higher-dosage arm, although with no statistical significance (RR = 1.21, 95%CI = 0.51–2.83; 46 participants). The number of oocytes was higher in the higher-dosage group (MD = 3.26, 95%CI = 2.18–4.34).

Fábregues et al. (38) compared daily rLH doses of 75 and 37.5 IU/day. In this study, the average number of oocytes retrieved and MII oocytes were similar across both dose groups, without statistical significance. There were more clinical pregnancies in the higher-dosage arm, although with no statistical significance (RR = 1.20, 95%CI = 0.51–2.83; 125 participants).

#### 3.6.4 Reporting biases: funnel plot

**Figure 7:**
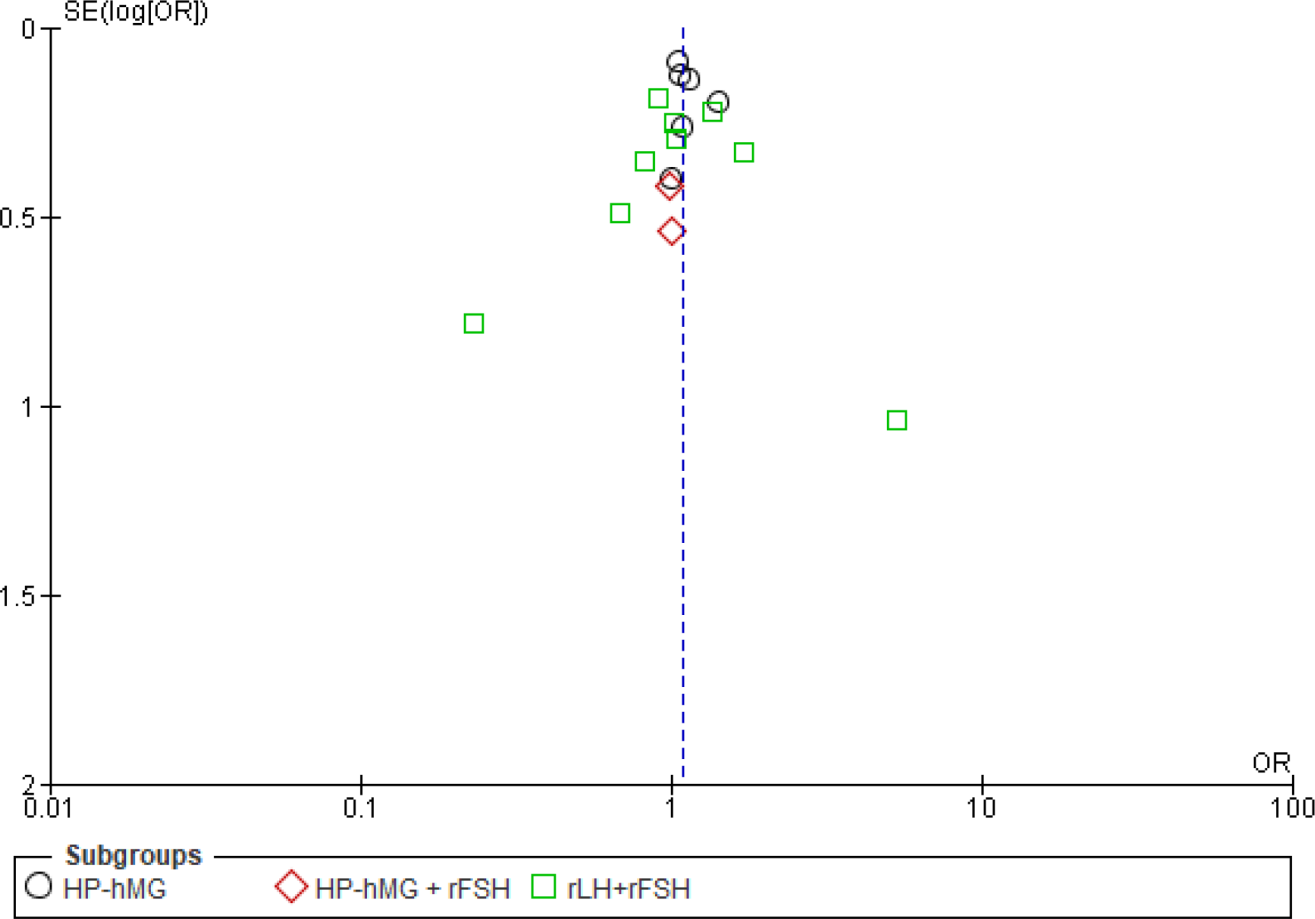
Funnel plot of gonadotropins with luteinizing hormone (LH) activity [with or without recombinant follicle-stimulating hormone (rFSH)] *versus* rFSH. Outcome: live birth.

**Figure 8:**
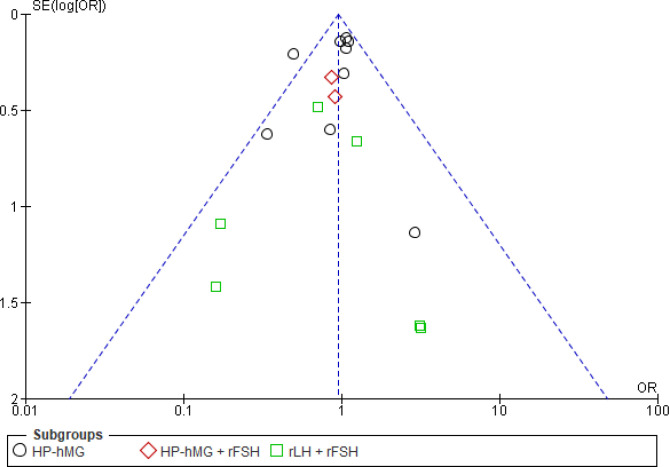
Funnel plot of gonadotropins with luteinizing hormone (LH) activity [with or without recombinant follicle-stimulating hormone (rFSH)] *versus* rFSH. Outcome: ovarian hyperstimulation syndrome (OHSS).

The funnel plot (**Figure 7**) shows an asymmetrical distribution of the studies around the measure of effect, with a lack of studies published with small effects for live birth, suggesting publication bias. However, for the outcome OHSS, there was a more homogeneous distribution of the studies, indicating no publication bias (**Figure 8**).

## 4 Discussion

This systematic review included 56 RCTs encompassing 14,034 women undergoing assisted reproductive treatments, with the exception of two studies conducted in oocyte donors. More than half of the participants underwent ovarian stimulation using GnRH agonist protocols. The most frequently evaluated comparisons were rLH plus rFSH *vs.* rFSH alone and HP-hMG *vs.* rFSH.

Overall, the evidence suggests that gonadotropins with LH activity, administered either alone or in combination with rFSH, probably result in little to no difference in the live birth or ongoing pregnancy rates when compared with rFSH alone. Their use may be associated with a slight reduction in the total number of retrieved oocytes, while the effect on the number of MII oocytes remains highly uncertain. The possible difference in the number of oocytes between gonadotropins with LH activity and rFSH was due mainly to the studies of Witz et al. (74) and Yahyaei et al. (75), which included women with high ovarian reserve. The effect of rFSH on the number of oocytes in these women was greater than gonadotropin with LH activity in women with poor or normal reserve. These results were correlated with the risk of OHSS.

Subgroup analyses did not reveal differences in the live birth rates between gonadotropins with LH activity and rFSH alone, with the notable exception of women treated with a GnRH agonist protocol receiving HP-hMG, who demonstrated higher live birth rates. In addition, women aged ≤35 years treated with HP-hMG exhibited higher ongoing and clinical pregnancy rates. In women with high ovarian reserve, HP-hMG appeared to confer a protective effect against OHSS compared with rFSH, albeit at the expense of a lower number of retrieved oocytes. Similarly, in women with normal or high ovarian reserve treated with HP-hMG, the number of MII oocytes was lower than that with rFSH. Among women older than 35 years, the combination of rLH plus rFSH was also associated with fewer MII oocytes compared with rFSH alone. In GnRH antagonist cycles, HP-hMG was associated with lower OHSS rates, as well as reduced numbers of total and MII oocytes. No other clinically meaningful differences were identified across subgroups.

When analyses were stratified by the source of LH activity, the evidence was very uncertain regarding live birth and the number of MII oocytes and suggested little to no difference in the clinical pregnancy rates or the total oocyte yield when comparing HP-hMG plus rFSH with rLH plus rFSH. Other comparisons were informed by a limited number of studies. The addition of rFSH to HP-hMG did not improve the live birth or OHSS rates compared with HP-hMG alone. Similarly, varying doses of rLH added to rFSH did not influence ongoing pregnancy rates, although higher rLH doses (150 IU/day) were associated with a greater number of retrieved oocytes compared with lower doses (75 IU/day).

The certainty of the evidence ranged from very low to moderate. Downgrading was primarily driven by RoB, substantial statistical heterogeneity, and imprecision, with the confidence intervals often encompassing no effect. Evidence certainty was moderate for live birth, ongoing pregnancy, and OHSS; low for the number of retrieved oocytes; and very low for the number of MII oocytes.

Our findings are largely consistent with previous systematic reviews. Al-Inany et al. (8) reported higher clinical pregnancy rates with HP-hMG compared with rFSH, although the differences in ongoing pregnancy and live birth were limited to the IVF subgroups. Similarly, we found no overall differences in these outcomes while identifying potential benefits of HP-hMG in women younger than 35 years and in those undergoing GnRH agonist protocols. Reviews by Santi et al. (78) and Orvieto et al. (3) reported higher oocyte yields with rFSH alone or rFSH plus rLH compared with hMG. In contrast, our results suggest that the advantage of rFSH over HP-hMG in oocyte yield is primarily confined to women with normal or high ovarian reserve and antagonist protocols.

Regarding oocyte maturity, Santi et al. (78) found no differences in MII oocyte numbers, whereas our analysis identified fewer MII oocytes with HP-hMG in women with normal or high ovarian reserve and with rLH supplementation in women of advanced maternal age. These discrepancies may reflect differences in the patient selection, COS protocols, and definitions of hypo-response across studies.

Other systematic reviews addressed related but distinct research questions. Bordewijk et al. (79) found minimal differences in the gonadotropin consumption required to achieve a live birth across rFSH, HP-hMG, and HP-FSH. Similarly, Weiss et al. (9) observed no clinically meaningful differences between urinary-derived gonadotropins and rFSH in women with clomiphene-resistant PCOS undergoing ovulation induction for intercourse or intrauterine insemination.

Recent reviews focusing specifically on rLH supplementation yielded heterogeneous results. While Conforti et al. (80, 81) reported improved clinical pregnancy rates with rLH co-treatment in hypo-responders and older women, our subgroup analyses—using low ovarian reserve and advanced age as proxies of hypo-response—did not confirm these benefits, except for a reduction in MII oocyte numbers in older women. Mao et al. (5) reported increased endometrial thickness but reduced oocyte and high-quality embryo numbers with rLH supplementation, particularly in long agonist protocols and older women. Although we did not replicate these subgroup-specific findings, our overall results similarly indicate fewer oocytes and MII oocytes with LH supplementation without improvement in the live birth or ongoing pregnancy rates.

For comparison based on the source of LH activity, there is a recent systematic review (7) focused on the comparison of rLH plus rFSH *vs*. hMG. Although Wang et al. assessed hMG, but not HP-hMG, the results on live birth and ongoing pregnancy were very similar to those in our systematic review. However, Wang et al. (7) found that the combination of rFSH and rLH produced more retrieved oocytes than hMG, while our review comparing rFSH and rLH *vs.* HP-hMG did not find differences between interventions.

A recent Cochrane network meta-analysis by Melo et al. (82) concluded that GnRH antagonist protocols reduce OHSS risk without compromising the live birth outcomes and that hMG may further reduce OHSS in predicted high responders. Our findings are consistent with these conclusions, particularly with regard to the reduced OHSS risk observed with HP-hMG in antagonist protocols and in women with high ovarian reserve.

The limitations of this review include the lack of contact with the original study authors to clarify missing or unclear data, the exclusion of seven studies due to language barriers, and the inability to retrieve two full-text articles. None of the included RCTs were specifically designed with cumulative live birth rate as a primary endpoint. Where reported, the cumulative outcomes were inconsistently defined and not suitable for pooled analysis. Furthermore, the use of 35 years for ovarian aging as a categorical threshold is largely conventional rather than biologically discrete. Ovarian aging is a continuous process. However, this threshold is historically rooted in research convention and regulatory frameworks rather than in precise endocrine inflection points. Emerging real-world data suggest that more clinically meaningful transitions may occur around ages 32 and 38 years, reflecting gradual changes in follicular competence and chromosomal integrity. However, as a systematic review, we were constrained by the predefined categorizations used in the original RCTs.

Nevertheless, this review has several strengths. It followed a prespecified, registered protocol (12), employed a comprehensive search strategy, and focused exclusively on highly purified hMG preparations to ensure methodological rigor. Broad eligibility criteria allowed the inclusion of diverse patient populations, and the outcomes encompassed clinically relevant, efficacy, and safety endpoints. Rigorous Cochrane methods were applied, including a random effects model, subgroup and sensitivity analyses, and ITT analyses.

## 5 Conclusion

This systematic review provides a comprehensive evaluation of the efficacy and safety of gonadotropin formulations with and without LH activity in COS. Overall, gonadotropins with LH activity probably result in little to no difference in the live birth or ongoing pregnancy rates compared with rFSH alone, despite a modest reduction in oocyte yield. Importantly, their use does not appear to increase the risk of OHSS and may confer safety advantages in specific subgroups, such as in women with high ovarian reserve and in those undergoing GnRH antagonist protocols.

Analyses stratified by age, ovarian reserve, and downregulation protocol highlight the need for individualized treatment strategies. Future RCTs should focus on clearly defined patient subgroups, standardize the definitions of hypo-response and ovarian reserve, and clarify the role of LH activity in optimizing both the efficacy and safety outcomes in assisted reproduction.
