# Supplementary material for: Luteinizing hormone activity in ovarian stimulation: comparative efficacy and safety of gonadotropins versus recombinant follicle-stimulating hormone—a systematic review and meta-analysis
Source: Front Endocrinol (Lausanne). 2026 Apr 22;17:1792900. doi: 10.3389/fendo.2026.1792900 (PMC13143684; doi:10.3389/fendo.2026.1792900)
Supplement: Supplementary file 1 [file DataSheet1.docx]

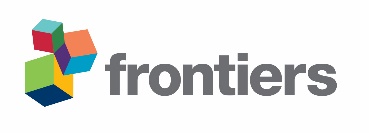


Supplementary Material

**Search strategy for MEDLINE**

PubMed

#1 "highly purified human menotropin*"[tiab]

#2 "HP-hMG"[tiab]

#3 "HPhMG"[tiab]

#4 "HP-hMG"[tiab]

#5 "hMGHP"[tiab]

#6 hMG[tiab]

#7 Menopur[tiab]

#8 Meropur[tiab]

#9 Meriofert[tiab]

#10 Menogon[tiab]

#11 "HMG rFSH"[tiab:~3]

#12 "human menopausal gonadotropin*"[tiab]

#13 "human menopausal gonadotrophin*"[tiab]

#14 uHMG[tiab]

#15 "rFSH rLH"[tiab:~3]

#16 "recombinant FSH"[tiab] AND "recombinant LH"[tiab]

#17 "recombinant follicle stimulating hormone*"[tiab] AND "recombinant LH"[tiab] 12

#18 Pergoveris[tiab]

#19 #1 OR #2 OR #3 OR #4 OR #5 OR #6 OR #7 OR #8 OR #9 OR #10 OR #11 OR #12 OR #13 OR #14 OR #15 OR #16 OR #17 OR #18

#20 "Ovulation Induction"[Mesh]

#21 "ovarian stimulation"[tiab]

#22 "ovulation induction"[tiab]

#23 COS[tiab]

#24 "embryo transfer*"[tiab]

#25 "assisted reproduct*"[tiab]

#26 ART[tiab]

#27 IVF[tiab]

#28 ICSI[tiab]

#29 fertili*[tiab]

#30 infertil*[tiab]

#31 #20 OR #21 OR #22 OR #23 OR #24 OR #25 OR #26 OR #27 OR #28 OR #29 OR #30

#32 #19 AND #31

#33 (randomized controlled trial[pt] OR controlled clinical trial[pt] OR randomized[tiab] OR placebo[tiab] OR drug therapy[sh] OR randomly[tiab] OR trial[tiab] OR groups[tiab]) NOT (animals [mh] NOT humans [mh])

#34 #32 AND #33

#35 #32 NOT #34

#36 "Cohort Studies"[Mesh]

#37 "Comparative Study"[pt]

#38 cohort*[tiab]

#39 outcome*[ti]

#40 observational[ti]

#41 efficacy[tiab]

#42 effectiv*[tiab]

#43 comparative[tiab]

#44 prospectiv*[tiab]

#45 controlled[tiab]

#46 propensity[tiab]

#47 chart[tiab]

#48 claim[tiab] OR claims[tiab]

#49 regist*[tiab]

#50 "real life"[tiab]

#51 "real world"[tiab]

#52 #36 OR #37 OR #38 OR #39 OR #40 OR #41 OR #42 OR #43 OR #44 OR #45 OR #46 OR #47 OR #48 OR #49 OR #50 OR #51

#53 #35 AND #52 289

**Search strategy for EMBASE**

Embase.com

#1 'highly purified human menotropin':ti,ab

#2 'hp-hmg':ti,ab

#3 'hphmg':ti,ab

#4 'HP-hMG':ti,ab

#5 'hmghp':ti,ab

#6 'hmg':ti,ab

#7 menopur:ti,ab

#8 meropur:ti,ab

#9 meriofert:ti,ab

#10 menogon:ti,ab

#11 ((hmg NEAR/5 rfsh):ti,ab)

#12 'human menopausal gonadotropin*':ti,ab

#13 'human menopausal gonadotrophin*':ti,ab

#14 uhmg:ti,ab

#15 ((rfsh NEAR/5 rlh):ti,ab)

#16 (('recombinant fsh' NEAR/5 'recombinant lh'):ti,ab)

#17 (('recombinant follicle stimulating hormone*' NEAR/5 'recombinant lh'):ti,ab)

#18 pergoveris:ti,ab

#19 #1 OR #2 OR #3 OR #4 OR #5 OR #6 OR #7 OR #8 OR #9 OR #10 OR #11 OR #12 OR #13 OR #14 OR #15 OR #16 OR #17 OR #18

#20 'ovulation induction'/de

#21 'ovarian stimulation':ti,ab

#22 'ovarian induction':ti,ab

#23 cos:ti,ab

#24 'embryo transfer*':ti,ab

#25 'assisted reproduct*':ti,ab

#26 art:ti,ab

#27 ivf:ti,ab

#28 icsi:ti,ab

#29 fertili*:ti,ab

#30 infertili*:ti,ab

#31 #20 OR #21 OR #22 OR #23 OR #24 OR #25 OR #26 OR #27 OR #28 OR #29 OR #30

#32 #19 AND #31

#33 'randomized controlled trial'/de

#34 'controlled clinical trial'/de

#35 random*:ti,ab,tt

#36 'randomization'/de

#37 'intermethod comparison'/de

#38 placebo:ti,ab,tt

#39 (compare:ti,tt OR compared:ti,tt OR comparison:ti,tt)

#40 (evaluated:ab OR evaluate:ab OR evaluating:ab OR assessed:ab OR assess:ab) AND (compare:ab OR compared:ab OR comparing:ab OR comparison:ab)

#41 ((open NEXT/1 label):ti,ab,tt)

#42 (((double OR single OR doubly OR singly) NEXT/1 (blind OR blinded OR blindly)):ti,ab,tt)

#43 'double blind procedure'/de

#44 ((parallel NEXT/1 group*):ti,ab,tt)

#45 (crossover:ti,ab,tt OR 'cross over':ti,ab,tt)

#46 (((assign* OR match OR matched OR allocation) NEAR/6 (alternate OR group OR groups OR intervention OR interventions OR patient OR patients OR subject OR subjects OR participant OR participants)):ti,ab,tt)

#47 (assigned:ti,ab,tt OR allocated:ti,ab,tt)

#48 ((controlled NEAR/8 (study OR design OR trial)):ti,ab,tt)

#49 (volunteer:ti,ab,tt OR volunteers:ti,ab,tt)

#50 'human experiment'/de

#51 trial:ti,tt

#52 #33 OR #34 OR #35 OR #36 OR #37 OR #38 OR #39 OR #40 OR #41 OR #42 OR #43 OR #44 OR #45 OR #46 OR #47 OR #48 OR #49 OR #50 OR #51

#53 ((random* NEXT/1 sampl* NEAR/8 ('cross section*' OR questionnaire* OR survey OR surveys OR database OR databases)):ti,ab,tt) NOT ('comparative study'/de OR 'controlled study'/de OR 'randomised controlled':ti,ab,tt OR 'randomized controlled':ti,ab,tt OR 'randomly assigned':ti,ab,tt)

#54 'cross-sectional study' NOT ('randomized controlled trial'/de OR 'controlled clinical study'/de OR 'controlled study'/de OR 'randomised controlled':ti,ab,tt OR 'randomized controlled':ti,ab,tt OR 'control group':ti,ab,tt OR 'control groups':ti,ab,tt)

#55 'case control*':ti,ab,tt AND random*:ti,ab,tt NOT ('randomised controlled':ti,ab,tt OR 'randomized controlled':ti,ab,tt)

#56 'systematic review':ti,tt NOT (trial:ti,tt OR study:ti,tt)

#57 nonrandom*:ti,ab,tt NOT random*:ti,ab,tt

#58 'random field*':ti,ab,tt

#59 (('random cluster' NEAR/4 sampl*):ti,ab,tt)

#60 review:ab AND review:it NOT trial:ti,tt

#61 'we searched':ab AND (review:ti,tt OR review:it)

#62 'update review':ab

#63 ((databases NEAR/5 searched):ab)

#64 (rat:ti,tt OR rats:ti,tt OR mouse:ti,tt OR mice:ti,tt OR swine:ti,tt OR porcine:ti,tt OR murine:ti,tt OR sheep:ti,tt OR lambs:ti,tt OR pigs:ti,tt OR piglets:ti,tt OR rabbit:ti,tt OR rabbits:ti,tt OR cat:ti,tt OR cats:ti,tt OR dog:ti,tt OR dogs:ti,tt OR cattle:ti,tt OR bovine:ti,tt OR monkey:ti,tt OR monkeys:ti,tt OR trout:ti,tt OR marmoset*:ti,tt) AND 'animal experiment'/de

#65 'animal experiment'/de NOT ('human experiment'/de OR 'human'/de)

#66 #53 OR #54 OR #55 OR #56 OR #57 OR #58 OR #59 OR #60 OR #61 OR #62 OR #63 OR #64 OR #65

#67 #52 NOT #66

#68 #32 AND #67

#69 #32 AND #67 AND ([conference abstract]/lim OR [conference paper]/lim OR [conference review]/lim)

#70 #68 NOT #69

#71 #32 NOT #68

#72 'cohort analysis'/de

#73 'comparative study'/exp

#74 cohort*:ti,ab

#75 outcome*:ti

#76 observational:ti

#77 efficacy:ti,ab

#78 effectiv*:ti,ab

#79 comparative:ti,ab

#80 prospectiv*:ti,ab

#81 controlled:ti,ab

#82 propensity:ti,ab

#83 chart:ti,ab

#84 claim:ti,ab

#85 claims:ti,ab

#86 regist*:ti,ab

#87 'real life':ti,ab

#88 'real world':ti,ab

#89 #72 OR #73 OR #74 OR #75 OR #76 OR #77 OR #78 OR #79 OR #80 OR #81 OR #82 OR #83 OR #84 OR #85 OR #86 OR #87 OR #88

#90 #71 AND #89

#91 #71 AND #89 AND ([conference abstract]/lim OR [conference paper]/lim OR [conference review]/lim)

#92 #90 NOT #91

#93 #70 OR #92

**Search strategy for Cochrane Central Register of Controlled Trials**

The Cochrane Library

Issue 10 of 12, February 2025

#1 'highly purified human menotropin':ti,ab

#2 "HP-hMG":ti,ab

#3 "HPhMG":ti,ab

#4 "HP-hMG":ti,ab

#5 "hMGHP":ti,ab

#6 hMG:ti,ab

#7 Menopur:ti,ab

#8 Meropur:ti,ab

#9 Meriofert:ti,ab

#10 Menogon:ti,ab

#11 (HMG NEAR/5 rFSH):ti,ab

#12 (human NEXT menopausal NEXT gonadotropin*):ti,ab

#13 (human NEXT menopausal NEXT gonadotrophin*):ti,ab

#14 uHMG:ti,ab

#15 (rFSH NEAR/5 rLH):ti,ab

#16 ("recombinant FSH" NEAR/5 "recombinant LH"):ti,ab

#17 (recombinant NEXT follicle NEXT stimulating NEXT hormone*):ti,ab NEAR/5 "recombinant LH":ti,ab

#18 Pergoveris:ti,ab

#19 #1 OR #2 OR #3 OR #4 OR #5 OR #6 OR #7 OR #8 OR #9 OR #10 OR #11 OR #12 OR #13 OR #14 OR #15 OR #16 OR #17 OR #18

#20 MeSH descriptor: [Ovulation Induction] explode all trees

#21 "ovarian stimulation":ti,ab

#22 "ovulation induction":ti,ab

#23 COS:ti,ab

#24 (embryo NEXT transfer*):ti,ab

#25 (assisted NEXT reproduct*):ti,ab

#26 ART:ti,ab

#27 IVF:ti,ab

#28 ICSI:ti,ab

#29 fertili*:ti,ab

#30 infertil*:ti,ab

#31 #20 OR #21 OR #22 OR #23 OR #24 OR #25 OR #26 OR #27 OR #28 OR #29 OR #30

#32 #19 AND #31

### Supplementary Table 1 References of excluded studies and reasons to be excluded

|  | **Cause of exclusion** |
| --- | --- |
| Aboulghar M A, Mansour R T, Serour G I, Amin Y M, Sattar M A, ElAttar E. Recombinant follicle-stimulating hormone in the treatment of patients with history of severe ovarian hyperstimulation syndrome. Fertility and Sterility. 1996;66(5):757-60. | Wrong comparator: HP-hMG was not assessed |
| Aboulghar M A, Mansour R T, Serour G I, Amin Y M, Sattar M A, Elattar E. Recombinant follicle-stimulating hormone in the treatment of patients with history of severe ovarian hyperstimulation syndrome. Fertility and Sterility. 1998;69(3 SUPPL. 2):72S-5S. | Wrong comparator: HP-hMG was not assessed |
| ACTRN12611001133921. A randomized trial to assess the importance of Human Menopausal Gonadotropin co-stimulation in in vitro fertilization programs. 2011. | Trial register |
| ACTRN12612000731897. Different treatment modalities for women with polycystic ovary like phenotype undergoing assisted reproduction. 2012. | Trial register |
| ACTRN12612000294853. A trial to compare highly purified human menopausal gonadotropins (hMG) and recombinant follicular stimulating hormone (rFSH) to improve pregnancy rates in infertile women. 2012. | Trial register |
| Agrawal R, Conway G S, Engmann L, Bekir J S, Jacobs H S. Implications of using follicle-stimulating hormone preparations depleted of luteinizing hormone to achieve follicular growth in vitro fertilization. Gynecol Endocrinol 1998;12(1):9-15. | Wrong comparator: HP-hMG and recombinant Gonadotropins were not assessed |
| Agustina A M, Gisela J, Constanza F, Eugenia I, Fernanda U, Ines C, et al. Controlled ovarian stimulation affects the immunological quality of the ovary and endometrium. American Journal of Reproductive Immunology. 2013;69:167‐168. | Abstract in meeting |
| Alama Faubel P, Cruz M, Munoz M, Pacheco A, Collado D, Requena A. Type of gonadotropin does not affect either oocyte quality or follicular fluid endocrine profile during ovarian stimulation in oocyte donors. Human reproduction (Oxford). 2016;31:i293-. | Abstract in meeting |
| Almog B, Azem F, Kapustiansky R, Azolai J, Wagman I, Levin I, et al. Intrafollicular and serum levels of leptin during in vitro fertilization cycles: comparison between the effects of recombinant follicle-stimulating hormones and human menopausal gonadotrophin. Gynecological Endocrinology. 2011;27(9):666‐668-. | Wrong comparator: Not HP-hMG |
| Alsbjerg B, Povlsen B B, Laursen R J, Elbeak H O, Andersen C Y, Humaidan P. Endocrine effects of rhCG or rLH supplementation to rFSH stimulation in a GnRHa long down-regulation protocol-a randomized controlled study. Human reproduction (Oxford). 2014;29:i50-. | Abstract in meeting |
| Alsbjerg B, Elbaek H O, Laursen R J, Povlsen B B, Haahr T, Yding Andersen C, et al. Bio-equivalent doses of recombinant HCG and recombinant LH during ovarian stimulation result in similar oestradiol output: a randomized controlled study. Reprod Biomed Online. 2017;35(2):232-8. | Wrong comparator: Not HP-hMG |
| Ana M, Vicente M, María R J, Trinidad G G, Alberto R. Observational study to assess the therapeutic value of four ovarian hyperstimulation protocols in IVF after pituitary suppression with GnRH antagonists in normally responding women. Clin Med Insights Reprod Health. 2011;5:1-9. | Not RCT |
| Anderson S, Norris H, Hartlein T, Davies E B, Brasile D, Gocial B, et al. A prospective randomized trial to compare recombinant follicle stimulating hormone (rFSH) versus highly purified human menotropin (HP-hMG) for controlled ovarian stimulation on blastocyst aneuploidy rates. Fertility and Sterility. 2018;110(4 suppl):e201-. | Abstract in meeting |
| Antoine J M, Salat-Baroux J, Alvarez S, Cornet D, Tibi C, Brieu V, et al. Preliminary data on the efficacy of a new human menopausal gonadotropin (hMG) preparation containing a reduced amount of luteinizing hormone (LH) for superovulation in an in vitro fertilization (IVF) program. Journal of Assisted Reproduction and Genetics. 1992;9(4):404-6. | Wrong comparator: Not HP-hMG |
| Arce J C, Smitz J. Exogenous hCG activity, but not endogenous LH activity, is positively associated with live birth rates in anovulatory infertility. Human fertility (Cambridge). 2011;14(3):192‐199 | Wrong outcomes |
| Ashmita J, Vikas S, Swati G. The impact of progesterone level on day of hCG injection in IVF Cycles on clinical pregnancy rate. Journal of Human Reproductive Sciences. 2017;10(4):265-70. | Wrong comparator: Not HP-hMG |
| Asimakopoulos B, Al-Hasani S, Nikolettos N, Diedrich K. A comparison of the ovarian response to external stimulation between women with right or left ovary participating in programs of ICSI/ET. Archives of Gynecology and Obstetrics. 2003;268(3):168-71. | Wrong study design |
| Assou S, Moussaddykine S, Van Den Abbeel E, Arce J C. Human cumulus cell biomarkers for predicting top blastocyst development and pregnancy in single blastocyst transfer. Fertility and Sterility. 2011;96(3):S16-. | Wrong comparator: it was a genetic study |
| Aydin Y, Cepni I, Ocal P, Aydin B, Aydogan B, Salahov R, et al. A randomised open-label trial comparing highly purified hMG and recombinant FSH in a GnRH agonist cycle. Human reproduction (Oxford). 2012;27. | Abstract in meeting |
| Bagratee J S, Lockwood G, López Bernal A, Barlow D H, Ledger W L. Comparison of highly purified FSH (Metrodin-high purity) with pergonal for IVF superovulation. Journal of Assisted Reproduction and Genetics. 1998;15(2):65-9. | Wrong comparator: it was assessed HP-FSH and not rFSH. |
| Balasch J, Fábregues F, Creus M, Moreno V, Puerto B, Peñarrubia J, et al. Pure and highly purified follicle-stimulating hormone alone or in combination with human menopausal gonadotrophin for ovarian stimulation after pituitary suppression in in-vitro fertilization. Human Reproduction. 1996;11(11):2400-4. | Wrong comparator: Not HP-hMG neither rFSH |
| Balasch J, Fábregues F, Casamitjana R, Peñarrubia J, Vanrell J A. A pharmacokinetic and endocrine comparison of recombinant follicle-stimulating hormone and human menopausal gonadotrophin in polycystic ovary syndrome. Reproductive BioMedicine Online. 2003;6(3):296-301. | Wrong comparator: Not HP-hMG |
| Barros Delgadillo J, Ruiz Valderrama C, Rios Barba M, De La Jara Diaz J. Should women delay pregnancy following laparoscopic adjustable gastric banding. Abstract withdrawn by the author P-524 Comparison of results of two flexible ovarian hyperstimulation protocols with two different initial doses (150 VS = 225 UI) in patients undergoing FIV/ICSI cycles. Human reproduction (Oxford). 2014;29:i334-. | Wrong comparator: Not HP-hMG |
| Bassil S, Wyns C, Donnez J. A randomized prospective cross-over study of highly purified follicle-stimulating hormone and human menopausal gonadotrophin for ovarian hyperstimulation in women aged 37-41 years. Journal of Assisted Reproduction and Genetics. 2000;17(2):107‐112-. | Wrong comparator: Not HP-hMG neither rFSH |
| Batiza V, Sants R, Galache P, Hernandez D, Montoya M, Ruy Sanchez M, et al. Comparative study of recombinant follicle stimulating hormone (recFSH) and highly purified FSH (hp FSH) plus Menotrophins (hMG) in intracytoplasmic sperm injection (ICSI) program. Fertility and Sterility. 1999;72(3 Suppl 1):S109‐110-. | Wrong comparator: Not HP-hMG |
| Bauman R, Vujisic S, Tripalo A, Aksamija A, Hafner D, Emedi I, et al. Influence of hormonal stimulation on in vitro fertilization/embryo transfer outcome. European Journal of Obstetrics and Gynecology and Reproductive Biology. 2005;119(1):94-102. | Wrong comparator: Not HP-hMG |
| Behre H M, Howles C M, Longobardi S. Luteinizing hormone supplementation from Day 1 versus 6 of ovarian stimulation in women aged 36n40 years: results from an open-label, randomized, multicentre, multinational trial. Human reproduction (Oxford). 2013;28:i239-. | Abstract in meeting |
| Bejarano Velazquez D, De La O Perez L O, Trevino Baez J D, Gonzalez Diaz O A. A randomized trial comparing the efficacy and safety of rFSH+rLH vs. rFSH alone and hMG to induce ovulation in cycles for low complexity techniques. Human reproduction (Oxford). 2016;31:i304-. | Abstract in meeting |
| Berkkanoglu M, Isikoglu M, Ozgur K. Clinical effects of ovulation induction with recombinant FSH (rFSH) supplemented with recombinant LH (rLH) or low-dose recombinant HCG (rHCG) in the midfollicular phase in microdose cycles. Fertility and Sterility. 2005;84(Suppl 1):S44‐45-. | Abstract in meeting |
| Bilge M, Ozdemirci S, Esinler D, Karahanoglu E, Esinler I, Aksu T. Assessment of follicular and serum VEGF and IGF-1 in ICSI patients: hMG vs rFSH. Clinical and Experimental Obstetrics and Gynecology. 2015;42(5):576-9. | Wrong comparator: Not HP-hMG |
| Bing H, Cheng J, Huang L, Tan W, Xue L, Wang S. Effects of human menopausal gonadotropin on zona pellucida and pregnancy outcomes of ovarian stimulation protocols. Iranian Journal of Reproductive Medicine. 2015;13(6):337-44. | Wrong comparator: Not HP-hMG |
| Bissonnette F, Minano Masip J, Kadoch I J, Librach C, Sampalis J, Yuzpe A. Individualized ovarian stimulation for in vitro fertilization: a multicenter, open label, exploratory study with a mixed protocol of follitropin delta and highly purified human menopausal gonadotropin. Fertility and Sterility. 2021;115(4):991-1000. | Not RCT |
| Bjercke S, Tanbo T, Åbyholm T, Omland A, Opøien H K, Fedorcsak P. Clinical outcome following stimulation with highly purified hMG or recombinant FSH in patients undergoing their first treatment cycle of IVF or ICSI. Acta Obstetricia et Gynecologica Scandinavica.  2010;89(8):1053-60. | Not RCT |
| Bleau N, Agdi M, Son W, Tan S, Dahan M H. A Comparison of outcomes from in vitro fertilization cycles stimulated with follicle stimulating hormone plus either recombinant luteinizing hormone or human menopausal gonadotropins in subjects treated with long gonadotropin releasing hormone agonist protocols. Int J Fertil Steril. 2017;11(2):79-84. | Wrong comparator: Not HP-hMG |
| Bordewijk E M, Mol F, Van Der Veen F, Van Wely M. Required amount of rFSH, HP-hMG and HP-FSH to reach a live birth: A systematic review and meta-analysis. Human Reproduction Open.2019;2019(3). | Wrong study design |
| Bosch E, Labarta E, Vidal C, Giles J, Bellver J, Zuzuarregui J L, et al. The relationship between serum androgen levels and the need of LH administration during controlled ovarian stimulation for in vitro fertilization: an explorative study. Human reproduction (Oxford). 2011;26:i26-. | Abstract in meeting |
| Bosch Aparicio E, Alama P, Romero J L, Mari M, Labarta E. Follicular steroidogenesis in GnRH antagonist ovarian stimulation cycles with r-FSH vs. hp-HMG. Human reproduction (Oxford). 2019. 34 SUPPL 1 | Abstract in meeting |
| Brannian J, Eyster K, Mueller B A, Bietz M G, Hansen K. Differential gene expression in human granulosa cells from recombinant FSH versus human menopausal gonadotropin ovarian stimulation protocols. Reproductive Biology and Endocrinology. 2010;8. | Wrong patient population |
| Bühler K F, Fischer R, Verpillat P, Allignol A, Guedes S, Boutmy E, et al. Comparative effectiveness of recombinant human follicle-stimulating hormone alfa (r-hFSH-alfa) versus highly purified urinary human menopausal gonadotropin (hMG HP) in assisted reproductive technology (ART) treatments: a non-interventional study in Germany. Reproductive Biology and Endocrinology. 2021;19(1). | Not RCT |
| Bühler K, Roeder C, Schwarze J E, Lispi M, Allignol A, Falla E, et al. Cost-effectiveness analysis of recombinant human follicle-stimulating hormone alfa(r-hFSH) and urinary highly purified menopausal gonadotropin (hMG) based on data from a large German registry. Best Practice and Research: Clinical Obstetrics and Gynaecology. 2022;85:188-202. | Wrong outcomes |
| Burgués S. The effectiveness and safety of recombinant human LH to support follicular development induced by recombinant human FSH in WHO group I anovulation: evidence from a multicentre study in Spain. Hum Reprod. 2001;16(12):2525-32. | Wrong study design |
| Campo S, Garcea N. Efficacy assessment of highly purified follicle-stimulating hormone alone or in combination with human menopausal gonadotropin during pituitary suppression in patients undergoing GIFT for unexplained infertility. Gynecological Endocrinology. 1998;12(3):161-6. | Wrong comparator: Not HP-hMG |
| Canosa S, Carosso A R, Mercaldo N, Ruffa A, Evangelista F, Bongioanni F, et al. Effect of rLH Supplementation during Controlled Ovarian Stimulation for IVF: Evidence from a Retrospective Analysis of 1470 Poor/Suboptimal/Normal Responders Receiving Either rFSH plus rLH or rFSH Alone. Journal of Clinical Medicine. 2022;11(6). | Not RCT |
| Carone D, Vizziello G, Vitti A, Chiappetta R. Clinical outcomes of ovulation induction in WHO group i anovulatory women using r-hFSH + r-hLH in a 2: 1 ratio compared to hMG. 2 Human reproduction (Oxford). 010;25 suppl 1(6):i312-. | Abstract in meeting |
| Çelik C, Sofuoǧlu K, Selçuk S, Asoǧlu M R, Abali R, Çetingöz E, et al. Comparison of ovulation induction and pregnacy outcomes in IVF patients with normal ovarian reserve who underwent long protocol with recombinant-FSH and highly purified-hMG. Journal of the Turkish German Gynecology. 2011;12(1):15-20. | Not RCT |
| Cerrillo Martinez M, Cruz Palomino M, Ferrando R, Garcia Velasco J A. Freeze-all strategy in endometriosis patients-a new indication? Human reproduction (Oxford). 2017;32:i253-. | Abstract in meeting |
| Chang J C, Yi Y C, Chen Y F, Guu H F, Kung H F, Chen L Y, et al. A direct healthcare cost analysis of recombinant LH versus hMG supplementation on FSH during controlled ovarian hyperstimulation in the GnRH-antagonist protocol. Archives of Gynecology and Obstetrics. 2024;309(2):699-706. | Wrong outcomes |
| Chawla M, Fakih M, Devroey P, Qasim O, Shunnar A, Emerson G, et al. Outcomes of in vitro fertilization in poor responders of ≤ 40 years of age using either daily gonadotropin stimulation or long acting FSH stimulation. Human reproduction (Oxford). 2017;32:i467-. | Abstract in meeting |
| Check J H, O'Shaughnessy A, Nazari A, Hoover L. Comparison of efficacy of high-dose pure follicle-stimulating hormone versus human menopausal gonadotropins for in vitro fertilization. Gynecologic and Obstetric Investigation. 1995;40(2):117-9. | Wrong comparator: Not HP-hMG neither rFSH |
| Check M, Wilson C, Check J H, Kiefer D, Choe J K. Evidence that exclusive use of Follistim® may produce better pregnancy results than the use of Gonal-F® following in vitro fertilization (IVF) - Embryo transfer (ET). Clinical and Experimental Obstetrics and Gynecology. 2002;29(3):183-4. | Wrong outcomes |
| Chedid S, Agiman R, Ikeda F, Matsuzaki C, Nunes C, Grieco A. Evaluation of the efficacy of adding recombinant LH hormone to recombinant FSH hormone in cycles of non-selected patients submitted to controlled ovarian stimulation for assisted reproduction. The 21st annual meeting of the european society of human reproduction and embryology. 2005;i122-. | Abstract in meeting |
| Chen L H, Chin T H, Huang S Y, Yu H T, Chang C L, Huang H Y, et al. Supplementation with human menopausal gonadotropin in the gonadotropin-releasing hormone antagonist cycles of women with high AMH: Pregnancy outcomes and serial hormone levels. Taiwanese Journal of Obstetrics and Gynecology. 2021;60(4):739-44. | Not RCT |
| Chen M J, Yi Y C, Guu H F, Chen Y F, Kung H F, Chang J C, et al. A retrospective, matched case-control study of recombinant LH versus hMG supplementation on FSH during controlled ovarian hyperstimulation in the GnRH-antagonist protocol. Frontiers in Endocrinology. 2022;13. | Wrong study design |
| Cheung A P, Pride S M, Yuen B H, Sy L. In-vivo ovarian androgen responses to recombinant FSH with and without recombinant LH in polycystic ovarian syndrome. Human Reproduction. 2002;17(10): 2540-7. | Wrong outcomes |
| ChiCTR-IPR-14005678. A comparison of outcomes from ART cycles stimulated with recombinant follicle stimulating hormone and highly purified human menopausal gonadotropin. 2014. | Trial register |
| ChiCTR-TRC-14004552. A multicenter, prospective, randomized control study to compare the impact of high purity hMG (HP-HMG) plus recombinant human follicle-stimulating hormone (rFSH) versus mono use of recombinant human follicle-stimulating hormone (rFSH) on HCG day’s progesterone level based on GnRHa long protocol. 2014. | Trial register |
| ChiCTR-TRC-14004965. A prospective, randomized, controlled trial of recombinant FSH combined with HP-HMG on ovarian response and clinical outcomes. 2014. | Trial register |
| ChiCTR-OPR-15006204. Efficacy of highly purified HMG vs. HMG on pregnancy outcomes in vitro fertilization. 2015. https://trialsearch.who.int/Trial2.aspx?TrialID=ChiCTR-OPR-15006204 | Wrong comparator: HP-hMG vs. hMG with rFSH |
| ChiCTR-IPR-16008329. Comparison of Different Gonadotropins in Non-PCOS Patients with High Ovarian Reserve undergoing in vitro Fertilization: a Randomized Controlled Trial. 2016. | Trial register |
| ChiCTR-IPR-16008355. Comparison of Different Gonadotropins in PCOS Patients undergoing in vitro Fertilization: a Randomized Controlled Trial. 2016. | Trial register |
| Choi D, Hwang S S, Lee E Y, Park C E, Yoon B K, Lee J H, et al. Recombinant FSH and pregnancy-associated plasma protein. 2003;109(2):171-6. | Wrong comparator: uFSH + hMG (Pergonal) vs rFSH vs uFSH |
| Commenges-Ducos M, Piault S, Papaxanthos A, Ribes C, Dallay D, Commenges D. Recombinant follicle-stimulating hormone versus human menopausal gonadotropin in the late follicular phase during ovarian hyperstimulation for in vitro fertilization. Fertility and Sterility. 2002;78(5):1049-54. | Wrong comparator: Not HP-hMG |
| Conforti A, Esteves S C, Di Rella F, Strina I, De Rosa P, Fiorenza A, et al. The role of recombinant LH in women with hypo-response to controlled ovarian stimulation: a systematic review and meta-analysis. Reprod Biol Endocrinol. 2019;17(1):18-. | Wrong study design: Systematic review |
| Cruz M, Requena A, Agudo D, García-Velasco J A. Type of gonadotropin used during controlled ovarian stimulation induces differential gene expression in human cumulus cells: A randomized study. European Journal of Obstetrics and Gynecology and Reproductive Biology. 2017;215:124-33. | Wrong comparator: Not HP-hMG |
| Cruz M, Ruiz M E. HP-hMG in monotherapy improves clinical outcomes in young women. Fertility and Sterility. 2020;114(3):e554-. | Abstract in meeting |
| CTRI/2012/05/002656. A clinical trial to see the effects and safety of two drugs IVF-Mâ?¢ (Menotropin)and MenopurÂ® (Menotropin) in Infertile Women undergoing IVF treatment. 2012. | Trial register |
| CTRI/2017/11/010317. Comparative study of Recombinant human menopausal gonadotrophin in treatment of infertility. 2017. | Trial register |
| CTRI/2019/10/02179. Comparative study between two brands of human menopausal gonadotropin in infertility patients. 2019.  https://trialsearch.who.int/Trial2.aspx?TrialID=CTRI/2019/10/02179 | Wrong comparator: two different HP-hMGs |
| CTRI/2023/10/058780. To compare the success rate of Stop GnRH agonist-antagonist protocol with conventional IVF protocols. 2023.  https://trialsearch.who.int/Trial2.aspx?TrialID=CTRI/2023/10/058780 | Wrong comparator: Not HP-hMG |
| Daftary G S, Ando S, Yankov V, Heiser P W. Association between the number of oocytes retrieved and cumulative live birth rate in IVF treatment: Menopur in GNRH antagonist single embryo transfer-high responder (MEGASET-HR) trial outcomes. Fertility and Sterility. 2019;111(4 SUPPL):e12-. | Abstract in meeting |
| Dahan M H, Agdi M, Shehata F, Son W, Tan S L. A comparison of outcomes from in vitro fertilization cycles stimulated with either recombinant luteinizing hormone (LH) or human chorionic gonadotropin acting as an LH analogue delivered as human menopausal gonadotropins, in subjects with good or poor ovarian reserve: A retrospective analysis. European Journal of Obstetrics and Gynecology and Reproductive Biology. 2014;172(1):70-3. | Wrong comparator: Not HP-hMG |
| Dalal R J, Pai H, Palshetkar N. Effectiveness of HP-hMG vs r-FSH in patients undergoing IVF/ICSI cycles with moderate male factor infertility. International Journal of Infertility and Fetal Medicine. 2012;3(2):51-6. | Wrong study design |
| Dechaud H, Assou S, Moussaddykine S, Van Den Abbeel E, Aasted H, Hamamah S. Cumulus cells gene expression profile following controlled ovarian stimulation with HP-HMG or RFSH in a GnRH antagonist protocol: new indicators of ovarian microenvironment health. Fertility and Sterility. 2011;96(3): S257‐S258-. | Abstract in meeting |
| Del Gadillo J C, Siebzehnrübl E, Dittrich R, Wildt L, Lang N. Comparison of GnRH agonists and antagonists in unselected IVF/ICSI patients treated with different controlled ovarian hyperstimulation protocols: a matched study. Eur J Obstet Gynecol Reprod Biol. 2002;102(2):179-83. | Wrong comparator: Not HP-hMG. They compared GnRH agonists with antagonists |
| De Placido G, Mollo A, Alviggi C, Strina I, Varricchio M T, Ranieri A, et al. Rescue of IVF cycles by HMG in pituitary down-regulated normogonadotrophic young women characterized by a poor initial response to recombinant FSH. Human Reproduction. 2001;16(9):1875-9. | Wrong comparator: Not HP-hMG. |
| Deveer R, Karakas Yilmaz N, Col I, Batioglu S. Ovulation induction with HP-HMG versus recombinant FSH in PCO patients undergoing assisted reproduction cycles. Human reproduction. 2009;24 Suppl 1:i131 P‐325 Poster-. | Abstract in meeting |
| Dokuzeylül Güngör N, Yurci A, Gürbüz T, Güngör K. Comparing rLH with hMG in embryo transfers at the stage of blastocyst and pregnancy outcomes in poor responders. Journal of Experimental and Clinical Medicine (Turkey). 2021;38(4):474-7. | Wrong comparator: rFSH + u-hMG vs rFSH + rLH |
| Donato R, Bessow C, Genro V, Chapon R, Oliveira de Souza T, Cunha-Filho Jsld. Corifollitropin alpha was not detrimental to follicular ovarian responsiveness measured by follicular output rate (FORT). Hum Fertil (Camb). 2023;26(3):557-63. | Not RCT |
| Doody K, Daftary G S, Seifu Y, O'Brien K, Yankov V, Heiser P W. Can treatment of patients predicted to be high-responders be improved? insights from the Menopur in GNRH antagonist single embryo transfer - high responder (MEGASET-HR) trial. Fertility and Sterility. 2018;110(4 SUPPL):e31-. | Abstract in meeting |
| Drakakis P, Loutradis D, Kallianidis K, Milingos S, Dionyssiou-Asteriou A, Michalas S. The clinical efficacy of recombinant FSH (r-FSH) as compared to highly purified urinary gonadotrophin (hMG-FD) and the use of a low starting dose of r-FSH in IVF or ICSI. A randomized prospective study. Italian Journal of Gynaecology and Obstetrics. 2002;14(3):64-8. | Language exclusion |
| Drakakis P, Loutradis D, Kallianidis K, Liapi A, Milingos S, Makrigiannakis A, et al. Small doses of LH activity are needed early in ovarian stimulation for better quality oocytes in IVF-ET. European Journal of Obstetrics and Gynecology and Reproductive Biology. 2005;121(1):77-80. | Wrong comparator: rFSH (Puregon) vs rFSH + hMG (Menogon) |
| Duijkers I J, Willemsen W N, Hollanders H M, Hamilton C J, Thomas C M, Vemer H M. Follicular fluid hormone concentrations after ovarian stimulation using gonadotropin preparations with different FSH/LH ratios. II. Comparison of hMG and recombinant FSH. International Journal of Fertility and Women's Medicine. 1997;42(6):431‐435-. | Wrong comparator: Not HP-hMG |
| Duijkers I J, Willemsen W N, Hollanders H M, Hamilton C J, Thomas C M, Vemer H M. Follicular fluid hormone concentrations after ovarian stimulation using gonadotropin preparations with different FSH/LH ratios. I. Comparison of an FSH-dominant and purified FSH preparation. International Journal of Fertility and Women's Medicine. 1997;42(5):306‐310-. | Wrong comparator |
| Engel J B, Ludwig M, Felberbaum R, Albano C, Devroey P, Diedrich K. Use of Cetrorelix in combination with clomiphene citrate and gonadotrophins: A suitable approach to 'friendly IVF'? Human Reproduction. 2002;17(8):2022-6. | Wrong comparator: Not HP-hMG |
| Errázuriz J, Romito A, Drakopoulos P, Frederix B, Racca A, De Munck N, et al. Cumulative live birth rates following stimulation with corifollitropin alfa compared with HP-HMG in a GnRH antagonist protocol in poor ovarian responders. Frontiers in Endocrinology. 2019;10(MAR). | Not RCT |
| Eskandar M, Jaroudi K, Jambi A, Archibong E I, Coskun S, Sobande A A. Is recombinant follicle-stimulating hormone more effective in IVF poor responders than human menopausal gonadotrophins? Medical Science Monitor. 2004;10(1):PI6-9. | Wrong comparator: Not HP-hMG |
| Esteves S C, Schertz J C, Verza S Jr, Schneider D T, Zabaglia S F. A comparison of menotropin, highly-purified menotropin and follitropin alfa in cycles of intracytoplasmic sperm injection. Reprod Biol Endocrinol. 2009;7:111-. | Not RCT |
| EUCTR2004-001307-35-GB. A prospective, open label, randomised, parallel group, comparative pilot study to study the efficacy and safety of highly purified Menotrophin versus recombinant FSH (Follitropin alfa) administered subcutaneously to subfertile female patients undergoing IVF using antagonist downregulation. - PROSPECT. 2005. | Trial register |
| EUCTR2005-000993-29-IT. A prospective, randomized, investigator-blind, controlled, clinical study of phase III on the clinical efficacy and tolerability of hMG-IBSA IBSA Institut Biochimique sa versus Menopur Ferring administered subcutaneously in women undergoing controlled ovarian hyperstimulation COH in an ART programme IVF. 2007. | Trial register |
| EUCTR2008-008288-92-ES. Estudio clínico inicial para determinar las ventajas de emplear un preparado de FSH y LH recombinantes en proporción 2: 1 frente a la pauta habitual con menotropina (proporción: 1: 1). 2009. | Trial register |
| EUCTR2008-006775-67-SE. A randomised, open-label, assessor-blind, parallel groups, multicentre trial comparing the efficacy of MENOPUR versus recombinant FSH in controlled ovarian stimulation following a GnRH antagonist protocol and single embryo transfer - MEGASET. 2009. | Trial register |
| EUCTR2010-019411-37-DE. A Prospective, Open Label, Randomised, Parallel Group Trial Comparing the Effects of Highly Purified Menotrophin and Recombinant Follicle Stimulating Hormone (rFSH, Follitropin alpha) Administered Subcutaneoulsy to Subfertile Female Patients Undergoing IVF Using Antagonist Down-Regulation on Progesterone Serum Levels During the Follicular Phase and their Possible Use as Predictors for the Success Rate of Ongoing Pregnancies (PREDICT) - PREDICT. 2010. | Trial register |
| EUCTR2010-021021-13-GB. Safety and efficacy study comparing a new hMG formulation (hMG-IBSA) to a reference product (Menopur®) in patients undergoing ovarian stimulation for in vitro fertilisation (IVF). 2010. | Trial register |
| EUCTR2010-022032-37-IT. Randomized, open-label, single centre study on ovarian stimulation in ART poor responders treated with recombinant FSH in association with recombinant LH. 2011. | Trial register |
| EUCTR2013-000583-29-BE. Corifollitropin alfa followed by Menotropin for Poor Ovarian Responders Trial. 2013. | Trial register |
| EUCTR2013-002027-42-ES. Randomized Study stimulation of the ovaries in women with poor response expected or ovaries. 2013. | Trial register |
| EUCTR2013-002979-17-ES. Comparative study between the usage of corifollitropin alfa and daily recombinant FSH in the ovarian stimulation of low responders. 2013. | Trial register |
| EUCTR2014-001743-20-ES. Use of the corifolitropina alfa in oocyte donors. 2014. | Trial register |
| EUCTR2013-003817-16-PL. A study to compare Pergoveris and GONAL-f in women who have responded poorly to previous infertiliy treatment cycles. 2014. | Trial register |
| EUCTR2014-005331-14-ES. Type of gonadotropin and embryo kinetics of development. 2015. | Trial register |
| EUCTR2015-005762-28-ES. Comparative analysis of hormone variations during ovarian stimulation with two different stimulation drugs. 2016. | Trial register |
| Fábregues F, Creus M, Casals G, Carmona F, Balasch J. Outcome from consecutive ICSI cycles in patients treated with recombinant human LH and those supplemented with urinary hCG-based LH activity during controlled ovarian stimulation in the long GnRH-agonist protocol. Gynecological Endocrinology. 2013;29(5):430-5. | Not RCT |
| Feldberg D, Goldman J A, Shelef M, Ashkenazi J, Dicker D, Yeshaya A. Comparison of a fixed and dynamic protocol for embryo replacement in an IVF/ET programme. Human Reproduction. 1988;3(6):747-50. | Wrong comparator: Not HP-hMG neither rFSH |
| Fernandez-Ponce A, Yell D, Gregoire R, Drakeley A J. Evaluation of pregnancy and live birth outcomes at the Hewitt Fertility Centre for three newly introduced recombinant gonadotropins when compared to human menopausal gonadotropin. Human reproduction (Oxford). 2021;36(SUPPL 1):i419-. | Abstract in meeting |
| Figen Turkcapar A, Seckin B, Onalan G, Ozdener T, Batioglu S. Human Menopausal Gonadotropin versus Recombinant FSH in Polycystic Ovary Syndrome Patients undergoing In Vitro Fertilization. Int J Fertil Steril. 2013;6(4):238-43. | Wrong comparator: Not HP-hMG |
| Filicori M, Cognigni G, Melappioini Tabarelli C, Ferlini F, Bernardi S. Endocrine and clinical profiles of controlled ovarian stimulation (COS): improved features in human menopausal gonadotropin (hMG) vs recombinant human follicle-stimulating hormone (r-hFSH) treatment. Fertility and Sterility. 2002;78(3 Suppl 1):S104‐105, Abstract no: O‐275-. | Abstract in meeting |
| Fragoulakis V, Kourlaba G, Tarlatzis B, Mastrominas M, Maniadakis N. Economic evaluation of alternative assisted reproduction techniques in management of infertility in Greece. ClinicoEconomics and Outcomes Research. 2012;4(1):185-92. | Wrong study design |
| Fragoulakis V, Pescott C P, Smeenk J M, van Santbrink E J, Oosterhuis G J, Broekmans F J, et al. Economic Evaluation of Three Frequently Used Gonadotrophins in Assisted Reproduction Techniques in the Management of Infertility in the Netherlands. Appl Health Econ Health Policy. 2016;14(6):719-27. | Wrong outcomes |
| Frattarelli J L, Miller B T, Kaplan B, Widra E, Scott R T. Does leutinizing hormone activity in the form of low-dose HCG or HMG produce better outcomes for GnRH antagonist art cycles stimulated with rFSH? Fertility and Sterility. 2007;88 Suppl 1:132, Abstract no: 68-. | Abstract in meeting |
| Fried G, Harlin J, Csemiczky G, Wramsby H. Controlled ovarian stimulation using highly purified FSH results in a lower serum oestradiol profile in the follicular phase as compared with HMG. Human Reproduction. 1996;11(3):474-7. | Wrong comparator: Not HP-hMG |
| Fritzsche H, Michelmann H W, Siebzehnrübl E, Schmedemann R K A. Interactions between oocyte and surrounding cumulus cells influence the results of assisted reproduction. Journal fur Reproduktionsmedizin und Endokrinologie. 2006;3(6):373-8. | Wrong outcomes |
| Gatta V, Tatone C, Ciriminna R, Vento M, Franchi S, D'Aurora M, et al. Gene expression profiles of cumulus cells obtained from women treated with recombinant human luteinizing hormone + recombinant human follicle-stimulating hormone or highly purified human menopausal gonadotropin versus recombinant human follicle-stimulating hormone alone. Fertility and Sterility.2013;99(7):2000-2008.e1. | Wrong outcomes |
| Gizzo S, Andrisani A, Noventa M, Manfè S, Oliva A, Gangemi M, et al. Recombinant LH supplementation during IVF cycles with a GnRH-antagonist in estimated poor responders: A cross-matched pilot investigation of the optimal daily dose and timing. Mol Med Rep. 2015;12(3):4219-29. | Wrong study design |
| Gizzo S, Quaranta M, Andrisani A, Bordin L, Vitagliano A, Esposito F, et al. Serum stem cell factor assay in elderly poor responder patients undergoing IVF: A new biomarker to customize follicle aspiration cycle by cycle. Mol Med Rep. 2016;23(1):61-8. | Wrong outcomes |
| Goldfarb J M, Desai N. Follitropin-alpha versus human menopausal gonadotropin in an in vitro fertilization program. Fertil Steril. 2003;80(5):1094-9. | Wrong comparator: Not HP-hMG |
| Gomes M K, Vieira C S, Moura M D, Manetta L A, Leite S P, Reis R M, et al. Controlled ovarian stimulation with exclusive FSH followed by stimulation with hCG alone, FSH alone or hMG. European journal of obstetrics, gynecology, and reproductive biology. 2007;130(1):99‐106-. | Wrong comparator: Not HP-hMG |
| Gordon U D, Harrison R F, Fawzy M, Hennelly B, Gordon A C. A randomized prospective assessor-blind evaluation of luteinizing hormone dosage and in vitro fertilization outcome. Fertility and Sterility. 2001;75(2):324-31. | Wrong comparator: Not HP-hMG |
| Grover S A, Foster E D, Sinha A, Elci O U, Daftary G S, Heiser P W. Serum gonadotropin association with live birth in high-responders undergoing ovarian stimulation: Menopur in Gonadotropin Releasing Hormone (GNRH) antagonist single embryo transfer - high responder (MEGASET-HR) trial analysis. Fertility and Sterility. 2020;114(3):e92-. | Abstract in meeting |
| Grynberg M, Cedrin-Durnerin I, Raguideau F, Herquelot E, Luciani L, Porte F, et al. Comparative effectiveness of gonadotropins used for ovarian stimulation during assisted reproductive technologies (ART) in France: A real-world observational study from the French nationwide claims database (SNDS). Best Practice and Research: Clinical Obstetrics and Gynaecology. 2023;88. | Not RCT |
| Hammadeh M E, Ertan A K, Georg M T, Rosenbaum P, Schmidt W. Relationship between ovarian stimulation regimen and interleukin level in pre-ovulatory follicular fluid and their effect on ICSI outcome. American Journal of Reproductive Immunology. 2002;48(4):255-61. | Wrong comparator: Not HP-hMG |
| Hammadeh M E, Mauss V, Meisinger M, Herrmann W, Georg T, Rosenbaum P, et al. Association between ovarian stimulation regime and protein metabolism of patients undergoing IVF/ICSI therapy. Zentralblatt fur Gynakologie. 2003;125(5):183-8. | Wrong comparator: Not HP-hMG |
| Heiser P W, Foulk R, Seifu Y, Yankov V, Daftary G S. Highly purified human menotropin (HPHMG) is associated with a low incidence of ovarian hyperstimulation (OHSS) in patients undergoing invitro fertilization: Menopur in GNRH antagonist single embryo transfer-High responder (MEGASET-HR) trial outcomes. Human reproduction. 2018;109(3):e30‐e31-. | Abstract in meeting |
| Heiser P, Foster E, Sinha A, Elci O, Daftary G. Predicted high-responder women diagnosed with oligoovulation may benefit from stimulation with highly purified human menopausal gonadotrophin (HP-hMG). Human reproduction. 2020;35 Suppl 1:i6-. | Abstract in meeting |
| Helmgaard L, Klein B M, Arce J C. Twice-daily assessments of the local tolerability associated with a new MENOPUR multi-dose formulation during controlled ovarian stimulation. Human reproduction (Oxford). 2011;26:i236‐i237-. | Abstract in meeting |
| Hernández Ayup S, Balderas Rosales C D R, De La Llata E S, Moraga M R, Batiza Reséndiz V, Santos Haliscak R, et al. Intacytoplasmic sperm injection: Reproductive reality in couples with sterility. Ginecologia y Obstetricia de Mexico. 2005;73(2):69-75. | Wrong study design |
| Hu Z, Zeng R, Gao R, Chen M, Liu X, Zhang Q, et al. Effects of different gonadotropin preparations in GnRH antagonist protocol for patients with polycystic ovary syndrome during IVF/ICSI: a retrospective cohort study. Front Endocrinol (Lausanne). 2024;15:1309993-. | Not RCT |
| Humaidan P, Schertz J, Fischer R. Efficacy and safety of pergoveris in assisted reproductive technology- ESPART: Rationale and design of a randomised controlled trial in poor ovarian responders undergoing IVF/ ICSI treatment. BMJ Open. 2015;5(7). | Wrong study design |
| Humaidan P, Chin W, Rogoff D. Results of the ESPART randomized controlled trial investigating recombinant luteinizing hormone supplementation for controlled ovarian stimulation in poor ovarian responders aligned with the Bologna criteria. Human reproduction (Oxford). 2016;31:i15‐i16-. | Abstract in meeting |
| Hussain S S, Hassan M F. The effectiveness of using r-HMG+r-FSH vs. R-FSH alone during COS on ICSI outcome. Annals of Tropical Medicine and Public Health. 2019;22(8). | Abstract in meeting |
| Hwang F R, Chang M Y, Soong Y K. Gonadotropin stimulation after pituitary desensitization with leuprolide acetate, comparison of FSH/hMG and hMG alone cycles - A study of 166 cases. Chang Gung Medical Journal. 1993;16(4):223-30. | Wrong comparator: Not HP-hMG |
| Ilhana G A, Erkanli Senturka G, Oktemb O, Durmusoglua F. The impact of HMG on follicular fluid hormone levels, embryo quality and IVF outcome. Eastern Journal of Medicine. 2014;19(1):22-7. | Wrong comparator: Not HP-hMG |
| IRCT201201046541N2. Comparison of different medication protocols for induction of ovarian in the cycles of in vitro fertilization on expression of GREM 1, HAS2 and PGS2 genes as determinants of oocyte development. 2012. | Trial register |
| IRCT201408116541N7. Comparison of ovulation induction with different gonodotropins. 2014. | Trial register |
| IRCT2016092720351N3. Comparison of the Clinical pregnancy outcomes of ovarian stimulation with hMG + rFSH or rFSH + rLH methods in infertile patients. 2016. | Trial register |
| Jansen C A, van Os H C, Out H J, Coelingh Bennink H J. A prospective randomized clinical trial comparing recombinant follicle stimulating hormone (Puregon) and human menopausal gonadotrophins (Humegon) in non-down-regulated in-vitro fertilization patients. Human reproduction (Oxford). 1998;13(11):2995‐2999-. | Wrong comparator: Not HP-hMG |
| Janssens L, Roelant E, De Neubourg D. The LH endocrine profile in gonadotropin-releasing hormone analogue cycles. Gynecological Endocrinology. 2022;38(10):831-9. | Wrong outcomes |
| Ji Z, Quan X, Lan Y, Zhao M, Tian X, Yang X. Gonadotropin versus Follicle-Stimulating Hormone for Ovarian Response in Patients Undergoing in vitro Fertilization: A Retrospective Cohort Comparison. Current Therapeutic Research - Clinical and Experimental. 2020;92. | Not RCT |
| Kably A, Castelazo E, Barroso G. [Comparative analysis of multifollicular development with the application of recombinant FSH vs. urinary FSH in the results of in vitro fertilization]. Ginecol Obstet Mex. 2001;69:304-9. | Wrong comparator: not HP-hMG |
| Kan O, Simsir C, Atabekoglu C S, Sonmezer M. The impact of adding hp-hMG in r-FSH started GnRH antagonist cycles on ART outcome. Gynecological Endocrinology. 2019;35(10):869-72. | Not RCT |
| Karlström P O, Holte J, Hadziosmanovic N, Rodriguez-Wallberg K A, Olofsson J I. Does ovarian stimulation regimen affect IVF outcome? a two-centre, real-world retrospective study using predominantly cleavage-stage, single embryo transfer. Reproductive BioMedicine Online. 2018;36(1):59-66. | Not RCT |
| Khair A F, Nelson W, Sinha A, Ando M, Heiser P W, Robins J C, et al. Can treatment choice affect cost of therapy in patients predicted to be high-responders? Results of an economic analysis of the Menopur in GnRH antagonist single embryo transfer - high responder (MEGASET-HR) trial. Fertility and Sterility. 2019;112(3):e218-. | Abstract in meeting |
| Khair A F, Foster E D, Sinha A, Elci O U, Daftary G S, Heiser P W. Differential ovarian response to gonadotropin preparations despite similar ovarian reserve: Menopur in GNRH (Gonadotropin Releasing Hormone) antagonist single embryo transfer - high responder (MEGASET-HR) trial analysis. Fertility and Sterility. 2020;114(3):e92-. | Abstract in meeting |
| Khair A F, Brown T, Markert M, Samuelsen C H, Barsoe C, Daftary G S, et al. Cost comparison of highly purified Human Menopausal Gonadotropin (HP-HMG) versus recombinant Follicle-Stimulating Hormone (rFSH) for controlled ovarian stimulation in us high-responder patients based on the MEGASET-HR trial. Fertility and Sterility. 2021;116(3):e267-. | Abstract in meeting |
| Khair A, Brown T, Markert M, Barsøe C R, Daftary G S, Heiser P W. Highly Purified Human Menopausal Gonadotropin (HP-hMG) Versus Recombinant Follicle-Stimulating Hormone (rFSH) for Controlled Ovarian Stimulation in US Predicted High-Responder Patients: A Cost-Comparison Analysis. PharmacoEconomics Open 7, 851–860 (2023). | No additional data to included studies |
| Khlifi A, Kacem O, Maroueni M, Elgoul L, Hidar S, Fekih M, et al. The influence of gonadotropins on clinico-biological ICSI outcome: A retrospective comparative study rFSH vs HP-hMG. Tunisie Medicale. 2016;94(6):167-77. | Not RCT |
| Kilani Z, Dakkak A, Ghunaim S, Cognigni G, Melappioni S, Filicori M. Elevated efficacy of highly purified human menopausal gonadotropin (HP hMG) for intracytoplasmic sperm injection (ICSI): results of a prospective, randomized, controlled trial comparing HP hMG to recombinant Follicle-Stimulating hormone (r-hFSH). Fertility and Sterility. 2002;78(3 Suppl 1):S53, Abstract O‐138-. | Abstract in meeting |
| Kim Y J, Park K A, Chae S J, Lim K S, Hur C Y, Kang Y J, et al. Effects of LH activity during ovarian stimulation for IVF according to their types in old women. Human reproduction (Oxford). 2011;26:i312-. | Abstract in meeting |
| Kirshenbaum M, Gil O, Haas J, Nahum R, Zilberberg E, Lebovitz O, et al. Recombinant follicular stimulating hormone plus recombinant luteinizing hormone versus human menopausal gonadotropins- does the source of LH bioactivity affect ovarian stimulation outcome? Reproductive Biology and Endocrinology. 2021;19(1). | Not RCT |
| Konstantinidou F, Placidi M, Di Emidio G, Stuppia L, Tatone C, Gatta V, et al. Maternal MicroRNA profile changes when LH Is added to the ovarian stimulation protocol: A Pilot Study. Epigenomes. 2023;7(4). | Wrong study design |
| Koo H S, Kwon H, Choi D S, Han S, Seo J Y, Yang K M. Clinical utility of newly developed highly purified human menopausal gonadotrophins: a randomized controlled trial. Reproductive BioMedicine Online. 2017;34(5):499-505. | Wrong comparator: two different HP-hMGs are compared |
| Kumbak B, Kahraman S. Effect of combining recombinant FSH with recombinant LH on oocyte and embryo quality in the GnRH agonist long and antagonist cycles. Journal of the Turkish German Gynecology Association. 2008;9(3):120-6. | Not RCT |
| Laufer N, DeCherney A H, Tarlatzis B C, Zuckerman A L, Polan M L, Dlugi A M, et al. Delaying human chorionic gonadotropin administration in human menopausal gonadotropin-induced cycles decreases successfully in vitro fertilization of human oocytes. Fertility and Sterility. 1984;42:198‐203-. | Wrong comparator: not HP-hMG |
| Lavy G, Pellicer A, Diamond M P, DeCherney A H. Ovarian stimulation for in vitro fertilization and embryo transfer, human menopausal gonadotropin versus pure human follicle stimulating hormone: A randomized prospective study. Fertility and Sterility. 1988;50(1):74-8. | Wrong comparator: not HP-hMG neither rFSH |
| Lezama-Ruvalcaba J L, Salazar-López Ortiz C G, Téllez-Velasco S, Bahena-Espinoza N. Results in IVF-ICSI cycles adding luteinizing hormone recombinant to follicle stimulating hormone recombinant with menotropins during ovarian stimulation in patients over 35 years-old. Ginecologia y Obstetricia de Mexico. 2018;86(6):383-8. | Not RCT |
| Li N, Shi J, Tian L, Yu L, Wang T, Meng B, et al. Recombinant FSH plus highly purified hMG versus recombinant FSH on ovarian response and clinical outcomes in long GnRH agonist protocol: a prospective, randomized, controlled trial. Human reproduction (Oxford). 2016;31:i420-. | Abstract in meeting |
| Lisi F, Rinaldi L, Fishel S, Lisi R, Pepe G, Picconeri M G, et al. Use of recombinant FSH and recombinant LH in multiple follicular stimulation for IVF: a preliminary study. Reprod Biomed Online. 2001;3(3):190-4. | Not RCT |
| Lisi F, Rinaldi L, Fishel S, Lisi R, Pepe G P, Picconeri M G, et al. Use of recombinant LH in a group of unselected IVF patients. Reproductive BioMedicine Online. 2002;5(2):104‐108-. | Not RCT |
| Lisi F, Rinaldi L, Fishel S, Caserta D, Lisi R, Campbell A. Evaluation of two doses of recombinant luteinizing hormone supplementation in an unselected group of women undergoing follicular stimulation for in vitro fertilization. Fertil Steril. 2005 Feb;83(2):309-15. | Wrong comparator: two doses of recombinant (r)LH, 75 IU (recommended) or 37.5 IU |
| Lloyd A, Kennedy R, Hutchinson J, Sawyer W. Economic evaluation of highly purified menotropin compared with recombinant follicle-stimulating hormone in assisted reproduction. Fertility and Sterility 2003;80(5):1108-13. | Wrong outcomes |
| Lou H Y, Huang X Y. Modified natural cycle for in vitro fertilization and embryo transfer in normal ovarian responders. Journal of International Medical Research. 2010;38(6):2070-6. | Wrong comparator: not HP-hMG, and the comparison was hMG vs rFSH + triptorelin |
| Loumaye E, Engrand P, Shoham Z, Hillier S G, Baird D T. Clinical evidence for an LH 'ceiling' effect induced by administration of recombinant human LH during the late follicular phase of stimulated cycles in World Health Organization type I and type II anovulation. Human Reproduction. 2003;18(2):314-22. | Wrong outcomes |
| Loutradis D, Stefanidis K, Drakakis P, Kallianidis K, Kallipolitis G, El Sheih A, et al. Does the addition of menopausal gonadotropin to recombinant FSH in pituitary suppressed women improve clinical pregnancy in an intracytoplasmic sperm injection program? Middle East Fertility Society Journal. 2003;8(1):30-5. | Wrong comparator: not HP-hMG |
| Loutradis D, Elsheikh A, Kallianidis K, Drakakis P, Stefanidis K, Milingos S, et al. Results of controlled ovarian stimulation for ART in poor responders according to the short protocol using different gonadotrophins combinations. Archives of Gynecology and Obstetrics. 2004;270(4):223‐226-. | Wrong comparator |
| Ludwig M, Strik D, Felberbaum R, Al-Hasani S, Diedrich K. No significant leukocytosis under controlled ovarian stimulation using the LHRH antagonist Cetrorelix and recFSH. European Journal of Obstetrics and Gynecology and Reproductive Biology. 2000;89(2):177-9. | Wrong comparator: not HP-hMG |
| Mahmoud K, Zhioua F, Kefi Attaoui L, Ben Aribia M, Meherzi F, Nemsia J, et al. Controlled ovarian stimulation (COS) in assisted reproductive technologies: rFSH alone or RFSH and HMG combined? Human reproduction (Oxford). 2001;16(Suppl 1):92-. | Abstract in meeting |
| Maity S P, Ghoshdastidar B, Ghoshdastidar S. A comparative study to examine the relative efficiency of hMG versus recombinant LH for LH activity supplementation in recombinant FSH cycles in art. Human reproduction (Oxford). 2010;25 suppl 1(6):i314‐i315-. | Abstract in meeting |
| Marcus S F, Brinsden P R, Macnamee M, Rainsbury P A, Elder K T, Edwards R G. Comparative trial between an ultra-short and long protocol of luteinizing hormone-releasing hormone agonist for ovarian stimulation in in-vitro fertilization. Human Reproduction. 1993;8(2):238-43. | Wrong comparator: long vs short GnRH agonist |
| Martin-Johnston M, Beltsos A N, Grotjan H E, Uhler M L. Adding human menopausal gonadotrophin to antagonist protocols - is there a benefit? Reprod Biomed Online. 2007;15(2):161-8. | Wrong comparator: not HP-hMG |
| Mayenga J M, Belaisch-Allart J, Chouraqui A, Tesquier L, Serkine A M, Cohen J, et al. Comparative randomized controlled study between human follicle- stimulating hormone (FSH-HP) and human menopausal gonadotropins (hMG) in in vitro fertilization. Contraception, fertilite, sexualite (1992). 1997;25(5):371‐374-. | Wrong comparator: not HP-hMG |
| Mayenga J, Belaisch-Allart J, Grefenstette I, Chouraqui A, Serkine A A M, Abirached F, et al. Comparison of in vitro fertilization (IVF) outcomes in patients treated with hMG vs. recombinant FSH in their first IVF cycle. Human reproduction (Oxford). 2006;21(Suppl):i126-. | Abstract in meeting |
| Mendret-Pellerin S, Leperlier F, Reignier A, Lefebvre T, Barrière P, Fréour T. A pilot study comparing corifollitropin alfa associated with hp-HMG versus high dose rFSH antagonist protocols for ovarian stimulation in poor responders. Hum Fertil (Camb). 2020;23(2):93-100. | Wrong study design |
| Mennini F S, Marcellusi A, Viti R, Bini C, Carosso A, Revelli A, et al. Probabilistic cost-effectiveness analysis of controlled ovarian stimulation with recombinant FSH plus recombinant LH vs. Human menopausal gonadotropin for women undergoing IVF. Reproductive Biology and Endocrinology. 2018;16(1). | Wrong study design |
| Meo F, Ranieri D M, Khadum I, Serhal P. Ovarian response and in vitro fertilization outcome in patients with reduced ovarian reserve who were stimulated with recombinant follicle-stimulating hormone or human menopausal gonadotropin. Fertility and Sterility. 2002;77(3):630-2. | Wrong comparator: not HP-hMG |
| Mignini Renzini M, Brigante C, Coticchio G, Dal Canto M, Caliari I, Comi R, et al. Retrospective analysis of treatments with recombinant FSH and recombinant LH versus human menopausal gonadotropin in women with reduced ovarian reserve. Journal of Assisted Reproduction and Genetics. 2017;34(12):1645-51. | Not RCT |
| Moraga R, Saucedo E, García-Gimeno T, Rodenas J J, Monzó A, Romeu A. Comparative study between two pituitary suppression protocols in controlled ovarian hyperstimulation for in vitro fertilization. Revista Iberoamericana de Fertilidad y Reproduccion Humana. 2003;20(5):283-9. | Wrong comparator: Nafarelin vs Cetrorelix |
| Motan T, Kiddoo D A. Randomised study of clinical pregnancy rates using different gonadotropins in IVF/ICSI cycles. Fertility and Sterility. 2011;96(3):S175-. | Abstract in meeting |
| Muñoz M, Cruz M, Humaidan P, Garrido N, Pérez-Cano I, Meseguer M. Dose of recombinant FSH and oestradiol concentration on day of HCG affect embryo development kinetics. Reprod Biomed Online. 2012;25(4):382-9. | Wrong study design |
| Munz W, Fischer-Hammadeh C, Herrmann W, Georg T, Rosenbaum P, Schmidt W, et al. Body mass index, protein metabolism profiles and impact on IVF/ICSI procedure and outcome. Zentralblatt fur Gynakologie. 2005;127(1):37-42. | Wrong comparator: not HP-hMG |
| Musters A, Wely van M, Verhoeve H, Repping S, Veen Van Der F, Mochtar M H. The effect of rLH-addition to rFSH for controlled ovarian hyperstimulation in women with poor ovarian reserve on embryo-quality, a randomized control trial. Human reproduction (Oxford). 2011;26:i2-. | Abstract in meeting |
| Nakagawa K, Ohgi S, Kojima R, Sugawara K, Horikawa T, Ito M, et al. Recombinant follicle stimulating hormone is more effective than urinary human menopausal gonadotropin in ovarian hyperstimulation for assisted reproductive technology treatment. Reproductive Medicine and Biology. 2007;6(1):27-32. | Wrong comparator: not HP-hMG |
| Nassar Z, Massad Z, Abdo G, Fakih M. Ovarian stimulation for in vitro fertilization (IVF): a prospective randomized comparison of recombinant FSH alone or in combination with human menopausal gonadotropins. Fertility and Sterility. 2001;76(3 Suppl 1):S92-. | Abstract in meeting |
| Nazarenko T, Durinian E, Chechurova T. The advantages of ovulation induction using recombinant FSG in comparison with human menopausal gonadotropin treatment of anovulatory infertility. XVI FIGO world congress of O & G. 2000; Abstract book 1:36‐37-. | Abstract in meeting |
| Nazzaro A, Salerno A. Recombinant LH supplementation to recombinant FSH during induced ovarian stimulation in the GnRH-antagonist protocol improves implantation and pregnancy rates. Fertility and Sterility. 2012;98 Suppl 1(3): S280-. | Abstract in meeting |
| Nazzaro A, Salerno A, Di Iorio L, Landino G, Marino S, Pastore E. RLH supplementation to rFSH during induced ovarian stimulation in the GnRh antagonist protocol improves implantation and pregnancy rates. Human reproduction (Oxford). 2013;28: i311-. | Abstract in meeting |
| NCT00335894. Clinical study of clinical efficacy and tolerability of hMG-IBSA s.c.in women undergoing COH in an ART programme (IVF). 2006. | Trial register |
| NCT00334425. The effect of LH-Priming during early follicular phase in IVF treatment. 2006. | Trial register |
| NCT00670059. Pre-Implantation genetic screening in women under the age of 36 years with single embryo transfer. 2008. | Wrong study design |
| NCT00805935. Menopur® Versus Follistim® in Polycystic Ovarian Syndrome (PCOS). 2008. | Trial register |
| NCT00669786. Human Menopausal Gonadotropin (HMG) vs Recombinant Follicle Stimulating Hormone (rFSH) in Gonadotropin Releasing Hormone (GnRH) antagonist cycles. 2008. | Trial register |
| NCT00884221. MENOPUR in Gonadotrophin-releasing Hormone (GnRH) antagonist cycles with single embryo transfer. 2009. | Trial register |
| NCT00829075. Impact of three different gonadotrophin regimes on egg donation program. 2009 | Trial register |
| NCT01365936. hMG or Recombinant FSH on OHSS prevention in PCOS patients undergoing IVF. 2011. | Trial register |
| NCT01297465. PERgoveriS in stratified treatment for assisted reproductive technique. 2011. | Trial register |
| NCT01312766. Safety and efficacy studyin Vitro Fertilisation (IVF) Patients. 2011. | Wrong comparator: Compare two different HP-hMGs |
| NCT01623570. Clinical outcomes in WHO Type I anovulatory women using r-hFSH+r-hLH in a 2: 1 ratio or hMG-HP. 2012. | Trial register |
| NCT02412904. Randomized controlled trial comparing embryonic quality in rFSH versus hMG in IVF protocol with GnRH antagonist. 2013. | Trial register |
| NCT02244866. Efficacy of Pergoveris in aged IVF patients. 2014. | Trial register |
| NCT02047227. Phase 3 Study to evaluate the efficacy and safety of Pergoveris® in assisted reproductive technology (ESPART). 2014. | Trial register |
| NCT02118051. Effect of treatment with Corifollitropin Alpha in Vitro Fertilization in patients with poor ovarian response. 2014. | Trial register |
| NCT02254928. Corifollitropin Alfa versus daily rFSH in the controlled ovarian stimulation of poor responders. 2014. | Wrong comparator: Corifollitropin alfa versus daily recombinant FSH and hMG |
| NCT02069808. Efficacy of recombinant FSH/GnRH antagonist protocol with and without LH adjunct for egg bank donation. 2014. https://clinicaltrials.gov/show/NCT02069808 | Trial register |
| NCT02418533. Mono-Menotropins versus rFSH Protocol on Embryo Quality. 2015. | Trial register |
| NCT02458768. Multicenter, randomized, open label, parallel study to evaluate the efficacy & safety of IVF-M HP Inj. vs. Menopur® Inj. 2015. | Trial register |
| NCT02554279. MENOPUR® in a Gonadotropin-Releasing Hormone (GnRH) antagonist cycle with single-blastocyst transfer in a high responder subject population. 2015. | Trial register |
| NCT03134690. GnRh antagonist protocol with delayed start stimulation in patients with poor ovarian response. 2016. | Trial register |
| NCT02677259. luteal phase estradiol support for in vitro fertilization/ intracytoplasmic sperm injection cycles. 2016. | Wrong comparator: address the role of estradiol luteal phase supplementation. For COS, rFSH and rhCG and/or medications containing both FSH and LH were used. |
| NCT02738580. Follicular steroid genesis in controlled ovarian stimulation. 2016. | Trial register |
| NCT04549649. The effect of dual trigger for final oocyte maturation on IVF/ICSI outcomes in patients with suboptimal ovarian response. 2020. | Trial register |
| Ng E H, Lau E Y, Yeung W S, Ho P C. HMG is as good as recombinant human FSH in terms of oocyte and embryo quality: a prospective randomized trial. Human reproduction (Oxford) 2001;16(2):319‐325-. | Wrong comparator: Not HP-hMG |
| NTR1457. L-AGE study. 2008. https://trialsearch.who.int/Trial2.aspx?TrialID=NTR1457 | Wrong comparator: different doses of rFSH |
| Nyboe Andersen A, Humaidan P, Fried G. Addition of rLH (Luveris) to rFSH during the fiinal days of follicular maturation in IVF/ICSI treated patients. A Nordic randomized multicentre trial. Human reproduction (Oxford). 2006;21(Suppl):i54-. | Abstract in meeting |
| Nyboe Andersen A, Devroey P, Arce J C. Live birth rate after single blastocyst transfer in a GnRH antagonist cycle using highly purified meno-tropin or recombinant FSH for controlled ovarian stimulation. Fertility and Sterility. 2011;96(3): S176-. | Abstract in meeting |
| Oka A A, Oka G A. The enigma of early progesterone rise: Is it associated with the type of gonadotropin used? Journal of Human Reproductive Sciences. 2019;12(3):229-33. | Wrong comparator: not HP-hMG |
| Olivennes F, Belaich Allart J, Alvarez S, Bouchard P, Frydman R. The use of hMG versus rec-FSH with the single dose GnRH antagonist (Cetrorelix) protocol in IVF-ET: a prospective randomized study. Fertility and Sterility. 1999;72(3 Suppl 1):S114‐5-. | Abstract in meeting |
| Orvieto R, Homburg R, Meltcer S, Rabinson J, Anteby E Y, Nahum R. HMG improves IVF outcome in patients with high basal FSH/LH ratio: A preliminary study. Reproductive BioMedicine Online. 2009;18(2):205-8. | Wrong comparator: not HP-hMG |
| Orvieto R. HMG versus recombinant FSH plus recombinant LH in ovarian stimulation for IVF: does the source of LH preparation matter? Reproductive BioMedicine Online. 2019;39(6):1001-6. | Wrong study design |
| Out H J, Driessen S G, Mannaerts B M, Coelingh Bennink H J. Recombinant follicle-stimulating hormone (follitropin beta, Puregon) yields higher pregnancy rates in in vitro fertilization than urinary gonadotropins. Fertility and Sterility. 1997;68(1):138‐142-. | Wrong comparator: not HP-hMG |
| Out H J, Driessen Sgaj, Mannaerts Bmjl, Coelingh Bennink H J T. Recombinant follicle-stimulating hormone (follitropin beta, Puregon) yields higher pregnancy rates in vitro fertilization than urinary gonadotropins. Fertility and Sterility. 1998;69(2 SUPPL.1):40S‐44S-. | Wrong comparator: not HP-hMG |
| Parsanezhad M E, Jahromi B N, Rezaee S, Kooshesh L, Alaee S. The effect of four different gonadotropin protocols on oocyte and embryo quality and pregnancy outcomes in IVF/ICSI cycles; a randomized controlled trial. Iranian Journal of Medical Sciences. 2017;42(1):57-65. | Wrong comparator: not HP-hMG |
| Paterson N D, Foong S C, Greene C A. Improved pregnancy rates with luteinizing hormone supplementation in patients undergoing ovarian stimulation for IVF. Journal of Assisted Reproduction and Genetics. 2012;29(7):579-83. | Not RCT |
| Pelekanos M, Gregorakis S, Pistofidis G, Tsirigotis M. Preliminary data on the use of rFSH versus rFSH plus HMG in patients undergoing assisted conception with IVF and embryo transfer. Human reproduction (Oxford). 2000;15(1):151-. | Abstract in meeting |
| Petanovski Z, Dimitrov G, Aydin B, Hadzi-Lega M, Sotirovska V, Suslevski D, et al. Recombinant FSH versus HP-HMG for controled ovarian stimulation in intracitoplasmic sperm injection cycles. Med Arh. 2011;65(3):153-6. | Wrong study design |
| Platteau P, Andersen A N, Balen A, Devroey P, Sørensen P, Helmgaard L, et al. Similar ovulation rates, but different follicular development with highly purified menotrophin compared with recombinant FSH in WHO Group II anovulatory infertility: A randomized controlled study. Reproductive BioMedicine Online. 2006;21(7):1798-804. | Wrong outcomes |
| Platteau P, Andersen A N, Loft A, Smitz J, Danglas P, Devroey P. Highly purified HMG versus recombinant FSH for ovarian stimulation in IVF cycles. Reprod Biomed Online. 2008 Aug;17(2):190-8. | No additional data to included studies |
| Platteau P, Helmgaard L, Arce J C. Interplay between infertility diagnosis and type of gonadotropin with respect to treatment outcome of controlled ovarian stimulation. Human reproduction (Oxford). 2009;92(3):S149-. | Abstract in meeting |
| Platteau P, Bosch E, Klein B M, Arce J C. Relation between serum progesterone at the end of controlled ovarian stimulation and treatment outcome across type of gonadotropin preparations. Human reproduction (Oxford). 2014;29:i322-. | Abstract in meeting |
| Prajapati K, Desai M, Shah S, Choudhary S, Aggarwal R, Mishra V. Treatment outcome of ovulation-inducing agents in patients with anovulatory infertility: A prospective, observational study. Journal of Pharmacology and Pharmacotherapeutics. 2017;8(3):116-21. | Wrong comparator: not HP-hMG |
| Raju G A R, Teng S C, Kavitha P, Lakshmi B K, Ravikrishna C. Combination of recombinant follicle stimulating hormone with human menopausal gonadotrophin or recombinant luteinizing hormone in a long gonadotrophin-releasing hormone agonist protocol: A retrospective study. Reproductive Medicine and Biology. 2012;11(3):129-33. | Not RCT |
| Rao KA, Khanna G, Bavishi H, Reddy NS, Mangukiya K, Jain R, Pv K. Clinical efficacy and safety of two highly purified human menopausal gonadotropins in women undergoing in vitro fertilization. Reprod Fertil. 2025 Jun 10;6(2):e240132. | Wrong comparator: two highly purified gonadotropins are compared for COS |
| Rashidi B H, Sarvi F, Tehrani E S, Zayeri F, Movahedin M, Khanafshar N. The effect of HMG and recombinant human FSH on oocyte quality: A randomized single-blind clinical trial. European Journal of Obstetrics and Gynecology and Reproductive Biology. 2005;120(2):190-4. | Wrong comparator: not HP-hMG |
| Requena A, García-Velasco J A, Coroleu B, Alarcón M, Alberto-Bethencourt J C, Báez D, et al. Erratum: Evaluation of the effectiveness and clinical security of Menopur® in the ovarian stimulation in IVF-ICSI. Revista Iberoamericana de Fertilidad y Reproduccion Humana. 2006;23(5):333-. | Wrong study design |
| Requena A, García-Velasco J A, Coroleu B, Alarcón M, Alberto-Bethencourt J C, Báez D, et al. Evaluation of the effectiveness and clinical security of Menopur® in the ovarian stimulation in IVF-ICSI. Revista Iberoamericana de Fertilidad y Reproduccion. Human2006;23(4):245-55. | Wrong study design |
| Requena A, Landeras J L, Martinez-Navarro L, Calatayud C, Sanchez F, Maldonado V, et al. Could the addition of hp-hMG and GnRH antagonists modulate the response in IVF-ICSI cycles? Human fertility (Cambridge). 2010;13(1):41‐49-. | Not RCT |
| Requena A, Cruz M, Ruiz F J, García-Velasco J A. Endocrine profile following stimulation with recombinant follicle stimulating hormone and luteinizing hormone versus highly purified human menopausal gonadotropin. Reproductive Biology and Endocrinology. 2014;12(1). | Not RCT |
| Revelli A, Pettinau G, Basso G, Carosso A, Ferrero A, Dallan C, et al. Controlled Ovarian Stimulation with recombinant-FSH plus recombinant-LH vs. human Menopausal Gonadotropin based on the number of retrieved oocytes: Results from a routine clinical practice in a real-life population. Reprod Biol Endocrinol 13, 77 (2015). | No additional data to included studies |
| Ritzinger A, Schmidmayr M, Lesoine B, Seifert-Klauss V. Influence of short-term ovarian stimulation on bone metabolism in women undergoing fertility treatment. Journal of Laboratory Medicine. 2021;45(1):19-26. | Wrong outcomes |
| Robins J C, Khair A F, Widra E A, Alper M M, Nelson W W, Foster E D, et al. Economic evaluation of highly purified human menotropin or recombinant follicle-stimulating hormone for controlled ovarian stimulation in high-responder patients: analysis of the Menopur in Gonadotropin-releasing Hormone Antagonist Single Embryo Transfer-High Responder (MEGASET-HR) trial. F S Rep. 2020;1(3):257-63. | No additional data to included studies |
| Rossin B, Pouly J L, Belaisch-Allart J, de Mouzon J. [Ovarian stimulation for IVF in France: choice and results according to protocols and gonadotrophin]. Gynecol Obstet Fertil. 2009;37(11-12):864-72. | Wrong comparator: not HP-hMG |
| Sabouni R Skorupski J Witz C A Wang W H Williams D B. Human menopausal gonadotropin vs. mixed protocol (HMG/FSH) in IVF/PGS: effects on embryo quality and euploidy. Fertility and Sterility. 2015;103 Suppl 2:e33-. | Abstract in meeting |
| Sagnella F, Moro F, Lanzone A, Tropea A, Martinez D, Capalbo A, et al. A prospective randomized noninferiority study comparing recombinant FSH and highly purified menotropin in intrauterine insemination cycles in couples with unexplained infertility and/or mild-moderate male factor. Fertility and Sterility. 2011;95(2):689-94. | Wrong patient population |
| Saucedo de la Llata E, Moraga Sánchez M R, Pezino Rodríguez J, Treviño A, Leal Almeida M, Sepúlveda J, et al. [High concentrations of serum estradiol in assisted reproduction]. Ginecol Obstet Mex. 2003;71:585-9. | Wrong comparator: assessment of the serum estradiol |
| Saucedo De La Llata E, Moraga Sánchez M R, Batiza Reséndiz V, Santos Haliscak R, Galache Vega P, Hernández Ayup S. Comparison of GnRH agonists and antagonists in an ovular donation program. Ginecologia y Obstetricia de Mexico. 2004;72(2):53-6. | Wrong comparator : Leuprolide vs Cetrorelix |
| Schwarze J E, Crosby J A, Zegers-Hochschild F. Addition of neither recombinant nor urinary luteinizing hormone was associated with an improvement in the outcome of autologous in vitro fertilization/intracytoplasmatic sperm injection cycles under regular clinical settings: a multicenter observational analysis. Fertility and Sterility. 2016;106(7):1714‐1717.e1-. | Not RCT |
| Serhal P, Phopong P, Ranieri D M. Comparison between human menopausal gonadotrophin and recombinant FSH for ovarian stimulation in patients undergoing in-vitro fertilization. Human reproduction (Oxford). 2000;15(1):143-. | Abstract in meeting |
| Setti A S, Braga Dpaf, Iaconelli A Júnior, Borges E Júnior. Improving Implantation Rate in 2nd ICSI Cycle through Ovarian Stimulation with FSH and LH in GNRH Antagonist Regimen. 2021;43(10):749-58. | Wrong study design |
| Sharara F I, Foster E D, Sinha A, Daftary G S, Heiser P W. Gonadotropin-specific follicular steroidogenesis in ovarian stimulation: evidence from the menopur in GnRH antagonist single embryo transfer - high responder (MEGASET-HR) trial. Fertility and Sterility. 2019;112(3):e56-. | Abstract in meeting |
| Shavit T, Agdi M, Son W Y, Hasson J, Dahan M H. A comparison between r-LH and urinary supplements containing LH activity in patients undergoing the microdose GnRH agonist flare protocol for in-vitro fertilization: A pilot study. Minerva Ginecologica. 2016;68(4):393-9. | Wrong comparator: not HP-hMG |
| Sills E S, Schattman G L, Veeck L L, Liu H C, Prasad M, Rosenwaks Z. Characteristics of consecutive in vitro fertilization cycles among patients treated with follicle-stimulating hormone (FSH) and human menopausal gonadotropin versus FSH alone. Fertility and Sterility. 1998;69(5):831-5. | Wrong comparator: not HP-hMG neither rFSH |
| Slater C C, Elci O U, Foster E D, Daftary G S, Heiser P W. Evaluation of possible factors contributing to early pregnancy loss in predicted high-responders: Menopur in GNRH (Gonadotropin Releasing Hormone) antagonist single embryo transfer - high responder (MEGASET-HR) trial analysis. Fertility and Sterility. 2021;116(3):e26-. | Abstract in meeting |
| Sopa N, Larsen E C, Westring Hvidman H, Andersen A N. An AMH-based FSH dosing algorithm for OHSS risk reduction in first cycle antagonist protocol for IVF/ICSI. European Journal of Obstetrics and Gynecology and Reproductive Biology. 2019;237:42-7. | Not RCT |
| Souza P M G, Carvalho B R, Nakagawa H M, Rassi T R E, Barbosa A C P, Silva A A. Corifollitropin alfa compared to daily rFSH or HP-HMG in GnRH antagonist controlled ovarian stimulation protocol for patients undergoing assisted reproduction. JBRA Assist Reprod. 2017;21(2):67-9. | Wrong comparator: Corifollitropin alfa vs rFSH/HP-hMG |
| Streda R, Mardesic T, Sobotka V, Tosner J. Long GnRH agonist vs. GnRH antagonist protocol in randomized controlled trial in unselected patients--hormonal and cycle characteristics--pilot study. Ceska gynekologie. 2009;74(2):75‐80-. | Language exclusion |
| Strehler E, Abt M, El Danasouri I, Gagsteiger F, Sterzik K. Impact of recombinant human follicle-stimulating hormone (rHFSH) and human menopausal gonadotrophins (HGM) on IVF/ICSI outcome. Human reproduction (Oxford). 1999;14:185-. | Abstract in meeting |
| Strehler E, Abt M, El-Danasouri I, De Santo M, Sterzik K. Impact of recombinant follicle-stimulating hormone and human menopausal gonadotropins on in vitro fertilization outcome. Fertility and Sterility. 2001;75(2):332-6. | Wrong comparator: not HP-hMG |
| Sun H, Hu Y, Wang B, Chen Q, Zhang N, Chen H, et al. [Effect of concentration of circulating luteinizing hormone in late-follicle phase on the outcome of in vitro fertilization for normogonadotrophic women]. Zhonghua Nan Ke Xue. 2004;10(12):912-5. | Language exclusion: Chinese |
| Tabata C, Fujiwara T, Sugawa M, Noma M, Onoue H, Kusumi M, et al. Comparison of FSH and hMG on ovarian stimulation outcome with a GnRH antagonist protocol in younger and advanced reproductive age women. Reproductive Medicine and Biology. 2015;14(1):5-9. | Wrong comparator: The FSH alone included rFSH or uFSH |
| Taheri F, Alemzadeh Mehrizi A, Khalili M A, Halvaei I. The influence of ovarian hyperstimulation drugs on morphometry and morphology of human oocytes in ICSI program. Taiwanese Journal of Obstetrics and Gynecology. 2018;57(2):205-10. | Wrong comparator: not HP-hMG |
| Tayyar A T, Kahraman S. Comparison between cycles of the same patients when using recombinant luteinizing hormone + recombinant follicle stimulating hormone (rFSH), human menopausal gonadotropin + rFSH and rFSH only. Archives of Medical Science. 2019;15(3):673-9. | Not RCT |
| Teissier M P, Chable H, Paulhac S, Aubard Y. Recombinant human follicle stimulating hormone versus human menopausal gonadotrophin induction: Effects in mature follicle endocrinology. Human Reproduction. 1999;14(9):2236-41. | Wrong outcomes |
| Thaller C. MEGASET study: Menotropin is just as good as recombinant FSH. New study data confirm the effectiveness and tolerance of MENOPUR® also in ICSI in GnRH antagonist cycles. Journal fur Gynakologische Endokrinologie. 2011;21(3):22-3. | Wrong study design |
| Toporcerová S, Hredzák R, Ostró A, Zdilová V, Potoceková D. [Influence of exogenous supplementation with luteinizing hormone during controlled ovarian hyperstimulation on the results of IVF cycle]. Ceska Gynekol. 2005;70(3):187-91. | Language exclusion |
| Torabizadeh A, Mirzaian S. Comparison of the IVF outcome between three methods of induction ovulation in PCOS patients. Iranian Journal of Obstetrics, Gynecology and Infertility. 2011;14(1). | Language exclusion |
| Trew G H, Brown A P, Gillard S, Blackmore S, Clewlow C, O'Donohoe P, et al. In vitro fertilisation with recombinant follicle stimulating hormone requires less IU usage compared with highly purified human menopausal gonadotrophin: Results from a European retrospective observational chart review. Reproductive Biology and Endocrinology. 2010;8. | Not RCT |
| Turkcapar F, Ozdener T, Batuoglu S. Influence of ovarian stimulation with HMG or recombinant FSH on OHSS prevention in PCO patients undergoing IVF. Fertility and Sterility. 2009;92 Suppl 1(3):S96-. | Abstract in meeting |
| Usha Rajinikanthan D B, Balasubramanyam S, Varma T. Comparison of in vitro fertilization/intracytoplasmic sperm injection outcomes in patients receiving recombinant human luteinizing hormone vs human menopausal gonadotropin supplementation. International Journal of Infertility and Fetal Medicine. 2016;7(3):77-81. | Wrong comparator: not HP-hMG |
| Vaiarelli A, Cimadomo D, Scarafia C, Innocenti F, Amendola M G, Fabozzi G, et al. Metaphase-II oocyte competence is unlinked to the gonadotrophins used for ovarian stimulation: a matched case–control study in women of advanced maternal age. Journal of Assisted Reproduction and Genetics. 2023;40(1):169-77. | Wrong study design |
| van Loenen A, Pirard C, Donnez J, Huirne J, Schats R, Lambalk C. Recombinant follicle stimulating hormone (R-FSH) versus recombinant luteinizing hormone (R-LH) and R-FSH treatment in combination with cetrorelix after oral contraceptive programming in IVF/ICSI; a feasibility study. Fertility and Sterility. 2002;78(3 Suppl 1):S46, Abstract no: O‐119-. | Abstract in meeting |
| van Wely M, Westergaard L G, Bossuyt P M, van der Veen F. Human menopausal gonadotropin and recombinant follicle-stimulating hormone for controlled ovarian hyperstimulation in assisted reproductive cycles. Fertil Steril. 2003;80(5):1121-2. | Wrong study design |
| Vega P G, De La Llata E S, Rodríguez A T, Haliscak R S, Reséndiz V A B, Montezco L A, et al. Prognostic values of oestradiol/oocyte rate in ICSI patients. Ginecologia y Obstetricia de Mexico. 2003;71(11):600-4. | Wrong comparator: not HP-hMG |
| Wang Y, Li L, Deng K, Liu J, Liu Y, Zou K, et al. Comparison of the combination of recombinant follicle-stimulating hormone and recombinant luteinizing hormone protocol versus human menopausal gonadotropin protocol in controlled ovarian stimulation: A systematic review and meta-analysis. Journal of Evidence-Based Medicine. 2020;13(3):215-26. | Wrong study design |
| Wang M, Huang R, Liang X, Mao Y, Shi W, Li Q. Recombinant LH supplementation improves cumulative live birth rates in the GnRH antagonist protocol: a multicenter retrospective study using a propensity score-matching analysis. Reproductive Biology and Endocrinology. 2022;20(1). | Not RCT |
| Wang L, Wang J, Hu Y Q, Liu J Y, Diao F Y. [Effects of time of recombinant luteinized hormone supplementation on pregnancy outcomes of women with advanced reproductive age during follicular-phase long protocol]. Zhonghua fu chan ke za zhi. 2022;57(10):758-66. | Language exclusion |
| Wang L, Wang J, Gao Y, Li M, Ni L, Liu J, et al. Effects of different exogenous LH activity drugs on pregnancy outcomes in patients with suboptimal ovarian response: a retrospective cohort study. Chinese Journal of Reproduction and Contraception. 2023;43(8):769-76. | Wrong design: retrospective cohort |
| Weghofer A, Munné S, Brannath W, Chen S, Barad D, Cohen J, et al. The impact of LH-containing gonadotropin stimulation on euploidy rates in preimplantation embryos: antagonist cycles. Fertility and Sterility. 2009;92(3):937-42. | Wrong comparator: the group of hMG included hMG and Hp-hMG |
| Weiss N S, Kostova E, Nahuis M, Mol B W J, van der Veen F, van Wely M. Gonadotrophins for ovulation induction in women with polycystic ovary syndrome. Cochrane Database of Systematic Reviews. 2019;2019(1). | Wrong study design: Systematic review |
| Westergaard L G, Erb K, Laursen S B, Rex S, Rasmussen P E. Human menopausal gonadotropin versus recombinant follicle-stimulating hormone in normogonadotropic women down-regulated with a gonadotropin-releasing hormone agonist who were undergoing in vitro fertilization and intracytoplasmic sperm injection: A prospective randomized study. Fertility and Sterility. 2001;76(3):543-9. | Wrong comparator: not HP-hMG |
| Westergaard L G, Erb K, Laursen S B, Rasmussen P E, Rex S, Westergaard C G, et al. Concentrations of gonadotrophins and steroids in pre-ovulatory follicular fluid and serum in relation to stimulation protocol and outcome of assisted reproduction treatment. Reproductive BioMedicine Online. 2004;8(5):516-23. | Wrong comparator: not HP-hMG |
| Wex-Wechowski J, Abou-Setta A M, Kildegaard Nielsen S, Kennedy R. HP-HMG versus rFSH in treatments combining fresh and frozen IVF cycles: Success rates and economic evaluation. Reproductive BioMedicine Online. 2010;21(2):166-78. | Wrong design: The data of efficacy were simulated. Economic study. |
| Witz C A, Doody K, Park J, Seifu Y, O'Brien K, Yankov V, et al. Highly purified human menotropin (HPHMG) versus recombinant follicle stimulating hormone (RFSH) in high responders undergoing in vitro fertilization (IVF): megaset-HR trial outcomes. Fertility and Sterility. 2017;108(3):e21‐e22-. | Abstract in meeting |
| Xia X, Shi Y, Geng L, Liu D, Hou Z, Lin H, et al. A cohort study of both human menopausal gonadotropin (HMG) and recombinant luteinizing hormone addition at early follicular stage in in vitro fertilization outcome: A STROBE-compliant study. Medicine (United States). 2019;98(19). | Not RCT |
| Yazici Yilmaz F, Görkemli H, Çolakoʇlu M C, Aktan M, Gezginc¸ K. The evaluation of recombinant LH supplementation in patients with suboptimal response to recombinant FSH undergoing IVF treatment with GnRH agonist down-regulation. Gynecological Endocrinology.2015;31(2):141-4. | Not RCT |
| Yenigul N N, Ozelçi R, Baser E, Dilbaz S, Aldemir O, Dilbaz B, et al. The value of LH supplementation in young women with diminished ovarian reserve treated with GnRH Antagonist Protocol for ovarian hyperstimulation in ICSI-cycles. Ginekol Pol. 2022. DOI: 10.5603/GP.a2021.0137 | Not RCT |
| Yetkinel S, Aytaç PÇ, Durdağ GD, Yağınç DA, Kılıçdağ EB, Şimşek E. Comparison of highly purified human menopausal gonadotropin and recombinant follicle stimulating hormone use in patients undergoing in vitro fertilization with progestin-primed ovarian stimulation protocol: a single center retrospective analysis. Arch Gynecol Obstet. 2024 Nov;310(5):2657-2662. | Wrong design |
| Younis J S, Izhaki I, Ben-Ami M. The effect of LH supplementation following GNRH antagonist administration in advanced reproductive ageing women undergoing IVF-ET: a prospective randomized controlled study. Fertility and Sterility. 2014;102(3):e23-. | Abstract in meeting |
| Younis J S, Izhaki I, Ben-Ami M. The effect of rLH supplementation to the GnRH-antagonist protocol on endocrine dynamics in the advanced reproductive age. Journal of Endocrinological Investigation. 2017;40(8):831‐839-. | Wrong outcomes |
| Yu Y, Zhang Q, Sun K, Xiu Y, Wang X, Wang K, Yan L. The therapeutic effects of rFSH versus uFSH/uHMG on ovarian stimulation in women undergoing assisted reproductive technology: a meta-analysis of randomized controlled trials. Arch Gynecol Obstet. 2024 Jun;309(6):2529-2555. | Wrong design: it is a systematic review |
| Zhu H, Wang Q, Huang W, Lu Y, Tang T. Recombinant luteinizing hormone (LH) supplementation improves controlled ovarian hyperstimulation outcome in women with low LH concentration during mid-and late-follicular phase. Latin American Journal of Pharmacy. 2021;40(6):1402-6. | Wrong design: retrospective cohort study |
| Ziebe S, Lundin K, Janssens R, Helmgaard L, Arce J C. Influence of ovarian stimulation with HP-hMG or recombinant FSH on embryo quality parameters in patients undergoing IVF. Human Reproduction. 2007;22(9):2404-13. | No additional data to included studies |

Supplementary Table 2 Characteristics of included studies. Comparison 1: adding LH activity to rFSH

| ID  Country | N | Age group | Women ovarian reserve | Pre-randomization | Intervention 1 | Intervention 2 | Downregulation | Funding  /COI | |
| --- | --- | --- | --- | --- | --- | --- | --- | --- | --- |
| HP-hMG vs rFSH | | | | | | | | | |
| Bosch 2008  NCT00669786  Spain | 280  ART | Mixed | NA | None | A daily dose of HP-hMG (225 IU/day) (Menopur, Ferring) for the first two days of stimulation. On day 3, depending on the ovarian response (E2 levels), the dosage could be adjusted up to a maximum of 300 IU/day or a minimum of 150 IU/day. | A daily dose of rFSH (225 IU/day) (Gonal F; Serono) for the first two days of stimulation. On day 3, depending on the ovarian response (E2 levels), the dosage could be adjusted up to a maximum of 300 IU/day or a minimum of 150 IU/day. | Antagonist  Cetrorelix (Cetrotide) | NR | |
| Bosch 2024  NCT02738580  Spain | 112  Donors | ≤35* | Normal  Criteria: AMH 10-30 pMol/l | None | A daily dose of HP-hMG (225 IU/day) (Meriofert KitVR , IBSA). | A daily dose of rFSH (225 IU/day) (Gonal-F, Merck). | Antagonist  Ganirelix acetate (Orgalutran) | Funding: Roche Diagnostics funded serum and follicular fluid hormone determinations.  COI: E.B. declares (see paper). The rest of authors report no conflicts. | |
| Chapon 2021  NCT02412904  Brazil | 168  ART | Mixed | Normal  Criteria: AMH 1-3 ng/ml; AFC >12 | None | A daily dose of HP-hMG (Menopur, Ferring), with dose between 150-300IU/day according to their AMH and AFC. | A daily dose of rFSH (Puregon, Organon), dose between 150-300IU/day according to their AMH and AFC. | Antagonist  Ganirelix acetate (Orgalutran) | Funding: No funding by pharmaceutical industry.  COI: NR | |
| Devroey 2012, Arce 2014  MEGASET  International | 749  ART | <35 | Normal  Criteria: Early follicular-phase total AFC >=10 for both ovaries combined. | None | A daily dose of HP-hMG (150 IU/day, with 1,200 IU FSH activity and 1,200 IU LH activity at 600 IU/mL) (Menopur; Ferring) for the first 5 days. From day 6 onwards, dosing could be changed by 75 IU per adjustment and not more frequently than every 4 days. | A daily dose of rFSH (150 IU/day) (follitropin beta; Puregon; MSD) for the first 5 days. From day 6 onwards, dosing could be changed by 75 IU per adjustment and not more frequently than every 4 days. | Antagonist  Ganirelix acetate (Orgalutran) | Funding: Ferring Pharmaceuticals, for conduct of statistical analysis. | |
| EISG 2002, Platteau 2004  EISG  International | 781  ART | ≤35* | NA | None | A daily dose of HP-hMG (225 IU/day) for 5 days. On day 6 (visit 4), depending on the ovarian response, the dosage could be adjusted up to a maximum of 450 IU/day. | Daily dose of rFSH (225 IU/day) for 5 days. On day 6 (visit 4), depending on the ovarian response, the dosage could be adjusted up to a maximum of 450 IU/day. | Agonist (long) | Funding: Ferring Pharmaceuticals.  COI: NR | |
| Hompes 2008  Netherlands | 629  ART | Mixed | NA | None | A daily dose of HP-hMG (150 IU/day) (Menopur), duration: max 14 days. Daily dose could be increased or decreased based on response (number of follicles) . | A daily dose of rFSH (150 IU/day) (Gonal-F, Serono; or Puregon, Organon), duration: max 14 days. (day start NR) Daily dose could be increased or decreased based on response (number of follicles). | Agonist  Leuprolide acetate / Triptorelin | NR | |
| Kilani 2003  Italy | 100  ART | <35 | NA | None | A daily dose of HP-hMG (150 IU/day) (Menopur; Ferring); continued until at least three ovarian follicles >o= 18mm AND E2 >600 pg/ml OR for 14 days. Post 14 days increments of dose were allowed (225 IU/day on days 15±17 and 300 IU/day on days 18±20). | A daily dose of rFSH (150 IU/day) (Gonal-F; Serono); continued until at least three ovarian follicles >o= 18mm AND E2 >600 pg/ml OR for 14 days. Post 14 days increments of dose were allowed (225 IU/day on days 15±17 and 300 IU/day on days 18±20). | Agonist (long)  Triptorelin (Decapeptyl) | Funding: Ferring Pharmaceuticals.  COI: NR | |
| Melo 2010  NCT00829075  Spain | 1028  Donors | Mixed | NA | None | A daily dose of HP-hMG (225 IU/day) (Menopur; Ferring) for 5 days. From day 5, dosage adjusted based on ovarian response (E2 levels). | A daily dose of rFSH (225 IU/day) (Gonal-F; Serono) for 5 days. From day 5, dosage adjusted based on ovarian response (E2 levels). | Agonist  Leuprolide acetate (Procrin) | Funding: NR COI: The authors report no conflict of interests. | |
| Miller 2013  US | 173  ART | Mixed | NA | None | A daily dose of HP-hMG (225 IU/day) (Menopur, Ferring) for minimum 5 days. On day 5, dose could be increased or decreased by <=150 IU/day, up to a maximum of 450 IU/day, based on ovarian response. | A daily dose of rFSH (225 UI/day) (Follistim, Merck) for a minimum of 5 days. On day 5, dose could be increased or decreased by <=150 IU/day, up to a maximum of 450 IU/day, based on ovarian response. | Antagonist  Ganirelix acetate | Funding: Ferring Pharmaceuticals.  COI: Authors declare (see paper). | |
| Nyboe Andersen 2006, Anckaert 2012, Arce 2014  MERIT  International | 731  ART | <35 and ≥35 | Poor and high  Criteria: AMH quartiles | None | A daily dose of HP-hMG (225 IU/day) (Menopur, Ferring) for 5 days. After 5 days, dose could be adjusted according to follicular response by 75 IU per adjustment (up to a maximum of 450 IU/day) and not more frequent than every 4 days. Duration: maximum of 20 days | A daily dose of rFSH (225 IU/day) (Gonal-F, Serono) for 5 days. After 5 days, dose could be adjusted according to follicular response by 75 IU per adjustment (up to a maximum of 450 IU/day) and not more frequent than every 4 days.  Duration: maximum of 20 days | Agonist (long)  Triptorelin (Decapeptyl) | Funding: NR COI: Authors declare (see paper). | |
| Witz 2020  MEGASET-HR NCT02554279  International | 620  ART | <35 | High  Criteria: AMH >= 5 ng/mL | None | A daily dose of HP-hMG (150 IU/day) (Menopur, Ferring), until day 5. From day 6, doses could be adjusted by 75 IU/day to max 300 IU/day based on ovarian response. Duration of treatment: maximum 20 days | A daily dose of rFSH (150 IU/day) (Gonal-f; EMD-Serono) until day 5. From day 6, doses could be adjusted by 75 IU/day to max 300 IU/day, based on ovarian response. Duration of treatment: max 20 days. | Antagonist  Ganirelix acetate (Orgalutran) | Funding: Ferring Pharmaceuticals.  COI: NR | |
| Yahyaei 2023  NCT03876145  Iran | 120  ART | ≤35* | High  Criteria: 100% PCOS | None | Mild-FSH received rFSH (150 IU/day) (Gonal-F, Merck Serono) from day 3 of the menstrual cycle. | Mild-HMG received HP-hMG (150 IU/day) (Merional, IBSA) from day 3 of the menstrual cycle. | Antagonist  Cetrorelix (Cetrotide) | Funding: No financial support has been granted.  COI: The authors report no conflict of interest. | |
| Ye 2012  China | 127  ART | ≥35 | NA | None | A daily fixed dose of HP-hMG (225 IU/day) (Menopur, Ferring) for the first 5 days. From day 5, dosage of HP-hMG could be adjusted according to ovarian response. | A daily fixed dose of rFSH (225 IU/day) (Gonal-F, Serono) for 5 days. From the 5th day, dosage of rFSH could be adjusted according to ovarian response. | Antagonist  Triptorelin | Funding: NR  COI: The authors report no conflict of interest. | |
| * These studies included participants up to 36, 37 and 38 years old, which were considered in the younger age group | | | | | | | | | |
| HP-hMG + rFSH vs rFSH | | | | | | | | |  |
| Drakopoulos 2017  NCT01816321 COMPORT  International | 152  ART | Mixed | Poor  Criteria: Bologna criteria | None | A single dose of 150 μg corifollitropin alfa (stimulation day 1); on stimulation Day 8 onwards, daily dose of HP-HMG (300 IU/day). | A daily dose of rFSH (300 IU/day) . | Antagonist  Ganirelix acetate | Funding: No external funding.  COI: C.B., P.H., H.T., N.P.P. declare (see paper). The rest of the authors report no conflict of interest. |  |
| Melo 2010  NCT00829075  Spain | 1028  Donors | Mixed | NA | None | A daily dose of rFSH (150 IU/day) (Gonal; Serono) and HP-hMG (75 IU/day) (Menopur; Ferring), for 5 days. From day 5, dosage could be adjusted based on ovarian response (E2 levels). | A daily dose of rFSH (225 IU/day) (Gonal-F; Serono) for 5 days. From day 5, dosage adjusted based on ovarian response (E2 levels). | Agonist  Leuprolide acetate (Procrin) | Funding: NR COI: The Authors report no conflict of interests. |  |
| Qiu 2023  ChiCTR2100043040  China | 172  ART | ≥35 | Poor  Criteria: AFC <5 or AMH <1.2 | None | A daily dose of HP-hMG (75 IU/day) (H10940097, Menotropin, Lizhu Group) and a daily dose of rFSH (225-300 IU/day). The starting doses of FSH ranged from 225 to 300 IU based on age, ovarian reserve, BMI, hormone levels, and previous response to fertility treatments. Dose could be adjusted according to the ovarian response. | A daily dose of rFSH (225 - 300IU/day). The starting doses of FSH ranged from 225 to 300 IU based on age, ovarian reserve, BMI, hormone levels, and previous response to fertility treatments. Dose could be adjusted according to the ovarian response. | Agonist and antagonist (long)  Triptorelin, Cetrorelix | Funding: Key R & D projects of Sichuan Provincial Department of Science and Technology under Grant 2021YFS0243.  COI:NR |  |
| Shu 2019  ChiCTR-TRC-14004552  China | 610  ART | ≤35* | Normal or high  Criteria: AFC >6 | None | A fixed daily dose of HP-HMG (75 IU/day) (Menopur, Ferring) and a daily dose of rFSH (75-150 IU/day) (Gonal-F, Merck Serono). Dose of rFSH 75IU/day for those weighing ≤60 kg, and 150 IU rFSH for those >60 kg. | A daily dose of rFSH (150-225 IU/day) (Gonal-F, Merck Serono). Dosage of rFSH was 150 IU/day for those weighing ≤60 kg, or 225 IU/day for those >60 kg. | Agonist  Triptorelin (Decapeptyl) | Funding: National 973 program, the National Health and Family Planning Commission of China, and the Jiangsu Province Special Program of Medical Science.  COI: The authors report no conflict of interest. |  |
| Sohrabvand 2010  Iran | 64  ART | <35 | NA | A fixed daily dose of rFSH (150 IU/day) (Gonal-F, Serono) for 6 days. | A daily dose of HP-hMG (75 IU/day) (Merional, IBSA) and one additional dose of rFSH (75 IU/day) (Gonal-F, Serono) was started on day seven. Dose of HP-hMG was adjusted with 1-2 additional ampoules (75-150 IU), according to ovarian response. | Standing rFSH treatment, with dosage adjustment. From day 7, the dose was adjusted with 1-2 additional ampoules (75-150 IU) of Gonal-F, according to ovarian response. | Agonist  Buserelin | Funding: NR COI: The authors report no conflict of interest. |  |
| * These studies included participants up to 36, 37 and 38 years old, which were considered in the younger age group | | | | | | | | |  |
| rLH + rFSH vs rFSH | | | | | | | | |  |
| Balasch 2001  Spain | 30  ART | Mixed | NA | None | A daily dose of rFSH (Gonal F; Ares-Serono) and a fixed daily dose of rLH (75 IU/day) (Luveris; Ares-Serono). Dosage of rFSH followed a step-down regimen of 450 IU on day 1, 300 IU on day 2, and 150 IU on days 3 to 5. From day 6 onward, dosage of rFSH was according to ovarian response (E2 levels). | A daily dose of rFSH (Gonal F; Ares-Serono), according to a step-down regimen of 450 IU on day 1, 300 IU on day 2, and 150 IU on days 3 to 5. From day 6 onward, dosage of rFSH according to ovarian response (E2 levels). | Agonist  Leuprolide acetate (Procrin) | Funding: rFSH and rLH provided by Ares-Serono.  COI: NR. |  |
| Barrenetxea 2008  Spain | 84  ART | ≥35 | NA | None | A daily dose of rFSH (375 IU/day) (Gonal F, Serono Europe). On day 7 of ovarian stimulation, a fixed daily dose of rLH (150 IU/day) (Luveris; Serono) was added. | A daily dose of rFSH (375 IU/day) (Gonal F, Serono). | Agonist  Leuprolide acetate (Procrin) | NR |  |
| Berkkanoglu 2007  Turkey | 97  ART | Mixed | Poor  Criteria: Fewer than 12 antral follicles | A daily dose of rFSH (600 IU/day) (Gonal-F, Serono; Puregon, Organon) for 7 days | A daily dose of rLH (75 IU/day) (Luveris, Serono) and a daily dose of rFSH (600 IU/day) (Gonal-F, Serono; Puregon, Organon). | A daily dose of rFSH (600 IU/day) (Gonal-F, Serono; Puregon, Organon). | Agonist  Leuprolide acetate (Lucrin) | Funding: NR  COI: NR |  |
| Bosch 2011  Spain | 720  ART | <35 and ≥35 | NA | None | A daily dose of rFSH (Gonal-f; Merck Serono) plus rLH (Luveris; Merck Serono).  For age <=35y: A daily dose of rFSH (150 IU/d) and a fixed daily dose of rLH (75 IU/day) for the first 5 days of stimulation.  For age 36-39: A daily dose of rFSH (300 IU/d) and a fixed daily dose of rLH (75 IU/day) for the first 5 days of stimulation.  Depending on the ovarian response, the dosage of rFSH could be adjusted. | A daily dose of rFSH (Gonal-f; Merck Serono,) plus rLH (Luveris; Merck Serono).  For age <=35y: A daily dose of rFSH (225 IU/d) SC for the first 5 days of stimulation. For age 36-39: A daily dose of rFSH (300 IU/d) SC for the first 5 days of stimulation. Depending on the ovarian response, the dosage of rFSH could be adjusted. | Antagonist  Cetrorelix (Cetrotide) | Funding: NR  COI: The Authors report no conflict of interests. |  |
| Caserta 2011  Italy | 999  ART | Mixed | NA | None | A daily dose of rFSH (150 UI/day). At day 7 of stimulation, a fixed daily dose of rLH (75 IU/day) was added and the dose of rFSH adapted according to ovarian response. | A daily dose of rFSH (150 IU/day) (Gonal F, Serono). At the 7th day of stimulation, the dose of rFSH was adapted according to ovarian response. | Agonist  Triptorelin | Funding: NR  COI: The authors report no conflicts of interest. |  |
| Cedrin 2004  France | 218  ART | Mixed | NA | A fixed daily dose of rFSH (150-300 IU/day) (Gonal F, Serono). Starting dose based on age, BMI and ovarian responsiveness in previous cycles. | A fixed daily dose of rLH (75 IU/day) (Luveris; Serono) was added on day 6 to standing rFSH treatment, and up to hCG administration. Up to starting antagonist administration, dosage of RFSH could be individually adjusted according to response. | Standing rFSH treatment was continued from day 6 up to hCG administration. Up to starting antagonist administration, dosage of rFSH could be individually adjusted according to response. | Antagonist  Cetrolerix (Cetrotide) | Funding: NR  COI: NR |  |
| DePlacido 2005  Italy | 130  ART | ≤35* | NA | A daily dose of 225IU of rFSH (Gonal-F; Serono Pharma, Rome, Italy). On day 5, rFSH reduced by 75 IU if E2 levels > 180 pg/ml. | On the 8th day of stimulation, daily dose of rLH (150 IU/day) added to standing gonadotropin treatment. | On the 8th day of stimulation, an increase of 150 IU in the daily dose of rFSH (step-up protocol). | Agonist (long)  Triptorelin (Decapeptyl) | Funding: Ministero dell’Istruzione, dell’Universita` e della Ricerca (PRIN annualita` 2003 prot. 2003065093_002).  COI: NR |  |
| Evangelio 2011  Spain | 90  ART | ≥35 | NA | None | A fixed daily dose of rLH (dose 1:2 to rFSH) (Luveris, Merck Serono) and a fixed daily dose of rFSH (dose based on expected response) (Gonal F / Puregon) in 1:2 or 1:3 rate until hCG trigger. | A fixed daily dose of rFSH (dose based on expected response) (Gonal F / Puregon) until hCG trigger. | Antagonist  Ovitrelle (Serono) | Funding: NR  COI: NR |  |
| Fábregues 2006  Spain | 120  ART | ≥35 | NA | None | A fixed daily dose of rLH (dose NR) (Luveris; Serono) starting on day 6, and a daily dose of rFSH(Gonal-F; Serono) starting from day 1 until hCG injection. Dosage of rFSH was 450 IU/day (Day 1), 300 IU/day (day 2), 150 IU/day (day 3 to 5). From day 5 onward, dosage could be modified individually based on ovarian response. | A daily dose of rFSH (Gonal-F; Serono) until hCG injection. Dosage of rFSH was 450 IU/day (Day 1), 300 IU/day (day 2), 150 IU/day (day 3 to 5). From day 5 onward, dosage could be modified individually based on ovarian response. | Agonist Triptorelin (Decapeptyl) | Funding: Instituto de Salud Carlos III (RCMN C03/08) and the Comissionat per a Universitat i Recerca-Generalitat de Catalunya (2001SGR 00372 and 2005SGR 00573).  COI: NR |  |
| Fábregues 2011  Spain | 187  ART | ≥35 | NA | None | A fixed daily dose of rLH (75 IU /day or 37.5 IU/day) (Luveris, Serono) started on day 6, and a daily dose of rFSH (Gonal-F, Merck-Serono), until administration of hCG day. Dose of rFSH was 450 IU (Day 1), 300 IU (Day 2), 150 IU (Day 3-4). From day 5 onwards, rFSH dose could be modified individually according to ovarian response. | A daily dose of rFSH (Gonal-F, Merck-Serono), until administration of hCG day. Dose of rFSH was 450 IU (Day 1), 300 IU (Day 2), 150 IU (Day 3-4). From day 5 onwards, dose could be modified individually according to ovarian response. | Agonist Triptorelin (Decapeptyl) | Funding: Agència de Gestió d´Ajuts Universitaris i de Recerca-Generalitat de Catalunya (2009SGR 1099).  COI: NR |  |
| Fernández Ramírez 2006  Spain | 34  ART | ≤35* | NA | None | A daily dose of rFSH (300-450 IU/day) (Gonal F, Serono) and a daily dose of rLH (150 IU/day) (Luveris, Serono). | A daily dose of rFSH (Gonal F, Serono). The dose was later adjusted based on ovarian response (E2 levels). Up to complete ovarian stimulation. | Antagonist  Cetrorelix | Funding: Serono.  COI: NR |  |
| Ferrareti 2004  Italy | 108  ART | ≤35* | Poor  Criteria: No response to initial gonadotropin | A daily dose of rFSH (150-225 IU/day) (Gonal F, Serono) depending on age for 7-10 days. | Increase of rFSH dose (max 450 IU/day), and addition of a daily dose of rLH (75–150 IU/day) (Luveris, Serono). | Increase of rFSH dose (max 450 IU/day). | Agonist  NR | NR |  |
| Ferraretti 2014  Italy | 43  ART | Mixed | Poor  Criteria: No response to at least two previous cycles with maximal FSH/HMG stimulation. | None | A daily dose of rLH (150 IU/day) for 4 days, plus rFSH (400 IU/day) starting at day 5 in agonist downregulation protocol. | A daily dose of rFSH (400 IU/day). | Agonist or antagonist | Funding: NR COI: The authors report no conflict of interests. |  |
| Griesinger 2005  Germany | 127  ART | Mixed | NA | None | A daily dose of rFSH (150 IU/day) (Gonal-F; Serono) and a daily dose of rLH (75 IU/day) (Luveris; Serono). From day 6 onwards, the rFSH dosage could be increased (up to 225-300 IU/day) according to ovarian response (E2 levels). In case of a dose increment to 300 IU rFSH, the rLH dose was concomitantly adjusted to 150 IU/day. | A daily dose of rFSH (150 IU/day) (Gonal-F; Serono). From day 6 onwards, the rFSH dosage could be increased (up to 225-300 IU/day) according to ovarian response (E2 levels). | Antagonist  Cetrorelix (Cetrotide) | NR |  |
| Gutman 2009  Israel | 20  ART | Mixed | NA | A daily dose of rFSH (150–225 IU/day) (Gonal F; Serono), until mean follicles diameter >14mm. | A daily dose of rLH (75 IU/day) (Luveris,Serono). | Placebo | Agonist  Buserelin | Funding: NR COI: The authors report no conflict of interests. |  |
| Humaidan 2004  Denmark | 231  ART | <35 and ≥35 | NA | A daily dose of rFSH (Gonal F, Serono; or Puregon, Organon) based on Age, baseline FSH, BMI and ovarian volume. On day 8, the dose of rFSH could be adjusted based on ovarian response. | A daily dose of rLH (Luveris, Serono) was added to standing rFSH treatment. Dose of rFSH and rLH in a ratio of 2:1. | No supplementation to standing rFSH treatment. | Agonist  Buserelin (Suprefact) | NR |  |
| Humaidan 2017  ESPART NCT02047227  Denmark | 939  ART | Mixed | Poor  Criteria: Modified Bologna criteria | None | A daily dose of rLH (150 IU/day) (Pergoveris) and rFSH (300 IU/day) (Gonal-F), at a fixed ratio of 1:1, with the dose fixed for the first 4 days of COS. | A daily dose of rFSH (300 IU/day) (Gonal-F), with the dose fixed for the first 4 days of COS. | Agonist  Triptorelin acetate | Funding: Merck KGaA.  COI: Authors declare (see paper). |  |
| König 2013  ISRCTN10841210  Netherlands | 253  ART | ≥35 | NA | A fixed daily dose of r-FSH (225 IU/day) (Gonal-F; Merck Serono). | On day 6 of stimulation, a fixed daily dose of rLH (150 UI/day) (Luveris, Merck Serono) was added to standing rFSH treatment. No dose adjustment was allowed. | Standing treatment with rFSH was continued up to hCG administration. No dose adjustment was allowed. | Antagonist  Cetrorelix (Cetrotide) | Funding: Merck Serono donated the r-LH (Luveris).  COI: The authors report no conflict of interest. |  |
| Kovacs 2010  Hungary | 50  ART | Mixed | NA | None | A fixed daily dose of rLH (75 IU/day) for 4 days. A daily dose of rFSH (150 IU/day) up for 5 days. After day 5 of rFSH, dose of rFSH could be adjusted based on ovarian response. | A fixed daily dose of rFSH (150 IU/day) for the first 5 days. After day 5, dose of rFSH could be adjusted based on ovarian response. | Agonist (long)  Buserelin (Suprefact) | NR |  |
| Lahoud 2017  ACTRN12610000064000  Australia | 100  ART | Mixed | NA | A daily dose of rFSH (dosage individualized) (Gonal F, Merck Serono; or Puregon, Merck Sharp & Dohme) at a dosage based on IVF Australia guidelines. | A daily dose of rLH (75 IU/day) (Luveris, Merck Serono) and a daily dose of rFSH (dose NR) (Gonal F / Puregon) until hCG trigger. | A daily dose of rFSH (dose NR) (Gonal F, Merck Serono) until rHCG trigger. | Agonist  Leuprolide (Lucrin) or Nafarelin (Synarel) | Funding: NR  COI: NR |  |
| Levi-Setti 2006  Italy | 40  ART | ≤35* | NA | A daily dose of r-FSH (225 IU/day) (Gonal-F; Serono), until mean follicles diameter >14mm. | A daily dose of rLH (75 IU/day) (Luveris; Serono) and a daily dose of rFSH (150 UI/day) (Gonal-F, Serono). | Standing treatment with rFSH was continued up to hCG administration | Antagonist  Cetrorelix (Cetrotide) | NR |  |
| Lisi 2012  Italy | 150  ART | Mixed | NA | None | A daily dose of rLH (75 IU/day) for 4 days, and a fixed daily dose of rFSH (150 IU/day) for 6 days. On the 7th day the dose of rFSH was adjusted according to the individual response. | A daily dose of rFSH (150 IU/day) (Gonal F, Merck-Serono) for 6 days. On the 7th day, the dose of rFSH was adjusted according to individual response. | Agonist  Triptorelin | Funding: NR  COI: The authors report no conflict of interest. |  |
| Marrs 2004  US | 431  ART | <35 and ≥35 | NA | None | A fixed daily dose of rLH (150 IU/day) (Luveris; Serono Laboratories) on stimulation day 6, and a daily dose of rFSH (225 IU/day) (Gonal-F; Serono Laboratories). After day 5, dosage of rFSH could be increased by 75-150 IU/day every 2-3 days | A daily dose of rFSH (225 IU/day) (Gonal-F; Serono Laboratories) for 5 days. After day 5, dosage of rFSH could be increased by 75-150 IU/day every 2-3 days | Agonist  Leuprolide acetate (Lupron) | Funding: NR  COI: NR |  |
| Matorras 2009  EUDRACT 2004-001503-36  Spain | 131  ART | ≥35 | NA | A fixed daily dose of rFSH (300–450 IU/day) (Gonal-F, Merck Serono). | A daily dose of rLH (150 IU/day) (Luveris, Merck Serono) was added to standing treatment with rFSH on day 6 of stimulation. Dose of rFSH was adjusted if needed after day 6 of stimulation. | Standing treatment with rFSH was continued. Dose of rFSH was adjusted if needed after day 6 of stimulation. | Agonist  Triptorelin (Decapeptyl) | Funding: Merck. Funding and editorial assistance.  COI: NR |  |
| Musters 2012  Netherlands | 244  ART | Mixed | Poor  Criteria: Age 35–41 or <35 + FSH > 12 IU/ml + AFC ≤ 5 | None | A daily dose of rFSH (150-450 IU/day) (Gonal-F, Merck Serono) and rLH (75-225 IU/day) (Luveris, Merck Serono). Starting doses based on AFC levels. After day 7 doses were adjusted according to ovarian response, up to a maximum of 450 IU rFSH and 225 IU rLH and keeping the ratio 2:1. | A daily dose of rFSH (150-450 IU/day) (Gonal-F, Merck Serono). Starting doses based on AFC levels. After day 7 doses were adjusted according to ovarian response, up to a maximum of 450 IU mg/day. | Agonist (long)  Triptorelin (Decapeptyl) | Funding: Merck Serono donated the rLH (Luveris) and the HCG (Ovitrelle) COI:NR |  |
| NyboeAndersen 2008  International | 526  ART | <35 and ≥35 | NA | None | A fixed daily dose of rFSH (150–225 IU/day) (Gonal-F, Serono) for six days, and a fixed daily dose of rLH (75-150 IU/day) (Luveris, Serono) started on day 6 of stimulation. Dose of rFSH according to age. Dose of rLH according to age. The rFSH dose was fixed for the first six days, after which the dose could be individualized. | A fixed daily dose of rFSH (150–225 IU/day) (Gonal-F, Serono) for 6 days. Dose according to age. After 6 days, the dose could be individualized. | Agonist  Nafarelin (Synarela) | Funding: Serono Nordic provided Luveris (rLH), funded the central measurements of serum LH, and conducted the statistical analysis.  COI: NR |  |
| Pezzuto 2010  Italy | 80  ART | Mixed | NA | A daily dose of rFSH (225–300 UI/day) (Puregon, Organon) . Dosing by age. | A daily dose of rLH (75 UI/day) (Luveris, Serono) was added to standing treatment with rFSH. Doses of rFSH could be adjusted based on response. | Standing treatment with rFSH continued. Doses of rFSH could be adjusted based on response. | Agonist (long)  Leuprolerin acetate (Enantone) | Funding: NR  COI: The authors report no conflicts of interest. |  |
| Rahman 2017  NCT03204253  Italy | 66  ART | Mixed | NA | None | A dose of rLH (75 IU /day) and a dose of rFSH (dose not reported). Addition of rLH (150 IU) about 12 hours before triggering ovulation with hCG. | A daily dose of rFSH (dose NR). | Antagonist  NR | Funding: NR. COI: The authors report no conflict of interests. |  |
| Razi 2014  Iran | 40  ART | <35 | NA | A daily dose of rFSH (150-225 IU/day) (Gonal-F, Serono), until mean follicles diameter >14mm. | A daily dose of rLH (75 IU/day) (Luveris, Serono) was added to standing rFSH treatment, for a maximum of 10 days. | Standing treatment with rFSH was continued for a maximum of 10 days. | Agonist (long)  Buserelin (Cinnafact) | Funding: Yazd Research and Clinical Center for Infertility.  COI:NR |  |
| Ruvolo 2007  Italy | 42  ART | Mixed | NA | A fixed daily dose of rFSH (225 IU/day) (Gonal-F, Serono) until day 6. From day 6, the dose of rFSH adjusted based on ovarian response (E2 levels). | A daily dose of rLH (75–150 IU/day) (Luveris, Serono) was added to standing rFSH treatment on day 8. | Standing treatment with rFSH was continued. | Agonist  Buserelin | Funding: Italian Ministero Istruzione Università Ricerca.  COI:NR |  |
| Tarlatzis 2006  International | 114  ART | ≤35* | NA | A daily dose of rFSH (150 IU/day) (Gonal-F, Serono) for 5 days. After 5 days dose adjusted to a max 450 IU/day according to ovarian response. | A daily dose of rLH (75 IU/day) (Luveris, Serono) was added to standing rFSH treatment. Duration for a maximum of 10 days. | Placebo was added to standing rFSH treatment. Duration for a maximum of 10 days. | Agonist (long)  Buserelin (Suprefact) | Funding: Serono (grant number GF9318).  COI: NR |  |
| Vuong 2015  NCT02244866  Vietnam | 240  ART | ≥35 | NA | None | A daily dose of rFSH (150-300 IU/day) (Gonal-F, Merck Serono) for 5 days. Dose based on AFC. On day 6, participants were switched to a daily dose of a combination of rLH + r FSH (150/75 IU/day) (Pergoveris, Merck Serono). Dosage of rFSH could be adapted based on ovarian response. | A daily dose of rFSH (150-300 IU/day) (Gonal-F, Merck Serono) for 5 days. Dose based on AFC. Dosage of rFSH could be adapted based on ovarian response. | Antagonist  Cetrorelix (Cetrotide) | Funding: Research Center for Genetics and Reproductive Health, Vietnam National University.  COI: The authors report no conflicts of interest. |  |
| Younis 2016  NCT01016210  Israel | 63  ART | Mixed | Poor  Criteria: Age 35-44 or poor ovarian response Bologna criteria | None | A daily dose of rFSH (300 IU/day) (Gonal-F, Merck Serono) and a daily dose of rLH (50 IU/day) (Luveris, Merck Serono) added when the leading follicle was 15mm diameter. Dosage to keep the ratio of FSH:LH to 2:1. | A daily dose of rFSH (300 IU/day) (Gonal-F, Merck Serono). | Antagonist  Cetrorelix (Cetrotide) | Funding: Merck-Serono Israel provided Luveris. COI: The authors report no conflict of interest. |  |

Comparison 2: Different sources of LH activity

| ID  Country | Sample | Age group | Women Ovarian reserve | | Pre-randomization | | Intervention 1 | Intervention 2 | Downregulation | Funding/COI |
| --- | --- | --- | --- | --- | --- | --- | --- | --- | --- | --- |
| HP-hMG vs rLH + rFSH | | | | | | | | | | |
| Carone 2012  NCT01623570  Italy | 35  ART | ≤35* | NA | | None | Daily dose of HP-hMG (150 IU/day, as 150 IU FSH + 150 IU LH-like activity) (Ferring), for a maximum of 16 days. | | A fixed daily dose of rFSH (150IU/day) and rLH (75IU/day) (Merck Serono) for a maximum of 16 days. | NR  NR | NR |
| Pacchiarotti 2010  Italy | 122  ART | NR | NA | | None | A daily dose of HP-hMG (225 IU/day) (Meropur, Ferring). | | A daily dose of rFSH + rLH (225 IU/day) (Pergoveris, Serono). | Agonist  Triptorelin | Funding: NR COI: The authors report no conflict of interests. |
| HP-hMG + rFSH vs rLH + rFSH | | | | | | | | | | |
| Gómez-Palomares 2005  Spain | 94  ART | ≥35 | NA |  | None | A daily i.m. dose of rFSH (225 IU/day) (Gonal F; Serono) and HP-hMG HMG (HMG-Lepori; Pharma Lepori) (75 IU/day rFSH and 75 IU/day of rLH) for 5 days. On day 6 of stimulation, only daily dose of rFSH in a step-down protocol. | | A daily Dose of rFSH (300 IU/day) (Gonal F; Serono) and rLH (75 IU/day) (Luveris; Serono) for 5 days. On day 6 of stimulation, only daily dose of rFSH in a step-down protocol. | Agonist  Leuprolide acetate (Procrin) | NR |
| Shahrokh 2017  IRCT2016092720351N3  Iran | 140  ART | <35 | NA | | None | A daily dose of rFSH 150-225 IU/day) (Gonal-F, Serono), and a daily dose of HP-hMG (150-225 IU/day) (Merional) started on day 6 of stimulation. Dosage of rFSH and HP-hMG was based on age and ovarian response. | | A daily dose of rFSH 150-225 IU/day) (Gonal-F, Serono), and a daily dose of rLH (75 IU/day) (Luveris, Serono) started on day 6 of stimulation | Agonist  Triptorelin (Decapeptyl) + Buserelin | Funding: NR  COI: The authors report no conflict of interest. |
| * These studies included participants up to 36, 37 and 38 years old, which were considered in the younger age group | | | | | | | | | | |

**Comparison 3: Other comparisons**

| ID  Country | Sample | Age group | Ovarian reserve | Pre-randomization | Intervention 1 | Intervention 2 | Downregulation | Funding/COI |
| --- | --- | --- | --- | --- | --- | --- | --- | --- |
| rLH + rFSH vs rLH + rFSH | | | | | | | | |
| DePlacido 2004  Italy | 46  ART | ≤35* | NA | A daily dose of rFSH (150–300 IU/day) (Gonal-F; Serono), according to patients age and BMI. On day 5, dose was reduced by 75 IU if E2 levels > 160 pg/ml. | On the 8th day of stimulation, a daily rLH dose (75 IU/day) added to standing gonadotropin treatment. | On the 8th day of stimulation, a daily rLH dose (150 IU/day) added to standing gonadotropin treatment. | Agonist (long)  Triptorelin (Decapeptyl) | Funding: Progetti autonomamente proposti, L.R. 41/94, 2000’.  COI: NR |
| Fábregues 2011  Spain | 187  ART | ≥35 | NA | None | A fixed daily dose of rLH (75 IU/day) (Luveris, Serono) started on day 6, and a daily dose of rFSH (Gonal-F, Merck-Serono), until administration of hCG day. Dose of rFSH was 450 IU (Day 1), 300 IU (Day 2), 150 IU (Day 3-4). From day 5 onwards, rFSH dose could be modified individually according to ovarian response. | A fixed daily dose of rLH (37.5 IU/day) (Luveris, Serono) started on day 6, and a daily dose of rFSH (Gonal-F, Merck-Serono), until administration of hCG day. Dose of rFSH was 450 IU (Day 1), 300 IU (Day 2), 150 IU (Day 3-4). From day 5 onwards, rFSH dose could be modified individually according to ovarian response. | Agonist Triptorelin (Decapeptyl) | Funding: Agència de Gestió d´Ajuts Universitaris i de Recerca-Generalitat de Catalunya (2009SGR 1099).  COI: NR |
| rFSH + HP-hMG vs HP-hMG | | | | | | | | |
| Melo 2010  NCT00829075  Spain | 1028  Donors | Mixed | NA | None | A daily dose of rFSH (150 IU/day) (Gonal; Serono) and HP-hMG (75 IU/day) (Menopur; Ferring), for 5 days. From day 5, dosage could be adjusted based on ovarian response (E2 levels). | A daily dose of HP-hMG (225 IU/day) (Menopur; Ferring) for 5 days. From day 5, dosage adjusted based on ovarian response (E2 levels). | Agonist  Leuprolide acetate (Procrin) | Funding: NR COI: The authors report no conflict of interests. |
| Taronger 2018  Spain | 234  ART | Mixed | Poor  Criteria: Abnormal ovarian reserve test, AMH < 8 pmol/L, or AFC < 7 | None | A single injection of Corifollitropin alfa (150 ug) (Elonva) on cycle day 2-3. On day 8, a daily dose of HP-hMG (300 IU/day) (Menopur) was added. | A daily dose of HP-hMG (300 IU/day) (Menopur) at continuous daily dose. | Antagonist  Ganirelix acetate | Funding: Health Research Institute La Fe (IIS La Fe) and Spanish Clinical Research Network, SCReN-IIS La Fe, PT17/0017/0035.  COI: The authors report no conflicts of interest. |

**Supplementary Table 3** **Summary of subgroup analysis Comparison 1: Gonadotropin with activity LH vs rFSH**

|  | **Studies** | **Participants** | **Risk Ratio**  **(M-H, Random, 95% CI)** | **Test for subgroup differences**  ***p*** |
| --- | --- | --- | --- | --- |
| Live birth |  |  |  |  |
| **Ovarian reserve** | 6 | 2221 | 1.19 [0.96, 1.47] |  |
| Poor reserve_HP-hMG + rFSH | 1 | 152 | 0.97 [0.43, 2.20] | 0.86 |
| Poor reserve_rLH+rFSH | 2 | 1047 | 1.29 [0.59, 2.80] |  |
| High reserve_HP-hMG | 3 | 1022 | 1.22 [0.93, 1.59] |  |
| **Age≤35 years** | 5 | 1654 | 1.09 [0.87, 1.36] |  |
| HP-hMG | 2 | 1368 | 1.08 [0.94, 1.23] | 0.99 |
| HP-hMG + rFSH | 1 | 64 | 1.00 [0.36, 2.77 |  |
| rLH+rFSH | 2 | 222 | 1.06 [0.29, 3.84] |  |
| **Age>35 years** | 4 | 542 | 1.21 [0.64, 2.27] |  |
| HP-hMG | 1 | 127 | 1.50 [0.94, 2.39] | 0.48 |
| rLH+rFSH | 3 | 415 | 0.97 [0.32, 2.97] |  |
| **GnRH agonist protocol** | 8 | 2835 | 1.08 [0.86, 1.35] |  |
| HP-hMG | 3 | 1487 | 1.23 [1.00, 1.51] | 0.53 |
| HP-hMG + rFSH | 1 | 64 | 1.00 [0.36, 2.77] |  |
| rLH+rFSH | 4 | 1284 | \|  \| 0.92 [0.57, 1.48] \| \| --- \| --- \| |  |
| **GnRH antagonist protocol** | 6 | 1910 | 1.05 [0.93, 1.20] |  |
| HP-hMG | 3 | 1428 | 1.08 [0.95, 1.22] | 0.61 |
| HP-hMG + rFSH | 1 | 152 | 0.97 [0.43, 2.20] |  |
| rLH+rFSH | 2 | 330 | 0.51 [0.11, 2.32] |  |

| **Clinical/ongoing pregnancy** |  |  |  |  |
| --- | --- | --- | --- | --- |
| **Ovarian reserve** | 11 | 3672 | 1.09 [0.91, 1.30] |  |
| Poor reserve_HP-hMG | 1 | 155 | 0.51 [0.25, 1.04] | 0.32 |
| Poor reserve_HP-hMG + rFSH | 2 | 310 | 1.02 [0.66, 1.57] |  |
| Poor reserve_rLH+rFSH | 5 | 1450 | 1.13 [0.83, 1.54] |  |
| Normal reserve_HP-hMG | 1 | 313 | 1.24 [0.52, 2.97] |  |
| Normal reserve_HP-hMG + rFSH | 1 | 610 | 1.28 [0.97, 1.67] |  |
| High reserve_HP-hMG | 3 | 834 | 1.06 [0.67, 1.68] |  |
| **Age≤35 years** | 14 | 4401 | 1.12 [1.03, 1.23] |  |
| HP-hMG | 3 | 1987 | 1.15 [1.00, 1.32] | 0.67 |
| HP-hMG + rFSH | 2 | 674 | 1.20 [0.93, 1.56] |  |
| rLH+rFSH | 9 | 1740 | 1.08 [0.94, 1.23] |  |
| **Age>35 years** | 14 | 2056 | 1.01 [0.85, 1.21] |  |
| HP-hMG | 2 | 239 | 0.90 [0.38, 2.11] | 0.13 |
| HP-hMG + rFSH | 1 | 158 | 1.04 [0.62, 1.75] |  |
| rLH+rFSH | 11 | 1659 | 1.00 [0.81, 1.24] |  |
| **GnRH agonist protocol** | 8 | 3987 | 1.05 [0.94, 1.19] |  |
| HP-hMG | 4 | 2214 | 1.12 [0.97, 1.29] | 0.41 |
| HP-hMG + rFSH | 1 | 64 | 1.00 [0.36, 2.77] |  |
| rLH+rFSH | 3 | 1709 | 0.94 [0.77, 1.16] |  |
| **GnRH antagonist protocol** | 7 | 2813 | 1.03 [0.92, 1.16] |  |
| HP-hMG | 3 | 1648 | 1.11 [0.96, 1.28] | 0.23 |
| HP-hMG + rFSH | 1 | 152 | 0.97 [0.45, 2.11] |  |
| rLH+rFSH | 3 | 1013 | 0.89 [0.73, 1.09] |  |

| **OHSS** |  |  |  |  |
| --- | --- | --- | --- | --- |
| **Ovarian reserve** | 5 | 2668 | 0.57 [0.40, 0.83] |  |
| Poor reserve_rLH+rFSH | 1 | 939 | 3.10 [0.13, 75.83] | 0.45 |
| Normal reserve_HP-hMG | 1 | 157 | 0.99 [0.06, 15.51] |  |
| Normal reserve_HP-hMG + rFSH | 1 | 610 | 0.91 [0.39, 2.11] |  |
| High reserve_HP-hMG | 2 | 962 | 0.50 [0.32, 0.79] |  |
| **Age<=35 years** | 7 | 2805 | 0.67 [0.44, 1.03] |  |
| HP-hMG | 4 | 1748 | 0.74 [0.37, 1.47] | 0.52 |
| HP-hMG + rFSH | 1 | 610 | 0.91 [0.39, 2.11] |  |
| rLH+rFSH | 2 | 447 | 0.41 [0.10, 1.75] |  |
| **Age>=35 years** | 2 |  |  |  |
| rLH+rFSH | 2 | 423 | 1.32 [0.25, 6.96] | NA |
| **GnRH agonist protocol** | 13 | 6917 | 1.04 [0.66, 1.65] |  |
| HP-hMG | 5 | 2727 | 0.87 [0.52, 1.46] | 0.56 |
| HP-hMG + rFSH | 2 | 1174 | 0.60 [0.24, 1.50] |  |
| rLH+rFSH | 7 | 3016 | 1.04 [0.66, 1.65] |  |
| **GnRH antagonist protocol** | 7 | 2813 | 0.61 [0.38, 0.97] |  |
| HP-hMG | 4 | 1813 | 0.54 [0.34, 0.86] | 0.24 |
| rLH+rFSH | 3 | 1000 | 1.25 [0.34, 4.62] |  |

| **Number of oocytes** | **Studies** | **Participants** | **Mean Difference**  **(IV, Random, 95% CI)** | **Test for subgroup differences**  ***p*** |
| --- | --- | --- | --- | --- |
| **Ovarian reserve** | 9 | 2851 | -1.07 [-2.18, 0.05] |  |
| Poor reserve_HP-hMG + rFSH | 2 | 319 | 0.23 [-0.30, 0.77] | <0.001 |
| Poor reserve_rLH + rFSH | 3 | 1141 | 0.06 [-0.78, 0.90] |  |
| Normal reserve_HP-hMG | 1 | 104 | -1.40 [-4.38, 1.58] |  |
| Normal reserve_HP-hMG + rFSH | 1 | 609 | -0.60 [-1.57, 0.37] |  |
| High reserve_HP-hMG | 2 | 678 | -7.09 [-8.71, -5.47] |  |
| **Age≤35 years** | 13 | 4033 | -0.42 [-1.43, 0.58] |  |
| HP-hMG | 4 | 1572 | -2.28 [-5.46, 0.91] | 0.33 |
| HP-hMG + rFSH | 2 | 669 | 0.21 [-1.63, 2.05] |  |
| rLH+rFSH | 8 | 1792 | 0.23 [-0.67, 1.14] |  |
| **Age>35 years** | 11 | 2271 | -0.46[-1.21, 0.30] |  |
| HP-hMG + rFSH | 1 | 164 | 0.35 [-0.32, 1.02] | 0.08 |
| rLH+rFSH | 10 | 2107 | -0.56 [-1.36, 0.23] |  |
| **GnRH agonist protocol** | 27 | 8045 | -0.39 [-0.83, 0.05] |  |
| HP-hMG | 5 | 2480 | -1.20 [-2.42, 0.01] | 0.25 |
| HP-hMG + rFSH | 3 | 1243 | 0.11 [-0.87, 1.09] |  |
| rLH+rFSH | 20 | 4322 | -0.30 [-0.82, 0.22] |  |
| **GnRH antagonist protocol** | 16 | 3482 | -4.74 [-9.98, 0.51] |  |
| HP-hMG | 6 | 1909 | -2.75 [-5.27, -0.23] | 0.07 |
| HP-hMG + rFSH | 1 | 147 | 0.00 [-0.94, 0.94] |  |
| rLH+rFSH | 9 | 1426 | -6.42 [-16.66, 3.82] |  |
| **Number of MII oocytes** |  |  |  |  |
| **Ovarian reserve** | 9 | 2714 | -1.27 [-2.43, -0.21] |  |
| Poor reserve_HP-hMG + rFSH | 4 | 1155 | -0.14 [-0.40, 0.12] | <0.001 |
| Normal reserve_HP-hMG | 2 | 272 | -0.93 [-1.72, -0.14] |  |
| Normal reserve_HP-hMG + rFSH | 1 | 609 | -0.80 [-1.67, 0.07] |  |
| High reserve_HP-hMG | 2 | 678 | -5.84 [-7.08, -4.60] |  |
| **Age≤35 years** | 10 | 2104 | -0.08 [-1.62, 1.46] |  |
| HP-hMG | 3 | 797 | -2.13 [-6.58, 2.33] | 0.41 |
| HP-hMG + rFSH | 2 | 669 | 0.23 [-1.92, 2.38] |  |
| rLH+rFSH | 5 | 638 | 0.99 [-0.53, 2.51] |  |
| **Age>35 years** | 6 |  |  |  |
| rLH+rFSH | 6 | 815 | -1.08 [-1.50, -0.66] | NA |
| **GnRH agonist protocol** | 16 | 3836 | -0.09 [-0.61, 0.43] |  |
| HP-hMG | 1 | 74 | 1.10 [-0.51, 2.71] | 0.32 |
| HP-hMG + rFSH | 2 | 669 | 0.23 [-1.92, 2.38] |  |
| rLH+rFSH | 13 | 3093 | -0.20 [-0.77, 0.37] |  |
| **GnRH antagonist protocol** | 14 | 2575 | -1.12 [-2.09, -0.15] |  |
| HP-hMG | 6 | 1353 | -2.74 [-4.65, -0.83] | 0.02 |
| HP-hMG + rFSH | 1 | 147 | 0.20 [-0.58, 0.98] |  |
| rLH+rFSH | 7 | 1075 | -0.07 [-0.77, 0.63] |  |

Supplementary Figure 1 Risk of Bias of Live Birth/Ongoing Pregnancy

Supplementary Figure 2 Risk of Bias of Number of Oocytes/MII Oocytes

Supplementary Figure 3 Risk of Bias of OHSS

**Certainty of evidence**

Supplementary Table 4 Summary of Findings Table Comparison 1: Gonadotropins with LH Activity vs rFSH

| Outcomes | № of participants (studies) | Certainty assessment | | | | | Certainty of the evidence (GRADE) | Relative effect (95% CI) | Anticipated absolute effects | |
| --- | --- | --- | --- | --- | --- | --- | --- | --- | --- | --- |
|  |  | Risk of bias | Inconsistency | Indirectness | Imprecision | Other considerations |  |  | Risk with rFSH | Risk difference with LH activity |
| Live birth | 5096 (17 RCTs) | not serious | not serious | not serious | serious^a^ | none | ⨁⨁⨁◯ Moderate^a^ | **RR 1.07** (0.96 to 1.18) | 218 per 1.000 | **13 more per 1.000** (9 fewer to 39 more) |
| Pregnancy assessed with: Clinical or ongoing pregnancy | 12357 (46 RCTs) | not serious | not serious | not serious | serious^a^ | none | ⨁⨁⨁◯ Moderate^a^ | **RR 1.02** (0.96 to 1.09) | 252 per 1.000 | **8 more per 1.000** (10 fewer to 25 more) |
| Number of oocytes assessed with: PP analysis | 11734 (46 RCTs) | serious^b^ | serious^c^ | not serious | not serious | none | ⨁⨁◯◯ Low^b,c^ | - | The mean number of oocytes was **9.77** | MD **0.50 lower** (0.88 lower to 0.12 lower) |
| Number of MII oocytes  assessed with: PP analysis | 6530 (30 RCTs) | serious^b^ | very serious^c^ | not serious | not serious | none | ⨁◯◯◯ Very low^b,c^ | - | The mean number of MII oocytes was **7.47** | MD **0.49 lower** (0.93 lower to 0.05 lower) |
| OHSS assessed with: Composite of OHSS cases and cycles cancelled due to risk of OHSS | 9543 (19 RCTs) | not serious | not serious^d^ | not serious | serious^a^ | none | ⨁⨁⨁◯ Moderate^a,d^ | **RR 0.80** (0.61 to 1.03) | 40 per 1.000 | **8 fewer per 1.000** (16 fewer to 1 more) |
| ***The risk in the intervention group** (and its 95% confidence interval) is based on the assumed risk in the comparison group and the **relative effect** of the intervention (and its 95% CI). **CI:** confidence interval; **MD:** mean difference; **RR:** risk ratio  a. Confidence intervals are compatible with no effect and with a clinically relevant effect  b. Unclear to high risk of bias due to randomization process, deviations from the intervention and missing outcome data. A sensitivity ITT analysis, which could overcome the missing outcome data, reached similar conclusions to the main analysis.  c. Extreme heterogeneity (I2 > 80%)  d. Minimal heterogeneity (I2 = 17.1%) | | | | | | | | | | |

Supplementary Table 5 Summary of Findings Table Comparison 2: HP-HMG (± rFSH) vs rLH + rFSH

| Outcomes | № of participants (studies) | Certainty assessment | | | | | Certainty of the evidence (GRADE) | Relative effect (95% CI) | Anticipated absolute effects | |
| --- | --- | --- | --- | --- | --- | --- | --- | --- | --- | --- |
|  |  | Risk of bias | Inconsistency | Indirectness | Imprecision | Other considerations |  |  | Risk with rLH+rFSH | Risk difference with HP-hMG + rFSH |
| Live birth | 175 (2 RCTs) | serious^a^ | serious^b^ | not serious | serious^c^ | none | ⨁◯◯◯ Very low^a,b,c^ | **RR 1.35** (0.26 to 6.99) | 170 per 1.000 | **60 more per 1.000** (126 fewer to 1.021 more) |
| Clinical pregnancy | 234 (2 RCTs) | not serious | serious^b^ | not serious | serious^c^ | none | ⨁⨁◯◯ Low^b,c^ | **RR 1.05** (0.39 to 2.85) | 264 per 1.000 | **13 more per 1.000** (161 fewer to 489 more) |
| Number of oocytes assessed with: PP analysis | 251 (2 RCTs) | not serious | serious^b^ | not serious | serious^c^ | none | ⨁⨁◯◯ Low^b,c^ | **-** | The mean number of oocytes was **9.4** | MD **1.89 lower** (5.65 lower to 1.87 higher) |
| Number of MII oocytes  assessed with: PP analysis | 140 (1 RCT) | serious^d^ | not serious | not serious | very serious^c^ | none | ⨁◯◯◯ Very low^c,d^ | **-** | The mean number of MII oocytes was **8.0** | MD **1.33 higher** (0.49 lower to 3.15 higher) |
| OHSS assessed with: Composite of OHSS cases and cycles cancelled due to risk of OHSS | 391 (4 RCTs) | serious^a^ | serious^e^ | not serious | not serious | none | ⨁⨁◯◯ Low^a,e^ | not estimable |  |  |
| ***The risk in the intervention group** (and its 95% confidence interval) is based on the assumed risk in the comparison group and the **relative effect** of the intervention (and its 95% CI).  **CI:** confidence interval; **MD:** mean difference; **RR:** risk ratio  a. Unclear or high risk of bias due to concerns on randomization process  b. Extreme heterogeneity (I2 >80%)  c. Confidence interval compatible with no effect and with relevant effect  d. Unclear risk of bias due to some concerns in randomization, deviations from intended interventions, missing outcome data and selection of the reported result.  e. Moderate heterogeneity (I2 = 61.9%) | | | | | | | | | | |
